# The (Null) Effects of Happiness on Affective Polarization, Conspiracy Endorsement, and Deep Fake Recognition: Evidence from Five Survey Experiments in Three Countries

**DOI:** 10.1007/s11109-021-09701-1

**Published:** 2021-03-18

**Authors:** Xudong Yu, Magdalena Wojcieszak, Seungsu Lee, Andreu Casas, Rachid Azrout, Tomasz Gackowski

**Affiliations:** 1 Department of Communication, University of California, Davis, Davis, CA 95616 USA; 2 Amsterdam School of Communication Research, University of Amsterdam, Amsterdam, The Netherlands; 3 Department of Communication Science, Free University of Amsterdam, Amsterdam, The Netherlands; 4 Department of Communication Science, University of Amsterdam, Amsterdam, The Netherlands; 5 Department of Social Communication and Public Relations, University of Warsaw, Warsaw, Poland

**Keywords:** Happiness, Affective polarization, Misinformation, Conspiracy endorsement, Deep fake, Misattribution of affect

## Abstract

Affective polarization is a key concern in America and other democracies. Although past evidence suggests some ways to minimize it, there are no easily applicable interventions that have been found to work in the increasingly polarized climate. This project examines whether irrelevant factors, or incidental happiness more specifically, have the power to reduce affective polarization (i.e., misattribution of affect or “carryover effect”). On the flip side, happiness can minimize systematic processing, thus enhancing beliefs in conspiracy theories and impeding individual ability to recognize deep fakes. Three preregistered survey experiments in the US, Poland, and the Netherlands (total *N* = 3611) induced happiness in three distinct ways. Happiness had no effects on affective polarization toward political outgroups and hostility toward various divisive social groups, and also on endorsement of conspiracy theories and beliefs that a deep fake was real. Two additional studies in the US and Poland (total *N* = 2220), also induced anger and anxiety, confirming that all these incidental emotions had null effects. These findings, which emerged uniformly in three different countries, among different partisan and ideological groups, and for those for whom the inductions were differently effective, underscore the stability of outgroup attitudes in contemporary America and other countries.

**Supplementary Information:**

The online version contains supplementary material available at 10.1007/s11109-021-09701-1.

Affective polarization is a key concern in various countries. In the US, partisans increasingly dislike and distrust their political opponents (Iyengar and Westwood 2015; Iyengar et al. 2019; Mason 2018), discrimination against one’s partisan outgroup trumps that based on other social cleavages in several other western contexts (e.g., Westwood et al. 2018), and newer democracies in Central Europe are polarizing along support or opposition toward their authoritarian governments (Górska 2019). Hostility toward the political opposition has concrete implications for the democratic system, thwarting consensus, paralyzing governance, and leading partisans to distrust the government run by the opposing party (Hetherington and Rudolph 2015), as well as for citizens’ non-political attitudes and behaviors, such as decreasing romantic relationships and friendships across party lines (Chopik and Motyl 2016; Huber and Malhotra 2017; Nicholson et al. 2016) and influencing decisions in the labor market (Gift and Gift 2015; see Iyengar et al. 2019 for a review).

Despite its negative outcomes, there is limited evidence on how to minimize affective polarization. Past research suggests potential strategies, such as correcting misperceptions about outparty supporters (Ahler and Sood 2018) or enhancing the sense of common national identity (Levendusky 2018a). Given the increasing partisan animosity (Pew 2019a) and the widening partisan divide over key issues in the US (Newport and Dugan 2017), it is even more difficult to attenuate interparty hostilities, as suggested by more recent work (see e.g., Levendusky 2018b; Wojcieszak and Warner 2020 for largely indirect effects). Also, although affective polarization is on the rise internationally (Fomina 2019; Górska 2019; Silva 2018), no work tests easily applicable interventions outside the US.

As part of the continuous scholarly effort, this project proposes an innovative solution to mitigate affective polarization, namely making people feel happy. Emotions are critically important to politics (e.g., Marcus et al. 2000). However, most work to date has focused on the effects of negative emotions and of integral emotions on political attitudes and behaviors (e.g., Valentino et al. 2008; Vasilopoulos et al. 2019). Positive emotions in general—and incidental happiness in particular—are not studied extensively in political science literature (or in the context of politics). Also, although emotions are the key underpinning of affective polarization (Iyengar et al. 2012), there is no work investigating their potential to minimize interparty hostility.

Bridging social psychology and political science, we examine whether incidental happiness has the power to improve attitudes toward political outgroups (i.e., misattribution of affect or “spillover effect”; Schwarz and Clore 1983). Because affective polarization is heightened by negative feelings for the outparty (Iyengar et al. 2012) and people tend to evaluate the target more positively when they feel happy (Schwarz 2000), triggering happiness unrelated to the political context should improve citizens’ evaluations of their opponents, thereby reducing affective polarization. This solution is also versatile: if found effective, social media platforms could easily feature “feel good” messages or images to users, which overtime could decrease interparty animus. This exploration adds to the literature on the effects of irrelevant, non-political events on political attitudes and behaviors (Achen and Bartels 2017; Busby et al. 2017), as we note below.

In addition to testing whether experimentally induced happiness has the power to spill over to outgroup attitudes, we also examine whether it can have unintended consequences. Extant work often neglects the fact that minimizing one bad outcome may produce negative “side-effects.” For example, priming common American identity lowers affective polarization toward outparty members (Levendusky 2018a) but also enhances negative attitudes toward undocumented immigrants (Wojcieszak and Garrett 2018). Similarly, corrective information, although sometimes effective (Bode and Vraga 2018), may suppress political expression (Lawrence and Estow 2017). The potential side effects of various interventions are not known because scholars typically focus on minimizing one bad outcome at a time. Therefore, we examine whether making people feel happy can reduce affective polarization while also increasing people’s endorsement of conspiracy theories and reducing the ability to recognize a deep fake by prompting reliance on heuristic information processing (Chaiken 1980).

To enhance the robustness of the findings, we test our theoretical predictions in three distinct countries: the US (a two-party system, where most of the research on affective polarization originates), the Netherlands (an established multi-party Western democracy that is known for its consensus-based political model), and Poland (an increasingly polarized post-communist democracy in Central Europe).

Using three pre-registered survey experiments in the three countries (total *N* = 3611), we find no evidence that incidental happiness has any effect on affective polarization toward country-specific political outgroups and divisive social groups, and also on conspiracy endorsement as well as beliefs that a deep fake video is real. Two additional not pre-registered studies in the US and Poland (total *N* = 2220) also induced anger and anxiety, emotions that may exacerbate affective polarization when carried through to subsequent judgments. All these incidental emotions had null effects on the core outcomes. Our findings, which emerged uniformly across the countries, for various outgroups, among different political and ideological sub-samples, for those who have different levels of political identity strength and political interest, and for those for whom the emotion inductions were differently effective, underscore the immovable nature of affective polarization and the persistence of misinformed beliefs.

## Incidental Happiness and Affective Polarization

Affective polarization refers to the tendency of partisans to increasingly dislike, even hate, their political opposition, impute negative traits to their opponents, and feel displeasure at the potential of interacting with outparty members (Iyengar et al. 2012). Although some scholars conceptualize affective polarization as the difference between ingroup favoritism and outgroup animosity (e.g., Iyengar and Westwood 2015; Rogowski and Sutherland 2016), other work defines it as outgroup animosity only (e.g., Druckman et al. 2020; Iyengar et al. 2012, 2019; Levendusky 2018a), partly because the increase in the ingroup versus outgroup difference is mainly driven by heightened negative affect toward the opposition (Druckman and Levendusky 2019; Iyengar et al. 2019).

This negative affect arises from the fact that partisans in the US increasingly treat identification with a political party as one of their core social identities (Mason 2018), as do citizens in other divided democracies (Westwood et al. 2018). Identification with one group (e.g., one’s political camp, be it a party or an ideological group) leads individuals to place others into ingroups (in-partisans or fellow ideologues) and outgroups (supporters of the outparty or opposing ideologues; Tajfel and Turner 1979), resulting in positive feelings toward ingroup members and under certain conditions (e.g., power politics) negative feelings toward outgroup members (Brewer 1999).

In the US, there is strong empirical support for the observations of growing interparty hostility. “Democrats and Republicans both say that the other party’s members are hypocritical, selfish, and closed-minded, and they are unwilling to socialize across party lines, or even to partner with opponents in a variety of other activities” (Iyengar et al. 2019, p. 130). Parallel evidence from international contexts is limited. Some work shows that partisan discrimination is greater than that based on other social cleavages in old and newer democracies (Carlin and Love 2018; Górska 2019; Martini and Torcal 2019; Westwood et al. 2018), and that trends in affective polarization over the past several decades vary across countries, with the US experiencing larger increase in polarization than most other western countries (Boxell et al. 2020; Gidron et al. 2020).

The high levels of affective polarization and its deleterious democratic effects make it ever more important to develop theoretically-driven and easily implementable mitigation strategies. Toward this end, we build on the literature on irrelevant effects, which emphasizes the subtle power of non-political factors in shaping political attitudes and behaviors. For instance, a win of a local football team before election can increase the incumbent vote share (Healy et al. 2010) and influence presidential approval, likely by affecting individuals’ moods (Busby et al. 2017; see also, Huber et al. 2012; Achen and Bartels 2017 for political outcomes of lotteries, floods, or shark attacks). We focus not on uncontrolled events but rather on clearly apolitical and ostensibly politically irrelevant induced affective states.

We draw on the classic theorizing and research in social psychology on the misattribution of affect (Payne et al. 2010; Schwarz and Clore 1983) and the spillover or carryover effect (De Hooge et al. 2014; Lerner et al. 2004). Extensive evidence suggests that incidental emotions—emotions “elicited in past, normatively unrelated situations” (Small and Lerner 2008, p. 151)—can impact present judgements and attitudes. For example, happiness triggered by sunny weather increases overall life satisfaction (Schwarz and Clore 1983), sadness produced by watching a movie clip encourages people to increase buying prices but reduce selling prices for the same object (Lerner et al. 2004), and happiness stimulated by listening to upbeat music makes people choose riskier lotteries (Schulreich et al. 2014).

There are two mechanisms through which incidental emotions could carry over and influence attitudes toward irrelevant topics and objects. First, individuals are more likely to recall mood-congruent thoughts and information from memory (e.g., Isen et al. 1978; Snyder and White 1982). In addition, the feelings-as-information theory (Schwarz 2012; Schwarz and Clore 1983) suggests that people attend to their current feelings as a source of information when evaluating the target. That is, people are likely to attribute their feelings elicited in the past by potentially irrelevant events or information to the target at hand and then mix up these feelings with their responses and attitudes to the target (Schwarz 2000, 2012).^1^

This work has rarely been applied to the sociopolitical context despite its immense potential and implications (but see Small and Lerner 2008; Ottati and Isbell 1996; Webster 2018). Inducing happiness before citizens make political decisions or evaluate political objects may influence their cognitions, attitudes, and/or behaviors. Here, we speculate that incidental happiness can attenuate affective polarization. When individuals are asked to evaluate the political outgroup while feeling good, they are more likely to respond positively because they recall more positive thoughts and because their happy state “spills over” to the outgroup. Our first overarching theoretical expectation is that *participants induced to feel happy will exhibit lower affective polarization than participants in the control condition (H1).*

In testing this effect, this project extends the work on affective polarization from its North American cradle to other political contexts. In the US, because the two major parties dominate the political arena, the distinction between partisan ingroup (i.e., a Democratic voter for a Democrat) and outgroup (i.e., a Republican voter for a Democrat) is straightforward. However, most other countries are multiparty systems, where partisan in- and out-groups are not structured around a dichotomy between opposing sides, strategic voting is common, and also partisan volatility is greater and the levels of partisan identification are lower than in the US (Bankert et al. 2017; Dalton 2014; Dalton and Weldon 2007; Huddy et al. 2018). There, affective polarization may be rooted in the broader ideological divide between the left and the right, which has traditionally split the electorates in Europe (e.g., Markowski 1997; see also Nicholson et al. 2018), or around other contemporary conflicts.

We test our predictions regarding incidental happiness reducing affective polarization in the US and two other distinct multi-party systems. We focus on the Netherlands, a stable and established Western European democracy that has relatively low levels of partisanship and low levels of partisan identity and where ideology matters to citizens’ cognitions and behaviors (see Huddy et al. 2018). The left–right divide is one of the most fundamental cleavages in Dutch politics (Andeweg and Irwin 2014; Pennings and Keman 2003) and polarization based on ideological identity has increased among the Dutch public in recent years (Silva 2018). We also examine Poland, a post-communist country with less established democratic traditions, where the current populist government “has managed to reformulate the main dividing line of political life and turn its followers and critics into two unyielding hostile camps” (Fomina 2019, p. 126): its supporters and those mobilized against the government’s policies and rhetoric. Although these camps are sometimes described using traditional socioeconomic categories such as wealth, education, and urban versus rural residence, these are secondary to the political and ideological orientations of the government supporters and opponents (e.g., religious and exclusivist view of Poland’s national identity versus pro-European and cosmopolitan outlook; Fomina 2019). These opposing camps have as or more negative attitudes toward their political opponents than toward various groups traditionally disliked in the (largely Catholic) Poland (i.e. Jews, Muslims, refugees, and sexual minorities; Górska 2019) and affective polarization has reached an unprecedented level, undermined trust in public institutions, and damaged the quality of policy processes (see Fomina 2019).

Furthermore, to offer a robust test of our hypothesis, we also test the effects of incidental happiness on affective polarization toward a range of political targets. For one, we examine the effects of incidental happiness on feelings toward political parties and also their voters (inasmuch as American partisans tend to evaluate the outparty more negatively than its supporters; Druckman and Levendusky 2019). Second, to shed light on the nuances of political outgroups in the three countries, we also test affective polarization toward one’s ideological outgroup (e.g., those who are politically liberal for a conservative) and—in Poland—toward government opponents [supporters] for the supporters [opponents]. Further, if our theoretical argument is correct, incidental happiness should also improve attitudes toward other social groups that are divisive and typically disliked by the left or the right, and so we examine its effects on feelings toward several groups at the center of political conflicts in the US and internationally: immigrants, feminists, and the far right.

## Incidental Happiness, Conspiracy Endorsement, and Deep Fake Recognition

The last objective of this project is to test whether the proposed remedy to affective polarization may produce unintended consequences. We suspect that incidental happiness can minimize systematic information processing, thus enhancing the extent to which individuals endorse misinformed beliefs. Happiness may signal a safe and benign situation, thus indicating that careful and effortful evaluation of a message is not necessary (Schwarz 2012). Extensive research shows that individuals who feel good or are induced to feel positive emotions rely on heuristics and pre-existing knowledge and lack the motivation to systematically process incoming information (e.g., Bodenhausen et al. 1994; Bohner et al. 1992). This robust finding applies to such ostensibly non-political topics as an increase in student services fees (Bless et al. 1990) and to such divisive issues as prejudice against African Americans (Park and Banaji 2000). Germane to our project, incidental happiness also decreases individuals’ skepticism and their ability to identify deceitful suspects (Forgas and East 2008). In short, happiness should make people less likely to scrutinize political information. Therefore, our second overarching expectation is that *partisans who are feeling happy will be more likely than participants in the control condition to endorse conspiracy theories* (*H2a) and less likely to recognize a deep fake as fake* (*H2b).*

We test these preregistered hypotheses across five survey experiments on non-probability-based, but representative on key census demographics, samples in the three countries in order to assess the robustness and the generalizability of the effects. Across the studies, we varied the ways in which incidental happiness was induced (i.e., photos, writing task, questions), the relevant control conditions (i.e., a general control group versus one for each happiness treatment), the measurement of the manipulation checks (i.e., positive affect scale from the Positive and Negative Affect Schedule, self-assessment manikin scale, coding open-ended responses) and also the emotional comparison (i.e., eliciting two negative emotions in the last two studies). We find no evidence that incidental happiness influenced affective polarization toward various political and social groups, conspiracy endorsement, and believing that a deep fake is real. Below, we report the results for the various political outgroups combined into an aggregate index of affective polarization (see also, Druckman et al. 2020) and Appendix H details the (similarly null) effects of happiness on affective polarization toward the individual outgroups.

### Study 1 and 2: Design and Measures

The first two pre-registered survey experiments (see https://osf.io/7dp4z; see Appendix A for the report and explanations of the deviations from the pre-registration), carried out in the US (Study 1) and Poland (Study 2), aimed to test the two overarching hypotheses and also the various ways in which incidental happiness can be induced in survey experimental settings. Because writing about one’s feelings, states, and experiences is more immersive and engages the self to a greater extent than mere exposure to images and (likely) than answering survey questions (see Pingree 2007), we expected that the *happy writing* task treatment, in which participants are asked to describe something that made them feel happy and positive, will exert stronger effects on the tested outcomes than *happy photos* treatment, which was predicted to exert stronger effects than *happy questions* treatment, in which happiness was elicited by asking participants to answer a series of questions about things they like. The treatments had been piloted (see Appendix B for details).

Study 1 (the US) was conducted by Dynata (former *Survey Sampling International*) and Study 2 (Poland) by Panel Ariadna (a Polish public opinion polling company with a high-quality invitation-only panel) in May 2019. In order for our samples to approximate the US/Polish populations, we set quotas for age, gender, education (and additionally for ethnicity in the US and the size of municipality in PL), and political predispositions (partisanship in the US and support-opposition for the current government in PL, i.e., the core axis of polarization). A total of 1370 participants completed Study 1 and 1229 completed Study 2. Respondents who failed the attention check (*n* = 72 in each study) and those who completed the study in under 48% of the median time, a standard recommended by Dynata (Study 1: 4 min 14 s, *n* = 69; Study 2: 6 min 30 s, *n* = 57), were excluded. Also, across both experiments those who had no clear political leanings, as assessed at the pretest (see below), were filtered out automatically (i.e., those who identified as partisan independents close to neither political party in the US, and as neither supporters nor opponents of the current government in Poland). The final samples include 1248 participants (the US) and 1109 participants (PL; see Appendix C for demographics for all the studies).^2^

Question wording, descriptive statistics for all the items, and the scaling statistics for all the scales for both studies (as well as the remaining studies) are presented in Appendix D. Across both experiments, participants first reported their political predispositions (ideology in both countries, partisanship in the US, most and least liked political party in PL, and support/ opposition toward the government in PL). The pretest also measured people’s current emotional state on four 7-point semantic differential scales (sad-happy, bad-good, tired-rested, tense-relaxed) to account for baseline emotions, political interest, and political identity strength (e.g., “I often think of myself as a Democrat /Republican” in the US or “…as a government supporter/opponent” in PL; Sniderman et al. 2004), both of which were predicted to influence the treatment effects, such that the more politically interested and the strong political identifiers may be less influenced by the treatments (these heterogeneous effects were part of the preregistered analysis plan, and we additionally did exploratory analysis by one’s partisanship and ideology).

Participants were then randomly assigned to one of the four treatment groups: *happy writing* (describing something that made them feel joyful and good, with several examples provided; US: *n* = 309, Poland: *n* = 272; adapted from Valentino et al. 2011), *happy questions* (answering six questions about things they like, e.g., “What is your favorite song?”; US: *n* = 315, Poland: *n* = 297),^3^*happy photos* (seeing five photos, e.g., a photo of a kitten and a puppy; US: *n* = 314, Poland: *n* = 245), or the control (no information/images; US: *n* = 310, Poland: *n* = 295). Randomization was successful apart from a significant difference on age in Study 2. We thus enter age as a covariate in our models. See Appendix E for details about randomization check for all the studies. Appendix F presents all the stimuli used in Study 1 and 2.

The posttest again assessed participants’ current feelings on the four semantic differential scales and using the 10-item Positive Affect scale from PANAS (Watson et al. 1988). Afterwards, we measured affective polarization using four classic indicators (i.e., feeling thermometers, social distance, trait ratings, and outgroup trust), with the outgroups adapted to each country (see Levendusky 2018a; Wojcieszak and Garrett 2018). As aforementioned, although some scholars compute difference scores between ingroup and outgroup feelings (e.g., Garrett et al. 2014; Wojcieszak et al. 2020), others focus exclusively on attitudes toward the political outgroup as indicators of affective polarization (e.g., Druckman et al. 2020; Levendusky 2018b). Because we treat affective polarization as hostility toward political opponents, the latter operationalization was adopted in all five studies.

The posttests contained four classical measures of affective polarization asking about various political groups. First, on the standard 101-point feeling thermometers, American partisans rated the Democratic Party, the Republican Party, supporters of the Democratic Party, supporters of the Republican Party, and those who are politically liberal and conservative. The Polish participants rated their favorite party, the least favorite party, supporters of the favorite party, supporters of the least favorite party, supporters and opponents of the government, and also those who are politically left and right.^4^ We relied on participants’ pretest partisanship and ideology in the US and PL and also their opposition or support toward the government in PL to identify their respective political outgroup (e.g., those politically conservative for a liberal, or government supporters for an opponent), and use these outgroup indicators in the final measure.^5^^,^ 6 The remaining affective polarization indicators asked about the core political outgroup only. Social distance tapped into the comfort people would feel having an outparty supporter (in the US) and a supporter/opponent of the government (in Poland) as a work colleague, a close relative by marriage, and a neighbor. Trait ratings were used to evaluate outparty supporters (in the US) and supporters/opponents of the government (in PL) as intelligent, honest, open-minded, mean, hypocritical, and selfish (with the negative items reverse coded). Lastly, we measured outgroup trust using four items about the political outgroup (e.g., “Most Democrats/Republicans (US), supporters/opponents of the current government (PL) are basically honest”).

The preregistration specified analyses of each individual affective polarization indicator (e.g., feeling thermometers, social distance, trait ratings, and outgroup trust), also differentiating between outgroup supporters and outgroup elites. For all five studies, we estimated these models and detail the results in Appendix H. Here, for parsimony and following recent work (e.g., Druckman et al. 2020), we present the results for the aggregate index of affective polarization created by first rescaling all the *outgroup items* (e.g., feeling thermometers toward the outparty, its supporters, and out-ideologues, social distance, trait ratings, and outgroup trust in Study 1) between 0 and 1 and later averaging the measures (*M* = 0.37, SD = 0.18, *α* = 0.84, Study 1; *M* = 0.31, SD = 0.15, *α* = 0.79, Study 2).

Furthermore, in both countries, participants evaluated immigrants, feminists, and Neo-Nazis on the 101-point feeling thermometers. In Poland, an additional feeling thermometer tapped into feelings toward nationalists. We used these indicators in separate pre-registered models to assess whether our treatments had the power to lower hostility toward these social groups.

Because the affective polarization batteries, in which people are asked to think about their political opponents, may “erase” whatever happy feelings produced by the treatments, we re-introduced these treatments subtly before measuring conspiracy endorsement and deep fake recognition (e.g., those in the *happy questions* group saw their answers to the happiness-eliciting questions). We tapped conspiracy endorsement asking about the extent to which participants agreed with six statements pointing to various conspiracy theories (e.g., “The government is deliberately hiding the truth about how many immigrants really live in the country” or “The idea of man-made global warming is a hoax that was invented to deceive people”). To test treatment effects on deep fake recognition, we showed the participants a deep fake video made by the Belgian socialist party featuring Donald Trump speaking about global warming, and with professional subtitles in Polish for Study 2.^7^ On a 5-point scale, participants then reported how fake or real they thought the video was. Feeling thermometers toward social groups, conspiracy endorsement, and deep fake recognition were rescaled between 0 and 1.

### Study 1 and 2: Results

We first assessed whether our happiness treatments were effective. Participants’ responses to the 10 items from the PANAS scale did not significantly differ across the groups [Study 1: *F*(3, 1244) = 1.11, *p* = 0.342; Study 2: *F*(3, 1105) = 0.94, *p* = 0.422]. We find some predicted effects on the semantic differential scales: In the US, the happy photos treatment (*M* = 5.86, SD = 1.28) increased happy/good feelings significantly more than the control [*M* = 5.80, SD = 1.35, *t*(1243) = 2.80, *p* = 0.005], yet the happy questions [*M* = 5.64, SD = 1.36, *t*(1243) = − 0.30, *p* = 0.767] and the happy writing [*M* = 5.67, SD = 1.34, *t*(1243) = 1.59, *p* = 0.111] treatments were indistinguishable from the control.^8^ In Poland, happy photos [*M* = 5.24, SD = 1.42, *t*(1105) = 2.10, *p* = 0.036], but not the happy questions [*M* = 5.13, SD = 1.48, *t*(1105) = 1.25, *p* = 0.210] and the happy writing [*M* = 5.15, SD = 1.55, *t*(1105) = 1.40, *p* = 0.161], generated greater happy/good feelings than the control (*M* = 4.97, SD = 1.56). Thus, although some of the treatments worked as intended, the effects are inconsistent (and may be due to the measurement of emotions, which we address below).

**Fig. 1:**
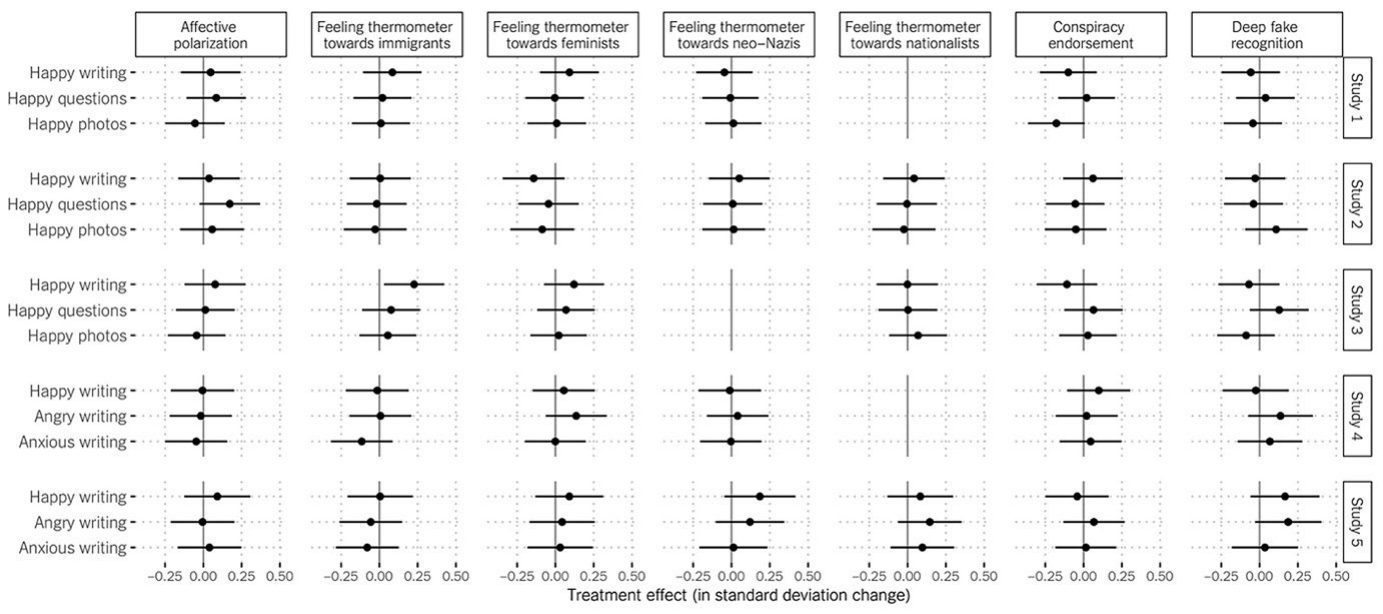
The effects of treatments on affective polarization, feeling thermometers toward divisive social groups, conspiracy endorsement, and deep fake recognition

Regression models predicted each of our dependent variables (i.e., aggregate affective polarization, feelings toward the tested social groups, conspiracy endorsement, and deep fake recognition) from the happiness treatments (with the control group as the reference category, and age as a covariate in Poland). To account for multiple tests, false discovery rate (FDR; Benjamini and Hochberg 1995; Yekutieli and Benjamini 1999) adjustments were applied. Specifically, we sorted p-values from lowest to highest and then multiplied each p-value by the total number of tests *m* and divided by its rank order *i*. This approach was applied to all the studies. We plot our results (i.e., the effects of treatments in standard deviation change with 95% confidence intervals) in Fig. 1 and present the descriptives and regression tables for all the studies in Appendix G and H, respectively.

Study 1 on the American sample finds absolutely no effects of incidental happiness on affective polarization. A regression model predicting the aggregate affective polarization index showed that all our happiness treatments are indistinguishable from the control (*ps* > 0.05, FDR adjustment; see Fig. 1). Feelings toward the various divisive groups were also not affected: be it immigrants, feminists, or neo-Nazis (*ps* > 0.05, FDR adjustment). Similarly, Study 2 in Poland finds that the happiness treatments and the control did not differ in their effects on the aggregate index and on feeling thermometers toward the tested social groups (*ps* > 0.05, FDR adjustment). In short, incidental happiness did not attenuate affective polarization and hostility toward divisive social groups, contrary to *H1*.

As seen in Fig. 1, parallel null effects emerged with regard to conspiracy endorsement and believing a deep fake video was real. In both countries, incidental happiness had no effects neither on conspiracy endorsements nor on deep fake recognition (*ps* > 0.05, FDR adjustment). Therefore, *H2a* and *H2b* are also rejected.

We also tested for pre-registered heterogeneous treatment effects by political identity strength and political interest, together with exploratory analysis by political affiliation (Republicans vs. Democrats in the US; government supporters vs. opponents in PL) and ideology. Although in Poland, participants in the happy questions treatment condition with greater political interest had more positive attitudes toward immigrants than those who were less interested in politics (*b* = 0.204, *p* = 0.048, FDR adjustment) and government supporters in the happy photos treatment condition were less likely to believe that the deep fake was true than government opponents (*b* = − 0.172, *p* = 0.016, FDR adjustment), we did not identify any consistent interaction effects in either study (see Appendix H for regression tables). Also, although not pre-registered, we speculated that the effects could depend on the extent to which the manipulations were effective, such that the most pronounced effects could emerge among those who felt greater happiness following the treatments. Also here, we find an insignificant pattern of results except that in the US, those who felt greater happiness after receiving the happy writing treatment were less likely to endorse conspiracy theories compared to those who were less happy (*b* = − 0.259, *p* = 0.046, FDR adjustment; see Appendix H). Apart from this sole exception, happiness effects on affective polarization, conspiracy endorsement, and deep fake do not depend on the intensity with which the induced emotion was felt.

As aforementioned, across all five studies, we re-estimated all the models testing the effects of incidental happiness on affective polarization toward the individual political outgroups (e.g., outgroup elites and outgroup supporters) with the individual indicators of affective polarization (i.e., feeling thermometers, trait ratings, social distance, and outgroup trust) as outcomes. For these indicators, we also tested heterogeneous treatment effects by political identity strength, political interest, political affiliation, ideology, and the effectiveness of the treatments. The largely insignificant results are presented in Appendix H.

### Study 1 and 2: Discussion

Thus far, we find no evidence that incidental happiness influences the tested outcomes. However, rather than rushing to the conclusion that incidental happiness does not shift individual political judgements and cognitions, we suspect that the almost null effects could be due to the fact that the happiness treatments did not consistently increase happiness and/or that the manipulation check items eliminated the treatment effects. We find no effects of our treatments on one manipulation check battery and only weak and inconsistent effects on the other (i.e., in both countries, only the *happy photos* treatment made people feel happier than the control, yet it did not necessarily exert the most pronounced effects). This could be because the control group did not engage in any task while those receiving the happy treatments had to answer additional questions or complete a writing task. In addition to the difference in the workload, the 10-item manipulation check battery that contained only positive emotions might have primed the control participants with happiness, minimizing the difference between the control and the happiness conditions.

### Study 3: Design and Measures

We address these limitations in Study 3, fielded in the Netherlands (and in the subsequent studies reported later).^9^ To check the effectiveness of the treatments, we used the self-assessment manikin (SAM), a visual scale designed as an alternative to the problematic verbal self-reports of emotions (Lang 1985). Also, in Study 3, each happiness condition was accompanied by its corresponding control condition, as detailed below. The core of the design remained unchanged. The questionnaires and the treatments were in Dutch.

Study 3 was conducted in June 2019, on an adult Dutch sample recruited by Dynata. Quotas were set for age, gender, education, and ideology. Ideological independents (core political identity in the NL, Andeweg and Irwin 2014; Silva 2018) were automatically filtered out, as it is not possible to determine the political outgroup for those participants. In total, 1457 participants completed the study. As before, those who failed the attention check (*n* = 162) and those who completed the study in under 4 min 12 s (*n* = 67) were excluded from the analyses. The final sample includes 1254 participants.

Similarly to the prior design, the pretest measured political interest, political identity strength, and participants’ current emotional state, in addition to standard socio-demographics and political variables (e.g., political ideology on the 11-point ideology scale, and respondents’ most and least liked political party, selected from a list of thirteen political parties in the Netherlands). Following the pretest, participants were randomly assigned to one of the six treatment groups: *happy writing* (*n* = 188), *happy questions* (*n* = 207), *happy photos* (*n* = 218), *control writing* (*n* = 215), *control questions* (*n* = 211), and *control photos* (*n* = 215; randomization was successful, see Appendix E for details). In the *control writing* treatment, participants were asked to describe what they were seeing in their surroundings; in the *control questions* treatment, they answered six filler questions (e.g., “What did you have for breakfast?”); in the *control photos* treatment condition, five photos of geometric figures were presented (e.g., a photo of a circle; see Appendix I for details). The *happy* treatments remained unchanged. After exposure, the posttest included the visual self-assessment manikin (SAM; Lang 1985) as a manipulation check (see Appendix I).

To assess affective polarization, we again used feeling thermometers toward the most liked and least liked party and their supporters, and toward those from the political left and right. As before, we used participants’ pre-test partisanship and ideology to determine their partisan and ideological outgroups. The remaining indicators of affective polarization (i.e., social distance, trait evaluations, and outgroup trust) were measured as in Study 1 and 2, adapted to ask about supporters of the opposing ideology (e.g., the political left for self-identified right-leaning participants). To create the index of affective polarization, we rescaled all the outgroup items between 0 and 1 and averaged (*M* = 0.38, SD = 0.12, *α* = 0.75). Additionally, participants rated feelings toward immigrants, feminists, and nationalists, which were used as individual outcome variables. As before, conspiracy endorsement and deep fake recognition were also measured (the deep fake video was shown with Dutch subtitles). These variables were also rescaled.

### Study 3: Results

Participants in the *happy photos* treatment (*M* = 6.97, SD = 1.76) were happier than those in the control photos [*M* = 6.45, SD = 1.65; *t*(1248) = 3.37, *p* < 0.001] and those in the *happy writing* treatment (*M* = 6.87, SD = 1.59) were happier than those in the control writing [*M* = 6.40, SD = 1.62; *t*(1248) = 2.88, *p* = 0.004]. Even though the means were in the predicted direction, there was no difference between *happy questions* treatment (*M* = 6.61, SD = 1.59) and the control questions condition [*M* = 6.46, SD = 1.43; *t*(1248) = 0.95, *p* = 0.345]. Because the three control conditions did not differ from each other in their effects on happiness levels [*F*(2, 638) = 0.07, *p* = 0.931], they were combined into one general control condition (*M* = 6.44, SD = 1.57). Participants in the *happy photos* [*t*(1250) = 4.21, *p* < 0.001] and the *happy writing* [*t*(1250) = 3.22, *p* = 0.001] treatments were happier than those in the general control condition but the *happy questions* treatment [*t*(1250) = 1.34, *p* = 0.182] again did not differ from the general control condition, suggesting that this was the weakest treatment.

Regression models with the general control condition entered as a reference did not find significant differences between the happy and the control conditions in their effects on the aggregate index of affective polarization (*ps* > 0.05, FDR adjustment; see Fig. 1). In terms of feelings toward the tested social groups, the results were mainly insignificant (*ps* > 0.05, FDR adjustment) except for one case: participants in the happy writing condition felt 0.049 warmer on the normalized scale toward immigrants than those in the control (*p* = 0.030, FDR adjustment). Because the happy questions and happy photos treatments exerted no effects whatsoever and the happy writing treatment had no effects on other outcomes, we caution against putting too much leverage on this substantively small effect. Thus, *H1* is rejected also for this study. In line with the results presented above, incidental happiness had no effects on neither conspiracy beliefs nor deep fake recognition (*ps* > 0.05, FDR adjustment), again leading us to reject *H2a* and *H2b*.

We then tested for the pre-registered heterogeneous effects by political identity strength (based on ideological identification with the left or the right) and political interest and also explored whether the effects of manipulations depend on ideology and the posttest happiness. Participants in the happy photos treatment condition with stronger political identity had more positive attitudes toward their political opponents than those with weaker political identity (*b* = 0.145, *p* = 0.026, FDR adjustment), and also the political right in the happy writing treatment condition had warmer feelings toward the opposition than the left (*b* = 0.062, *p* = 0.011, FDR adjustment), suggesting that happiness can have potential to be effective among those with strong political priors and among different political groups. Also, those for whom the happy photos condition generated the greatest levels of good/happy feelings were more likely to endorse conspiracies than those for whom the treatment was less effective (*b* = 0.305, *p* < 0.001, FDR adjustment), an exploratory finding partly consistent with the idea that experienced happiness may minimize more critical and effortful processing. As above, because these moderating effects were rather limited and not consistent, we treat them as merely suggestive.

### Study 3: Discussion

Although we improved the design, and participants in the *happy photos* and *happy writing* treatments were indeed happier than those in the corresponding control conditions (as assessed using the visual measurement of emotional states), Study 3 again demonstrated that incidental happiness has no effects whatsoever on affective polarization. Almost consistent null effects also emerged for hostility toward the individual political outgroups (as shown in Appendix H) and the various divisive social groups. Its effects on conspiracy endorsements and deep fake recognition were also insignificant. We speculated that although the visual SAM is a more unobtrusive way to assess emotions, measuring emotions immediately after the happiness induction might result in the happiness “spilling over” to the manipulation check items (as it should) rather than to the core outcomes presented later, minimizing its effects. Also, it could be argued that one should start by testing what emotions *exacerbate* affective polarization, and only later examine how these exacerbating emotions can be minimized toward attenuating affective polarization. Thus, in addition to incidental happiness, we induced two negative incidental emotions—anger and anxiety—and tested their effects on affective polarization.

### Study 4 and 5: Design and Measures

Theoretically, Study 4 and 5 (which were not pre-registered and are largely exploratory) examined the effects of incidental happiness, anger, and anxiety on affective polarization and conspiracy endorsement and believing that the deep fake is real. Anger and anxiety should exacerbate affective polarization because—when asked to evaluate the political outgroup—angry and anxious citizens will recall more unpleasant thoughts and misattribute the current negative feelings to the outgroup. Our third non-pre-registered prediction, therefore, is that *participants induced to feel angry and anxious will exhibit higher affective polarization than participants in the control condition (H3)*. When it comes to conspiracy endorsement and believing that a deep fake video is real, anger should enhance these outcomes because angry people are certain about what happened (Lerner and Keltner 2000) and do not process information analytically (Bodenhausen et al. 1994). In contrast, anxiety is associated with uncertainty (Lerner and Keltner 2000), thus promoting more systematic information processing (Meijnders et al. 2001; Tiedens and Linton 2001). Therefore, we speculated that *participants in the angry condition will be more likely to endorse conspiracies and less likely to recognize the deep fake video as fake than those in the control* (*H4)*, whereas those in the *anxious condition will be less likely to endorse conspiracies and more likely to recognize the deep fake video as fake than those in the control* (*H5)*.

We conducted Study 4 (the US) and 5 (Poland) using participants recruited via Dynata in August 2019 (the US) and Panel Ariadna in September 2019 (Poland). As before, those who had no clear political leanings (partisan independents close to neither party in the US and those who neither supported nor opposed the current government in Poland) were automatically excluded. A total of 1269 American partisans completed Study 4 and 1190 Polish adults completed Study 5. Those who failed the attention check (in Study 4 they were excluded automatically; *n* = 111, Study 5) and completed the study in under 48% of the median time (4 min 44 s, *n* = 104, Study 4; 4 min 25 s, *n* = 35, Study 5) were excluded. The final samples include 1165 participants (Study 4) and 1055 participants (Study 5).

After the pretest, which included the same measures as the previous studies except for the current emotional state, participants were randomly assigned to *happy writing* (US: *n* = 280, Poland: *n* = 232), *angry writing* (US: *n* = 305, Poland: *n* = 265), *anxious writing* (US: *n* = 308, Poland: *n* = 266), or the *control writing* condition (US: *n* = 272, Poland: *n* = 292); randomization was successful, see Appendix E. Writing tasks were used because they allow us to induce anger and anxiety independently (in contrast, threatening photos may stimulate fear, anger, and/or mixed emotions; Valentino et al. 2011). In addition, writing treatments enable us to measure emotional states unobtrusively: instead of asking participants to report their emotional states, we coded the intensity of emotions expressed in the writing task, as detailed below (see Valentino et al. 2011). The rest of the design was unchanged from Study 1 and 2. Following the treatments, participants completed a posttest, with the same country-specific measures used in Study 1 and Study 2. The aggregate index was created following the same procedure as in Study 1 and 2 (*M* = 0.41, SD = 0.19, α = 0.86, Study 4; *M* = 0.31, SD = 0.16, α = 0.82, Study 5) and the other outcome variables were rescaled.

### Study 4 and 5: Results

To assess whether the treatments elicited the intended emotions, research assistants (two native English speakers in the US and two native Polish speakers in PL) blind to the conditions and the hypotheses coded the open-ended responses for the intensity of specific emotions (happiness, anger, anxiety), from no emotion, to somewhat intense emotion, to very intense emotion (coded as 0, 1, and 2 respectively; in Study 4, Cohen’s κ = 0.73, 0.75, and 0.71 for happiness, anxiety, and anger; in Study 5, Cohen’s κ = 0.83, 0.61, and 0.60, respectively). In the US, the happiness writing treatment had the highest value on happy scores [*M* = 0.95, SD = 0.70; *F*(3, 1161) = 437.75, *p* < 0.001], anger treatment had the highest value on angry scores [*M* = 0.91, SD = 0.74; *F*(3, 1161) = 344.69, *p* < 0.001], and anxiety treatment had the highest value on anxiety scores [*M* = 0.99, SD = 0.75; *F*(3, 1161) = 396.67, *p* < 0.001]. These scores were significantly different from the other conditions (all *p*s < 0.001). In Poland, the happy treatment had the highest value on happy scores [*M* = 1.00, SD = 0.81; *F*(3, 1051) = 379.43, *p* < 0.001], the anger treatment had the highest value on angry scores [*M* = 0.72, SD = 0.75; *F*(3, 1051) = 149.78, *p* < 0.001], and the anxiety treatment had the highest value on anxious scores [*M* = 0.58, SD = 0.75: *F*(3, 1051) = 126.63, *p* < 0.001]. These scores were significantly different from other conditions (all *p*s < 0.001) although all these differences were not substantial.^10^

Again, in the US, we find no significant effects of happiness, anger, and anxiety on the index of affective polarization and hostility toward the social groups tested (*ps* > 0.05, FDR adjustment; see Fig. 1). In Poland, the same pattern of insignificant results emerged: participants in the three incidental emotions conditions, i.e., happiness, anger, and anxiety, did not differ from those in the control with regard to the affective polarization measure and hostility toward divisive social groups (*ps* > 0.05, FDR adjustment). Thus, *H1* and *H3* are not supported. Consistent with the results presented above, in both studies, no incidental emotion had any influence on conspiracy beliefs or believing that the deep fake video was real (*ps* > 0.05, FDR adjustment), rejecting *H2, H4,* and *H5*.^11^

We again tested for heterogeneous treatment effects by political identity strength, political interest, political identity, ideology, and the intensity of the posttest emotions. In the US, participants who had strong partisan identity and were in the angry writing treatment condition (*b* = − 0.338, *p* = 0.035, FDR adjustment) or anxious writing treatment condition reported greater hostility toward feminists compared to those with weaker partisan identity (*b* = − 0.445, *p* < 0.001, FDR adjustment). Apart from these exceptions, no heterogeneous effects were found. In sum, the interaction effects were largely insignificant and inconsistent.

### Study 4 and 5: Discussion

Study 4 and 5 used writing tasks to elicit three distinct emotions and checked the effectiveness of the treatments unobtrusively. As determined based on independent coding done by trained coders in both countries, happiness, anger, and anxiety were successfully induced. These emotions, although different in valence and the behavioral reaction they should theoretically elicit (see Nabi 1999; Lazarus 1991), had almost null effects on all the outcomes, validating and extending the findings from the previous three studies.

## General Discussion

We attended to potential effects of positive emotions in politics and extended the research on irrelevant non-political factors influencing political outcomes. Drawing on the classic work from social psychology on misattribution of affect and the spillover or carryover effect (De Hooge et al. 2014; Lerner et al. 2004; Payne et al. 2010; Schwarz and Clore 1983), we considered the potential of incidental happiness to mitigate affective polarization. In various largely non-political contexts studied to date, making people feel happy (i.e., eliciting incidental happiness that is unrelated to an evaluated object) has led them to make more positive evaluations of objects, ranging from their lives to co-workers and acquaintances (e.g., Dunn and Schweitzer 2005; Schwarz and Clore 1983). Our theoretical hope was that incidental happiness would work similarly in the political context, leading partisans to evaluate their political outgroups more positively. We also speculated, however, that those happy partisans would default to the less effortful, more peripheral, ways of processing political information, and thus be more likely to endorse misinformed beliefs.

Using five original experiments in three distinct countries, asking about attitudes toward various groups, and using distinct approaches to eliciting emotions, we find no evidence that incidental happiness, or anger and anxiety, have any effects on affective polarization toward country-specific political outgroups or hostility toward the divisive social groups generally disliked by the political left or right. We also fail to detect any effects of happiness and of the two negative emotions on individuals’ endorsement of various conspiracy theories (such as the truth about the harmful effects of vaccines is being deliberately hidden from the public or the idea of man-made global warming is a hoax that was invented to deceive people) and people’s belief that a deep fake video featuring Donald Trump discussing climate change is actually real.

These largely null findings are not due to design decisions (which were gradually adapted and refined), the ineffectiveness of the emotional inductions (which had inconsistent effects on the actual emotions in the first set of studies only), lack of statistical power, or the tested groups (again, we assessed affective polarization toward political outgroups as based on partisanship and ideology, and additionally in Poland as based on one’s support and opposition toward the government, asking participants about their feelings toward political elites as well as ordinary citizens; and we also measured animosity toward various social groups).

These findings have several important implications. First, they underscore the growing difficulties of changing people’s attitudes toward political outgroups (either minimizing affective polarization with happiness or exacerbating it with anger or anxiety) in contemporary US and also in two distinct democracies, the Netherlands and Poland. As affective polarization in the US and elsewhere has grown (e.g., Boxell et al. 2020; Gidron et al. 2020), recent efforts to attenuate interparty hostility have proven largely unsuccessful (e.g., Levendusky 2018b; Wojcieszak and Warner 2020). Because many other salient identities have been increasingly coalescing around partisanship in the US (such as ethnicity or religion), leading some to term it a “super identity” (Mason 2018), it may be ever more difficult to shift political attitudes and minimize outparty hostility (although one would think that the negative emotions elicited in study 4 and 5 should have the power to exacerbate it). Our treatments, although theoretically grounded and effective in non-political contexts, may have been too subtle and too removed from the outcomes tested. Had we tested happiness as related to the political outgroup (e.g., asking our US participants to write about how the Democratic/Republican Party made them feel happy), the treatments would have likely influenced the outcomes. Because our interest was in happiness that is *conceptually unrelated* and *irrelevant* to the political outcome (and easily evoked by social media platforms or other organizations interested in a more cohesive polity), asking people about the political outgroup directly was not considered realistic and versatile.

Our second major interest was in whether incidental happiness may produce unintended side effects. Also with regard to conspiracy endorsement and believing a deep fake video, we find no effects of happiness (or of anger and anxiety). This may be because various misinformed beliefs, and especially those related to vaccinations, immigration, climate change, and other divisive issues we tested, are closely linked to individual political identity (e.g., Pew 2019b) and hard to influence with rather subtle and thematically distinct treatments.

It is possible that incidental emotions vanish immediately when citizens make political judgements or are exposed to politically-charged conspiracy theories and deep fake videos because political identity and opinions are more powerful and more easily activated than non-political ones. Although this contradicts previous finding that incidental emotions can influence policy recommendations related to welfare assistance (Small and Lerner 2008) and trust in government (Webster 2018), the welfare policies are more complex and the concept of governmental trust may be more abstract than visceral affective evaluations of one’s political opponents. We speculate that when people are asked to evaluate their political outgroup or make judgements about controversial political issues, their political identity is activated together with the associated negative feelings toward the outgroup. These feelings quickly spread through one’s associative network (see Lodge and Taber 2005), eliminate incidental emotions, and drive one’s judgements and information processing.

More broadly, our findings suggest that incidental emotions, which have been found to work in various contexts, may not apply to political hostility. This reveals important boundary conditions of the classic theory on misattribution of affect. In general, various psychological frameworks, founded based on results from American undergraduate students with regard to non-ego-involving issues, may not generalize to the real-world of partisan politics and identity-rooted attitudes, cognitions, and behaviors that are at the core of contemporary social divides. More work, akin to our project and other similar efforts, is needed to test the applicability of social-psychological theorizing to the consequential domain of political conflict.

## Supplementary Information

Below is the link to the electronic supplementary material.

Supplementary file 1 (DOCX 3060 kb)

## Footnotes

1 The online processing model (Lodge et al. 2011) similarly suggests that spontaneous affective responses produced at the moment of processing information about a policy or a political candidate subsequently influence people's evaluation of the candidate, regardless of the content of the information. This project tests affective reactions generated by information/inductions that are non-political and irrelevant to the object of the evaluation.

2 Power analysis was conducted after data collection. Past work on mitigating affective polarization mostly finds very small effect sizes (e.g., *f*^2^ of most models is smaller or equal to 0.01 in Ahler and Sood 2018; Levendusky 2018a), if at all (e.g., Levendusky 2018b; Wojcieszak and Warner 2020). Additionally, Levendusky (2018a) found no heterogeneous treatment effects for either strong or sorted partisans. With regard to misinformed beliefs, Forgas and East (2008), who tested the effects of happy, neutral, and sad moods on skepticism and the detection of deception did not report effect sizes. Based on these studies, we adopted a conservative approach, with the goal of obtaining .80 power to detect a small effect size (*f*^2^) of .01 at the standard .05 alpha error probability for both main (*H1* and *H2*) and heterogeneous effects. The required *n* was approximately 1100. As such, all the studies reported in this manuscript have sufficient power to detect the tested effects except for Study 5, which has slightly fewer (*n* = 1055) participants than required.

3 Some questions might evoke negative emotions in some participants (e.g., one’s favorite song could induce nostalgia). However, it is unlikely that all of these six questions would have evoked negative emotions, especially that one question directly asked what made participants most happy.

4 Given the fragmented multi-party system in Poland, we first asked participants to select their most preferred party and later the least preferred party (from a list of 10 parties). The feeling thermometer questions asked about both.

5 In both countries, the true ideological moderates’ (5 on the 0—10 scale) feelings toward out-ideologues were treated as missing values and excluded in analysis.

6 To speak to the literature that defines affective polarization as the difference between the in- and out-group scores, in all five studies, we re-estimated exploratory models predicting the effects of our treatments on the difference between in- and outgroup feeling thermometers (see Appendix H for the results). For these subtractive indicators, we tested heterogeneous treatment effects by political identity strength, political interest, political affiliation, ideology, and effectiveness of the treatments, adjusting for multiple comparisons (see Appendix H for the results).

7 https://www.politico.eu/article/spa-donald-trump-belgium-paris-climate-agreement-belgian-socialist-party-circulates-deep-fake-trump-video/

8 Because the pretest tiredness was not equally distributed across conditions (*F*(3, 1244) = 3.26, *p* = .021), these models include it as a covariate.

9 Although the sociopolitical context is distinct from those of Study 1 and 2, our theoretical expectations should hold. Also, the subsequent studies (Study 4 and 5) were conducted in the US and PL.

10 Due to a programming error in Poland, those who were assigned to the control writing condition did not get the writing task. We assign 0 to them for all three emotions.

11 Parallel models with the happy writing condition entered as the reference group produced similar null effects.
