# Supplementary material for: The (Null) Effects of Happiness on Affective Polarization, Conspiracy Endorsement, and Deep Fake Recognition: Evidence from Five Survey Experiments in Three Countries
Source: Polit Behav. 2021 Mar 18;43(3):1265–87. doi: 10.1007/s11109-021-09701-1 (PMC8550506; doi:10.1007/s11109-021-09701-1)
Supplement: Supplementary file 1 — (DOCX 3060 kb) [file 11109_2021_9701_MOESM1_ESM.docx]

**Appendix A: Deviations from pre-registration**

For Study 1, we indicated in the preregistration that we would also measure feelings toward the Democratic Party candidates and elected officials and the Republican Party candidates and elected officials, but we deleted these two items from the final questionnaire to make it shorter.

For Study 2 (PL), the size of municipality in PL was added to quotas after pre-registration.

For study 3, we used the self-assessment manikin (SAM) as manipulation check and each happiness condition was accompanied by its corresponding control condition. These changes were implemented after pre-registration. Moreover, in the pre-registration, we indicated that we would measure feelings toward the most liked party candidates and elected officials, and the least liked party candidates and elected officials, but we deleted these two items from the final questionnaire to make it shorter. We also replaced environmentalists with nationalists for consistency with Study 2.

For all three studies, even though the pre-registration materials specified that the political *ingroups* will also be used as the outcome variables, for parsimony, we only present the results for the outgroups. The happiness effects on feelings toward the ingroup were uniformly insignificant. For trait ratings, the preregistration included an item “American,” which - however - was removed from the questionnaire to keep the traits consistent across the countries. The preregistration also specified that we would estimate ANOVA models with planned contrasts to test all the hypotheses across all studies, but ultimately, we used regression models because it is easier to interpret the treatment effects and interaction effects. Besides, we proposed to test the heterogeneous effects of age, gender, education, and ethnicity, but for parsimony, we did not report the results. No consistent patterns emerged. Moreover, we predicted that happiness would affect intentions to share political content, to unfriend/block/unfollow someone who has different opinions, and to follow the opposition. Again, for parsimony, we did not present the results. The happiness effects on these variables were uniformly insignificant. Additionally, for parsimony and following recent work (e.g., Druckman et al. 2020), we present the results for the aggregate index of affective polarization (the pre-registered analyses of each individual affective polarization indicator toward various outgroups are presented in Appendix H). We also applied false discovery rate adjustments after pre-registration because a great number of tests were run.

**Appendix B: Results of the pilot study**

The pilot, conducted on 511 undergraduate students, found that the happy writing treatment (*M* = 4.50, *SE* = .13) made people happier than the happy photo treatment (we tested the effectiveness of two treatments: happy photo condition 1: *M* = 3.99, *SE* = .12, *p* = .004; happy photo condition 2: *M* = 4.07, *SE* = .12, *p* = .013) and the happy questions treatment (*M* = 4.06, *SE* = .12, *p* = .012). In the main studies, we used five photos in the happy photos condition, including the two photos from the pilot (although in the preregistration we indicated that all five photos would be piloted).

**Appendix C: Demographics**

**Table C.1.1 Demographic description of the samples**

|  |  | Study 1 | Study 2 | Study 3 | Study 4 | Study 5 |
| --- | --- | --- | --- | --- | --- | --- |
| Age | Mean (SD) | 48.47 (17.16) | 45.59 (15.46) | 50.85 (16.29) | 49.73 (16.71) | 44.87 (15.41) |
| Gender | Male | 589 (47.20%) | 481 (43.37%) | 606 (48.33%) | 570 (48.93%) | 497 (47.11%) |
|  | Female | 659 (52.80%) | 628 (56.63%) | 648 (51.67%) | 595 (51.07%) | 558 (52.89%) |
| Education | Mean (SD) | 4.78 (1.49) | 4.19 (1.50) | 5.97 (2.00) | 4.67 (1.51) | 4.14 (1.52) |
| Political  predisposition |  | 663 (53.13%) (Democrat) | 676 (60.96%)  (Gov. opponent) | 588 (46.89%)  (Left) | 580 (49.79%)  (Democrat) | 626 (59.34%)  (Gov. opponent) |
|  |  | 585 (46.88%) (Republican) | 433 (39.04%)  (Gov. supporter) | 666 (53.11%)  (Right) | 585 (50.21%)  (Republican) | 429 (40.66%)  (Gov. supporter) |

**Appendix D: Question wording and descriptive statistics**

**Table D.1.1** Variable description

|  | **Study 1 (USA)** | | | **Study 2 (Poland)** | | | **Study 3 (Netherland)** | | | **Study 4 (USA)** | | | **Study 5 (Poland)** | | |
| --- | --- | --- | --- | --- | --- | --- | --- | --- | --- | --- | --- | --- | --- | --- | --- |
|  | *α* | *M* | *SD* | *α* | *M* | *SD* | *α* | *M* | *SD* | *α* | *M* | *SD* | *α* | *M* | *SD* |
| **Pretest Happiness** | .80# | 5.61 | 1.38 | .64# | 5.04 | 1.47 | .76# | 5.25 | 1.29 | - | - | - | - | - | - |
| Please indicate again how you feel at this moment.  – Bad : Good |  |  |  |  |  |  |  |  |  |  |  |  |  |  |  |
| – Sad : Happy |  |  |  |  |  |  |  |  |  |  |  |  |  |  |  |
| **Pretest Tiredness** | .61# | 5.08 | 1.61 | .63# | 4.28 | 1.58 | .61# | 4.82 | 1.45 | - | - | - | - | - | - |
| – Tired:Rested |  |  |  |  |  |  |  |  |  |  |  |  |  |  |  |
| – Tense:Relaxed |  |  |  |  |  |  |  |  |  |  |  |  |  |  |  |
| **Political Identity Strength** | .81 | 5.07 | 1.21 | .80 | 4.78 | 1.37 | .83 | 4.21 | 1.12 | .80 | 5.25 | 1.18 | .81 | 4.81 | 1.39 |
| – I often think of myself as a […] |  |  |  |  |  |  |  |  |  |  |  |  |  |  |  |
| – I consider myself a typical […] |  |  |  |  |  |  |  |  |  |  |  |  |  |  |  |
| – I'm proud that I'm a […] |  |  |  |  |  |  |  |  |  |  |  |  |  |  |  |
| – If someone said something bad about […] I feel as if they said something bad about me |  |  |  |  |  |  |  |  |  |  |  |  |  |  |  |
| **Political interest**  – How interested are you in politics? | - | 4.74 | 1.72 | - | 4.56 | 1.53 | - | 4.42 | 1.50 | - | 4.87 | 1.73 | - | 4.54 | 1.62 |
| **Ideology**  – Where would you place yourself on this scale, where 0 means left and 10 means right? | - | 5.67 | 2.95 | - | 5.29 | 2.89 | - | 5.05 | 2.66 | - | 6.03 | 2.93 | - | 5.29 | 3.03 |
| **Posttest Happiness (Semantic)** | .82# | 5.74 | 1.34 | .67# | 5.12 | 1.51 | - | - | - | - | - | - | - | - | - |
| Please indicate again how you feel at this moment.  – Bad : Good |  |  |  |  |  |  |  |  |  |  |  |  |  |  |  |
| – Sad : Happy |  |  |  |  |  |  |  |  |  |  |  |  |  |  |  |
| **Posttest Tiredness** | .65# | 5.29 | 1.56 | .63# | 4.49 | 1.58 | - | - | - | - | - | - | - | - | - |
| – Tired : Rested |  |  |  |  |  |  |  |  |  |  |  |  |  |  |  |
| – Tense : Relaxed |  |  |  |  |  |  |  |  |  |  |  |  |  |  |  |
| **Posttest Happiness (PANAS)** | .94 | 4.85 | 1.19 | .90 | 4.40 | 0.97 | - | - | - | - | - | - | - | - | - |
| Please also indicate how much you feel the following emotions right now. - Enthusiastic |  |  |  |  |  |  |  |  |  |  |  |  |  |  |  |
| – Interested |  |  |  |  |  |  |  |  |  |  |  |  |  |  |  |
| – Determined |  |  |  |  |  |  |  |  |  |  |  |  |  |  |  |
| – Excited |  |  |  |  |  |  |  |  |  |  |  |  |  |  |  |
| – Inspired |  |  |  |  |  |  |  |  |  |  |  |  |  |  |  |
| – Alert |  |  |  |  |  |  |  |  |  |  |  |  |  |  |  |
| – Active |  |  |  |  |  |  |  |  |  |  |  |  |  |  |  |
| – Strong |  |  |  |  |  |  |  |  |  |  |  |  |  |  |  |
| – Proud |  |  |  |  |  |  |  |  |  |  |  |  |  |  |  |
| – Attentive |  |  |  |  |  |  |  |  |  |  |  |  |  |  |  |
| **Posttest Happiness (SAM)** | - | - | - | - | - | - | - | 6.62 | 1.62 | - | - | - | - | - | - |
| **Feeling thermometers**  We’d like you to rate several different groups of people using a “feeling thermometer”. The higher the number, the warmer or more favorable you feel toward the group; the lower the number, the colder or less favorable. Please rate how you feel about the following groups:Please rate how you feel about the following groups: |  |  |  |  |  |  |  |  |  |  |  |  |  |  |  |
| – The outparty/least favorite party | - | 22.47 | 24.11 | - | 15.67 | 22.73 | - | 14.84 | 19.51 | - | 26.13 | 26.04 | - | 16.22 | 22.13 |
| – Outparty/ least favorite party supporters | - | 26.90 | 24.04 | - | 18.12 | 23.53 | - | 18.73 | 20.22 | - | 30.20 | 25.58 | - | 18.76 | 22.74 |
| – Out-ideologues | - | 32.09 | 27.75 | - | 31.35 | 23.22 | - | 37.54 | 18.93 | - | 36.00 | 28.27 | - | 32.25 | 22.86 |
| – Government supporters/opponents | - | - | - | - | 22.58 | 23.41 | - | - | - | - | - | - | - | 24.92 | 23.20 |
| – Immigrants | - | 59.04 | 27.79 | - | 39.13 | 26.58 | - | 54.44 | 21.26 | - | 59.92 | 27.05 | - | 41.79 | 26.55 |
| – Feminists | - | 54.61 | 30.69 | - | 46.08 | 29.58 | - | 54.15 | 22.58 | - | 51.19 | 28.59 | - | 47.64 | 29.03 |
| – Neo-Nazis | - | 10.57 | 21.35 | - | 7.56 | 15.20 | - | - | - | - | 12.76 | 23.26 | - | 11.47 | 19.92 |
| – Nationalists | - | - | - | - | 27.90 | 27.30 | - | 43.97 | 22.40 | - | - | - | - | 28.73 | 27.66 |
| **Social distance** | .93 | 4.53 | 1.53 | .93 | 4.22 | 1.53 | .93 | 4.54 | 1.19 | .93 | 4.77 | 1.49 | .92 | 4.19 | 1.46 |
| How comfortable or uncomfortable would you be to interact with […] as:  - Someone I have to work closely with at my job |  |  |  |  |  |  |  |  |  |  |  |  |  |  |  |
| - A close relative by marriage |  |  |  |  |  |  |  |  |  |  |  |  |  |  |  |
| - A neighbor on the same street |  |  |  |  |  |  |  |  |  |  |  |  |  |  |  |
| **Traits ratings** | .81 | 3.45 | 1.13 | .84 | 3.45 | 1.14 | .77 | 3.93 | 0.83 | .84 | 3.55 | 1.23 | .85 | 3.41 | 1.15 |
| Please indicate the extent to which you agree or disagree that […] are:  – Intelligent |  |  |  |  |  |  |  |  |  |  |  |  |  |  |  |
| – Open-minded |  |  |  |  |  |  |  |  |  |  |  |  |  |  |  |
| – Honest |  |  |  |  |  |  |  |  |  |  |  |  |  |  |  |
| – Hypocritical (R) |  |  |  |  |  |  |  |  |  |  |  |  |  |  |  |
| – Selfish (R) |  |  |  |  |  |  |  |  |  |  |  |  |  |  |  |
| – Mean (R) |  |  |  |  |  |  |  |  |  |  |  |  |  |  |  |
| **Outgroup trust** | .95 | 3.47 | 1.51 | .93 | 3.07 | 1.35 | .92 | 4.01 | 1.14 | .95 | 3.63 | 1.56 | .93 | 3.08 | 1.37 |
| – Most […] are basically honest |  |  |  |  |  |  |  |  |  |  |  |  |  |  |  |
| – Most […] are trustworthy |  |  |  |  |  |  |  |  |  |  |  |  |  |  |  |
| – Most […] are basically good and kind |  |  |  |  |  |  |  |  |  |  |  |  |  |  |  |
| – Most […] will respond in kind when they are trusted by others |  |  |  |  |  |  |  |  |  |  |  |  |  |  |  |
| **Conspiracy beliefs** | .84 | 2.39 | .97 | .77 | 2.63 | .79 | .83 | 2.41 | .90 | .83 | 2.53 | .95 | .75 | 2.66 | .77 |
| – The government is deliberately hiding the truth about how many immigrants really live in the country |  |  |  |  |  |  |  |  |  |  |  |  |  |  |  |
| – Immigration to this country is part of a bigger plan to make Muslims a majority of the country’s population |  |  |  |  |  |  |  |  |  |  |  |  |  |  |  |
| – The truth about the harmful effects of vaccines is being deliberately hidden from the public |  |  |  |  |  |  |  |  |  |  |  |  |  |  |  |
| – The idea of man-made global warming is a hoax that was invented to deceive people |  |  |  |  |  |  |  |  |  |  |  |  |  |  |  |
| – Regardless of who is officially in charge of governments and other organizations, there is a single group of people who secretly control events and rule the world together |  |  |  |  |  |  |  |  |  |  |  |  |  |  |  |
| – Vaccines are dangerous |  |  |  |  |  |  |  |  |  |  |  |  |  |  |  |
| **Fake video perceptions**  – This video was – fake : real | - | 1.59 | .96 | - | 2.20 | 1.32 | - | 1.43 | .82 | - | 1.61 | .99 | - | 2.20 | 1.30 |
| **Sample size** | 1,248 | | | 1,109 | | | 1,254 | | | 1,165 | | | 1,055 | | |

**Note.** *α*: Cronbach’s alpha. #: Pearson’s correlation coefficient.

**Appendix E: Results of randomization checks**

Study 1 and 2:

Randomization was successful in both experiments, with the four conditions not significantly different from one another on the key socio-demographics (Study 1: gender ($\chi^{2}$(3) = 0.76, *p* = .858), age (*F*(3, 1244) = 0.54, *p* = .654), ethnicity ($\chi^{2}$(15) = 8.74, *p* = .891), and education ($\chi^{2}$(18) = 14.80, *p* = .676). Study 2, gender ($\chi^{2}$(3) = 2.12, *p* = .549), age (*F*(3, 1105) = 2.87, *p* = .035), municipality size ($\chi^{2}$(12) = 9.74, *p* = .638), and education ($\chi^{2}$(15) = 10.80, *p* = .766).

Study 3:

Randomization was successful. Gender ($\chi^{2}$(5) = 3.84, *p* = .573), age (*F*(5, 1248) = 0.40, *p* = .847), and education ($\chi^{2}$(40) = 27.04, p = .941) were evenly distributed among the six experimental groups.

Study 4 and 5:

Randomization was successful in both experiments. In the US, gender ($\chi^{2}$(3) = 0.44, *p* = .932), age (*F*(3, 1161) = 0.33, *p* = .801), ethnicity ($\chi^{2}$(15) = 10.95, *p* = .756), and education ($\chi^{2}$(18) = 14.50, *p* = .696) were evenly distributed for four experimental groups. In Poland, gender ($\chi^{2}$(3) = 0.31, *p* = .958), age (*F*(3, 1051) = .89, *p* = .448), municipality size ($\chi^{2}$(12) = 14.93, *p* = .245), and education ($\chi^{2}$(15) = 9.59, *p* = .845) were also evenly distributed.

**Appendix F: Stimuli (Study 1 and 2)**

[Happy writing condition]

Now we would like you to describe something that made you feel happy and positive. Please describe how you felt as vividly and in as much detail as possible. Think about the specific context and the people involved (Examples of things you might write about include: having a great meal, receiving a gift, etc.). It is okay if you don’t remember all the details, just be specific about what exactly it was that made you happy and positive and what it felt like to be so happy and positive. Please write out your answer so that someone reading it might even feel how happy and positive you felt.

[Happy questions condition]

1. What is your favorite song?
2. What is your favorite food?
3. What is your favorite animal?
4. What is your favorite hobby?
5. What is your favorite travel destination?
6. What makes you most happy? It can be a place, a person, an activity, or anything else that makes you very happy.

[Happy photos condition]


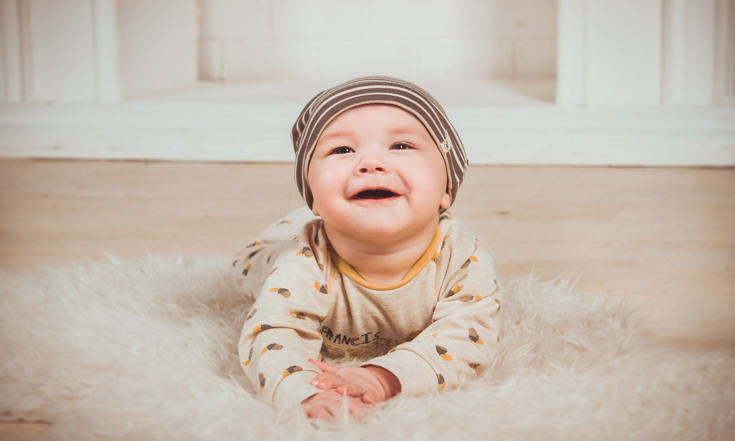


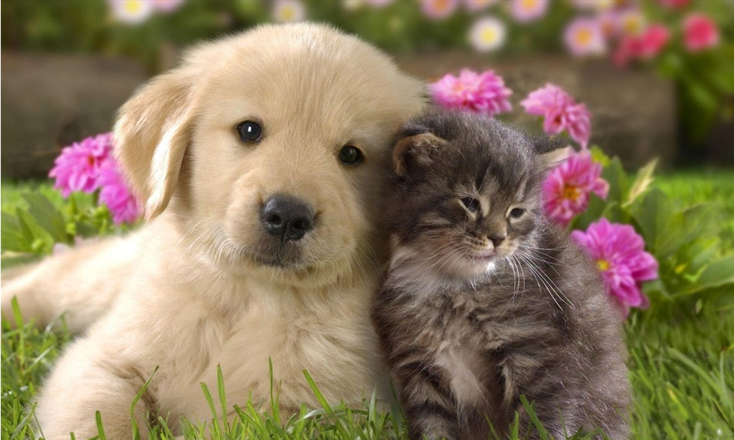


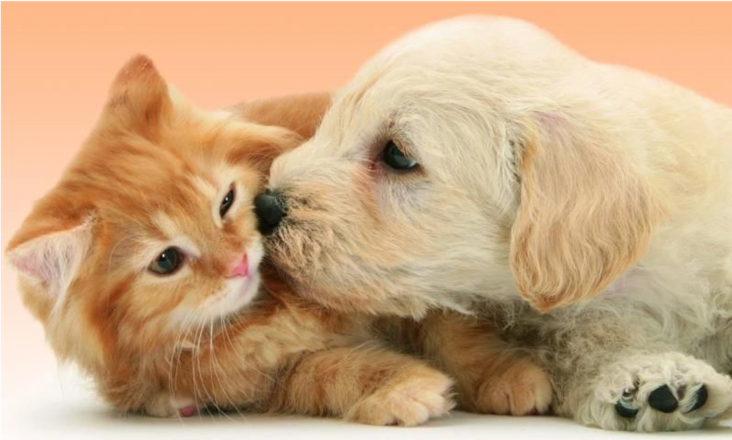


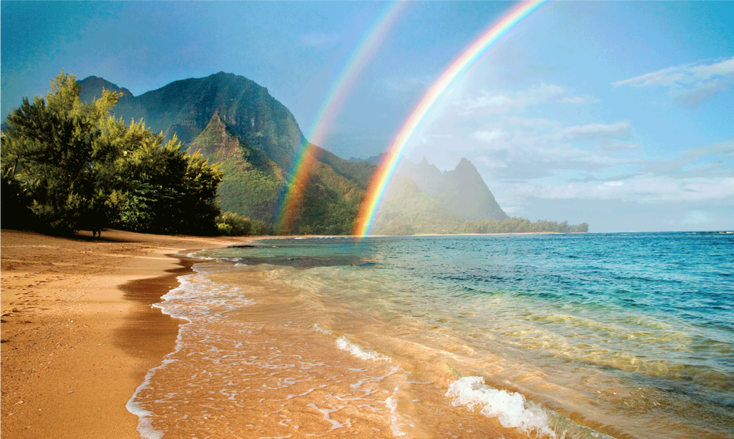


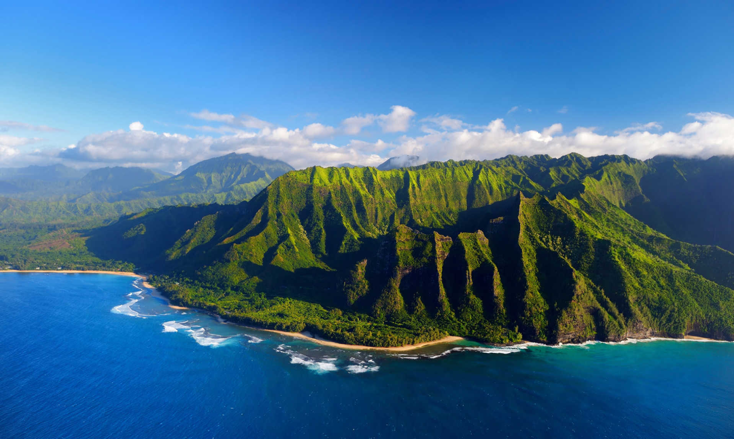


**Appendix G: Descriptives**

**Table G.1.1** Means and standard deviations of affective polarization, feeling thermometers toward social groups, conspiracy endorsement, and deep fake recognition by conditions (Study 1, US)

| Condition | Affective polarization | FT immigrants | FT feminists | FT neo-Nazis | Conspiracy | Deep fake |
| --- | --- | --- | --- | --- | --- | --- |
| Control | 0.371 (0.182) | 0.582 (0.282) | 0.539 (0.310) | 0.108 (0.224) | 0.363 (0.253) | 0.152 (0.244) |
| Happy Photos | 0.361 (0.183) | 0.585 (0.280) | 0.541 (0.304) | 0.111 (0.217) | 0.318 (0.233) | 0.142 (0.239) |
| Happy Questions | 0.386 (0.193) | 0.588 (0.278) | 0.537 (0.317) | 0.106 (0.218) | 0.368 (0.243) | 0.162 (0.253) |
| Happy Writing | 0.380 (0.174) | 0.606 (0.272) | 0.567 (0.297) | 0.098 (0.194) | 0.337 (0.243) | 0.138 (0.226) |

Note. Cell entries are means with associated standard deviations in parentheses. FT = feeling thermometer.

**Table G.1.2** Means and standard deviations of individual and subtractive affective polarization indicators by conditions (Study 1, US)

| Condition | FT outparty | FT outparty supporters | FT out-ideologues | Social distance | Trait ratings | Outgroup trust | FT party (subt.) | FT party supporters (subt.) | FT ideology (subt.) |
| --- | --- | --- | --- | --- | --- | --- | --- | --- | --- |
| Control | 0.217 (0.229) | 0.267 (0.241) | 0.311 (0.286) | 0.585 (0.263) | 0.405 (0.186) | 0.411 (0.250) | 0.787 (0.167) | 0.758 (0.177) | 0.730 (0.215) |
| Happy Photos | 0.224 (0.248) | 0.265 (0.240) | 0.320 (0.284) | 0.553 (0.254) | 0.394 (0.185) | 0.386 (0.238) | 0.777 (0.176) | 0.756 (0.177) | 0.718 (0.209) |
| Happy Questions | 0.232 (0.251) | 0.279 (0.253) | 0.343 (0.279) | 0.607 (0.259) | 0.424 (0.196) | 0.414 (0.264) | 0.769 (0.185) | 0.749 (0.188) | 0.700 (0.221) |
| Happy Writing | 0.226 (0.235) | 0.265 (0.228) | 0.309 (0.262) | 0.610 (0.240) | 0.414 (0.184) | 0.434 (0.249) | 0.778 (0.176) | 0.761 (0.178) | 0.730 (0.210) |

Note. Cell entries are means with associated standard deviations in parentheses. FT = feeling thermometer.

**Table G.2.1** Means and standard deviations of affective polarization, feeling thermometers toward social groups, conspiracy endorsement, and deep fake recognition by conditions (Study 2, PL)

| Condition | Affective polarization | FT immigrants | FT feminists | FT neo-Nazis | FT nationalists | Conspiracy | Deep fake |
| --- | --- | --- | --- | --- | --- | --- | --- |
| Control | 0.301 (0.152) | 0.391 (0.261) | 0.477 (0.286) | 0.074 (0.153) | 0.280 (0.267) | 0.410 (0.204) | 0.296 (0.338) |
| Happy Photos | 0.305 (0.143) | 0.397 (0.265) | 0.467 (0.293) | 0.072 (0.150) | 0.261 (0.272) | 0.398 (0.200) | 0.338 (0.356) |
| Happy Questions | 0.328 (0.169) | 0.382 (0.273) | 0.459 (0.321) | 0.077 (0.144) | 0.284 (0.272) | 0.399 (0.198) | 0.280 (0.318) |
| Happy Writing | 0.305 (0.146) | 0.397 (0.265) | 0.441 (0.281) | 0.080 (0.161) | 0.288 (0.282) | 0.422 (0.186) | 0.288 (0.311) |

Note. Cell entries are means with associated standard deviations in parentheses. FT = feeling thermometer.

**Table G.2.2** Means and standard deviations of individual and subtractive affective polarization indicators by conditions (Study 2, PL)

| Condition | FT outparty | FT outparty supporters | FT out-gov. | FT out-ideologues | Social distance | Trait ratings | Outgroup trust | FT party (subt.) | FT party supporters (subt.) | FT gov. (subt.) | FT ideology (subt.) |
| --- | --- | --- | --- | --- | --- | --- | --- | --- | --- | --- | --- |
| Control | 0.153 (0.227) | 0.167 (0.221) | 0.222 (0.238) | 0.290 (0.231) | 0.535 (0.270) | 0.394 (0.192) | 0.331 (0.227) | 0.763 (0.209) | 0.775 (0.189) | 0.741 (0.204) | 0.707 (0.184) |
| Happy Photos | 0.148 (0.217) | 0.179 (0.230) | 0.210 (0.215) | 0.331 (0.225) | 0.536 (0.242) | 0.402 (0.179) | 0.332 (0.219) | 0.762 (0.206) | 0.776 (0.187) | 0.752 (0.200) | 0.704 (0.180) |
| Happy Questions | 0.162 (0.227) | 0.198 (0.250) | 0.258 (0.261) | 0.335 (0.246) | 0.550 (0.262) | 0.430 (0.203) | 0.365 (0.238) | 0.752 (0.217) | 0.754 (0.204) | 0.713 (0.230) | 0.689 (0.184) |
| Happy Writing | 0.162 (0.236) | 0.180 (0.238) | 0.209 (0.213) | 0.299 (0.223) | 0.522 (0.243) | 0.403 (0.178) | 0.351 (0.213) | 0.762 (0.212) | 0.776 (0.189) | 0.762 (0.195) | 0.716 (0.198) |

Note. Cell entries are means with associated standard deviations in parentheses. FT = feeling thermometer.

**Table G.3.1** Means and standard deviations of affective polarization, feeling thermometers toward social groups, conspiracy endorsement, and deep fake recognition by conditions (Study 3, NL)

| Condition | Affective polarization | FT immigrants | FT feminists | FT nationalists | Conspiracy | Deep fake |
| --- | --- | --- | --- | --- | --- | --- |
| Control | 0.381 (0.124) | 0.532 (0.216) | 0.534 (0.231) | 0.437 (0.224) | 0.353 (0.228) | 0.109 (0.206) |
| Happy Photos | 0.376 (0.116) | 0.544 (0.208) | 0.539 (0.230) | 0.452 (0.216) | 0.360 (0.240) | 0.091 (0.183) |
| Happy Questions | 0.383 (0.129) | 0.549 (0.221) | 0.550 (0.228) | 0.438 (0.233) | 0.368 (0.213) | 0.135 (0.237) |
| Happy Writing | 0.391 (0.126) | 0.581 (0.195) | 0.562 (0.200) | 0.437 (0.225) | 0.328 (0.215) | 0.094 (0.192) |

Note. Cell entries are means with associated standard deviations in parentheses. FT = feeling thermometer.

**Table G.3.2** Means and standard deviations of individual and subtractive affective polarization indicators by conditions (Study 3, NL)

| Condition | FT outparty | FT outparty supporters | FT out-ideologues | Social distance | Trait ratings | Outgroup trust | FT party (subt.) | FT party supporters (subt.) | FT ideology (subt.) |
| --- | --- | --- | --- | --- | --- | --- | --- | --- | --- |
| Control | 0.145 (0.192) | 0.189 (0.204) | 0.375 (0.190) | 0.585 (0.196) | 0.491 (0.136) | 0.502 (0.191) | 0.808 (0.141) | 0.771 (0.147) | 0.611 (0.155) |
| Happy Photos | 0.145 (0.191) | 0.169 (0.193) | 0.369 (0.191) | 0.585 (0.191) | 0.486 (0.144) | 0.500 (0.192) | 0.812 (0.133) | 0.780 (0.135) | 0.623 (0.156) |
| Happy Questions | 0.150 (0.203) | 0.193 (0.209) | 0.376 (0.198) | 0.608 (0.208) | 0.482 (0.142) | 0.487 (0.194) | 0.799 (0.156) | 0.762 (0.162) | 0.608 (0.172) |
| Happy Writing | 0.162 (0.201) | 0.195 (0.200) | 0.381 (0.177) | 0.595 (0.207) | 0.492 (0.137) | 0.520 (0.178) | 0.800 (0.150) | 0.766 (0.150) | 0.607 (0.158) |

Note. Cell entries are means with associated standard deviations in parentheses. FT = feeling thermometer.

**Table G.4.1** Means and standard deviations of affective polarization, feeling thermometers toward social groups, conspiracy endorsement, and deep fake recognition by conditions (Study 4, US)

| Condition | Affective polarization | FT immigrants | FT feminists | FT neo-Nazis | Conspiracy | Deep fake |
| --- | --- | --- | --- | --- | --- | --- |
| Control Writing | 0.410 (0.192) | 0.608 (0.269) | 0.498 (0.288) | 0.126 (0.233) | 0.373 (0.235) | 0.142 (0.236) |
| Angry Writing | 0.406 (0.194) | 0.610 (0.275) | 0.537 (0.276) | 0.135 (0.240) | 0.378 (0.237) | 0.175 (0.268) |
| Anxious Writing | 0.401 (0.196) | 0.577 (0.274) | 0.498 (0.300) | 0.125 (0.230) | 0.384 (0.237) | 0.158 (0.246) |
| Happy Writing | 0.409 (0.196) | 0.604 (0.264) | 0.514 (0.279) | 0.123 (0.228) | 0.397 (0.236) | 0.137 (0.241) |

Note. Cell entries are means with associated standard deviations in parentheses. FT = feeling thermometer.

**Table G.4.2** Means and standard deviations of individual and subtractive affective polarization indicators by conditions (Study 4, US)

| Condition | FT outparty | FT outparty supporters | FT out-ideologues | Social distance | Trait ratings | Outgroup trust | FT party (subt.) | FT party supporters (subt.) | FT ideology (subt.) |
| --- | --- | --- | --- | --- | --- | --- | --- | --- | --- |
| Control Writing | 0.263 (0.261) | 0.302 (0.245) | 0.371 (0.277) | 0.635 (0.240) | 0.434 (0.212) | 0.436 (0.263) | 0.756 (0.182) | 0.742 (0.180) | 0.692 (0.192) |
| Angry Writing | 0.256 (0.265) | 0.292 (0.257) | 0.346 (0.270) | 0.642 (0.249) | 0.420 (0.204) | 0.449 (0.256) | 0.768 (0.191) | 0.752 (0.186) | 0.705 (0.205) |
| Anxious Writing | 0.264 (0.265) | 0.303 (0.263) | 0.358 (0.299) | 0.610 (0.257) | 0.419 (0.202) | 0.426 (0.255) | 0.764 (0.193) | 0.747 (0.190) | 0.700 (0.230) |
| Happy Writing | 0.263 (0.251) | 0.311 (0.257) | 0.365 (0.285) | 0.630 (0.243) | 0.428 (0.202) | 0.439 (0.266) | 0.765 (0.174) | 0.745 (0.172) | 0.688 (0.201) |

Note. Cell entries are means with associated standard deviations in parentheses. FT = feeling thermometer.

**Table G.5.1** Means and standard deviations of affective polarization, feeling thermometers toward social groups, conspiracy endorsement, and deep fake recognition by conditions (Study 5, PL)

| Condition | Affective polarization | FT immigrants | FT feminists | FT neo-Nazis | FT nationalists | Conspiracy | Deep fake |
| --- | --- | --- | --- | --- | --- | --- | --- |
| Control Writing | 0.310 (0.154) | 0.427 (0.264) | 0.466 (0.278) | 0.101 (0.182) | 0.266 (0.272) | 0.413 (0.197) | 0.271 (0.305) |
| Angry Writing | 0.309 (0.154) | 0.412 (0.267) | 0.478 (0.295) | 0.123 (0.196) | 0.305 (0.281) | 0.427 (0.189) | 0.328 (0.346) |
| Anxious Writing | 0.316 (0.161) | 0.406 (0.266) | 0.474 (0.295) | 0.104 (0.193) | 0.292 (0.284) | 0.416 (0.191) | 0.282 (0.320) |
| Happy Writing | 0.324 (0.160) | 0.428 (0.265) | 0.491 (0.297) | 0.135 (0.228) | 0.288 (0.269) | 0.405 (0.193) | 0.322 (0.325) |

Note. Cell entries are means with associated standard deviations in parentheses. FT = feeling thermometer.

**Table G.5.2** Means and standard deviations of individual and subtractive affective polarization indicators by conditions (Study 5, PL)

| Condition | FT outparty | FT outparty supporters | FT out-gov. | FT out-ideologues | Social distance | Trait ratings | Outgroup trust | FT party (subt.) | FT party supporters (subt.) | FT gov. (subt.) | FT ideology (subt.) |
| --- | --- | --- | --- | --- | --- | --- | --- | --- | --- | --- | --- |
| Control Writing | 0.150 (0.207) | 0.186 (0.227) | 0.239 (0.229) | 0.318 (0.213) | 0.530 (0.242) | 0.398 (0.195) | 0.347 (0.223) | 0.772 (0.189) | 0.779 (0.177) | 0.720 (0.212) | 0.689 (0.181) |
| Angry Writing | 0.168 (0.223) | 0.188 (0.227) | 0.244 (0.225) | 0.310 (0.233) | 0.525 (0.232) | 0.392 (0.189) | 0.337 (0.231) | 0.752 (0.201) | 0.765 (0.181) | 0.728 (0.202) | 0.690 (0.187) |
| Anxious Writing | 0.156 (0.234) | 0.184 (0.236) | 0.257 (0.241) | 0.330 (0.243) | 0.530 (0.247) | 0.406 (0.194) | 0.355 (0.231) | 0.772 (0.197) | 0.770 (0.186) | 0.716 (0.197) | 0.671 (0.199) |
| Happy Writing | 0.177 (0.221) | 0.193 (0.220) | 0.260 (0.233) | 0.334 (0.225) | 0.548 (0.254) | 0.414 (0.192) | 0.346 (0.226) | 0.764 (0.191) | 0.773 (0.179) | 0.709 (0.202) | 0.694 (0.173) |

Note. Cell entries are means with associated standard deviations in parentheses. FT = feeling thermometer.

**Appendix H: Regression models**

**Table H.1.1** The effects of happiness treatments on affective polarization, feeling thermometers toward social groups, conspiracy endorsement, and deep fake recognition (Study 1, US)

|  | Affective polarization | FT immigrants | FT feminists | FT neo-Nazis | Conspiracy | Deep fake |
| --- | --- | --- | --- | --- | --- | --- |
| Intercept | 0.371* (0.010) | 0.582* (0.016) | 0.539* (0.017) | 0.108* (0.012) | 0.363* (0.014) | 0.152* (0.014) |
| Happy Photos | -0.010 (0.015) | 0.003 (0.022) | 0.003 (0.025) | 0.003 (0.017) | -0.045 (0.019) | -0.011 (0.019) |
| Happy Questions | 0.015 (0.015) | 0.006 (0.022) | -0.001 (0.025) | -0.002 (0.017) | 0.005 (0.019) | 0.009 (0.019) |
| Happy Writing | 0.009 (0.015) | 0.024 (0.022) | 0.028 (0.025) | -0.010 (0.017) | -0.025 (0.020) | -0.014 (0.019) |
| N | 1248 | 1248 | 1248 | 1248 | 1248 | 1248 |
| R-squared | 0.003 | 0.001 | 0.002 | 0.001 | 0.007 | 0.001 |

Note. Cell entries are OLS regression coefficients with associated standard errors in parentheses. FT = feeling thermometer. * FDR adjusted *p* < .05.

**Table H.1.2** The effects of happiness treatments on individual and subtractive affective polarization indicators (Study 1, US)

|  | FT outparty | FT outparty supporters | FT out-ideologues | Social distance | Trait ratings | Outgroup trust | FT party (subt.) | FT party supporters (subt.) | FT ideology (subt.) |
| --- | --- | --- | --- | --- | --- | --- | --- | --- | --- |
| Intercept | 0.217* (0.014) | 0.267* (0.014) | 0.311* (0.018) | 0.585* (0.014) | 0.405* (0.011) | 0.411* (0.014) | 0.787* (0.010) | 0.758* (0.010) | 0.730* (0.014) |
| Happy Photos | 0.006 (0.019) | -0.002 (0.019) | 0.009 (0.026) | -0.032 (0.020) | -0.012 (0.015) | -0.025 (0.020) | -0.010 (0.014) | -0.002 (0.014) | -0.011 (0.020) |
| Happy Questions | 0.015 (0.019) | 0.012 (0.019) | 0.032 (0.026) | 0.022 (0.020) | 0.019 (0.015) | 0.003 (0.020) | -0.017 (0.014) | -0.009 (0.014) | -0.030 (0.020) |
| Happy Writing | 0.008 (0.019) | -0.002 (0.019) | -0.002 (0.026) | 0.025 (0.020) | 0.009 (0.015) | 0.023 (0.020) | -0.009 (0.014) | 0.003 (0.014) | 0.000 (0.020) |
| *N* | 1248 | 1248 | 930 | 1248 | 1248 | 1248 | 1248 | 1248 | 930 |
| *R*-squared | 0.000 | 0.001 | 0.002 | 0.008 | 0.003 | 0.005 | 0.001 | 0.001 | 0.003 |

Note. Cell entries are OLS regression coefficients with associated standard errors in parentheses. FT = feeling thermometer. * FDR adjusted *p* < .05.

**Table H.1.3** The effects of interactions between happiness treatments and political identity strength on affective polarization, feeling thermometers toward social groups, conspiracy endorsement, and deep fake recognition (Study 1, US)

|  | Affective polarization | FT immigrants | FT feminists | FT neo-Nazis | Conspiracy | Deep fake |
| --- | --- | --- | --- | --- | --- | --- |
| Intercept | 0.530* (0.037) | 0.511* (0.058) | 0.458* (0.064) | 0.025 (0.045) | 0.251* (0.051) | 0.129 (0.051) |
| Happy Photos | 0.002 (0.051) | 0.025 (0.080) | -0.004 (0.088) | 0.132 (0.061) | 0.050 (0.069) | -0.020 (0.069) |
| Happy Questions | -0.018 (0.050) | 0.037 (0.078) | 0.013 (0.086) | 0.054 (0.060) | -0.009 (0.068) | 0.011 (0.068) |
| Happy Writing | 0.012 (0.051) | -0.041 (0.080) | -0.045 (0.089) | 0.054 (0.062) | 0.042 (0.070) | -0.042 (0.070) |
| Pids | -0.233* (0.052) | 0.104 (0.082) | 0.117 (0.090) | 0.121 (0.063) | 0.163 (0.071) | 0.034 (0.071) |
| Happy Photos ×  Pids | -0.021 (0.072) | -0.031 (0.112) | 0.012 (0.124) | -0.190 (0.086) | -0.138 (0.098) | 0.015 (0.098) |
| Happy Questions ×  Pids | 0.044 (0.071) | -0.045 (0.111) | -0.018 (0.122) | -0.081 (0.085) | 0.025 (0.096) | -0.002 (0.096) |
| Happy Writing ×  Pids | -0.006 (0.072) | 0.096 (0.113) | 0.108 (0.124) | -0.094 (0.087) | -0.098 (0.098) | 0.041 (0.098) |
| *N* | 1248 | 1248 | 1248 | 1248 | 1248 | 1248 |
| *R*-squared | 0.067 | 0.009 | 0.011 | 0.005 | 0.018 | 0.003 |

Note. Cell entries are OLS regression coefficients with associated standard errors in parentheses. FT = feeling thermometer. * FDR adjusted *p* < .05.

**Table H.1.4** The effects of interactions between happiness treatments and political identity strength on individual and subtractive affective polarization indicators (Study 1, US)

|  | FT outparty | FT outparty supporters | FT out-ideologues | Social distance | Trait ratings | Outgroup trust | FT party (subt.) | FT party supporters (subt.) | FT ideology (subt.) |
| --- | --- | --- | --- | --- | --- | --- | --- | --- | --- |
| Intercept | 0.393* (0.049) | 0.451* (0.049) | 0.393* (0.069) | 0.744* (0.053) | 0.549* (0.038) | 0.562* (0.052) | 0.493* (0.032) | 0.465* (0.033) | 0.510* (0.053) |
| Happy Photos | 0.060 (0.067) | 0.048 (0.067) | 0.055 (0.097) | -0.072 (0.072) | -0.021 (0.053) | -0.020 (0.071) | -0.022 (0.044) | -0.019 (0.045) | 0.063 (0.074) |
| Happy Questions | -0.004 (0.066) | -0.004 (0.066) | -0.095 (0.097) | -0.071 (0.071) | 0.020 (0.052) | 0.019 (0.070) | -0.010 (0.043) | 0.002 (0.044) | 0.124 (0.073) |
| Happy Writing | 0.085 (0.068) | 0.059 (0.067) | 0.066 (0.099) | -0.112 (0.073) | 0.030 (0.053) | 0.009 (0.071) | -0.025 (0.044) | 0.003 (0.046) | 0.016 (0.075) |
| Pids | -0.256* (0.069) | -0.268* (0.068) | -0.115 (0.094) | -0.232* (0.074) | -0.210* (0.054) | -0.220* (0.073) | 0.428* (0.045) | 0.427* (0.046) | 0.308* (0.071) |
| Happy Photos ×  Pids | -0.083 (0.095) | -0.077 (0.094) | -0.067 (0.133) | 0.057 (0.102) | 0.011 (0.074) | -0.011 (0.100) | 0.023 (0.062) | 0.031 (0.064) | -0.101 (0.101) |
| Happy Questions ×  Pids | 0.022 (0.093) | 0.017 (0.093) | 0.180 (0.131) | 0.133 (0.101) | -0.007 (0.073) | -0.030 (0.098) | 0.000 (0.061) | -0.007 (0.063) | -0.214 (0.100) |
| Happy Writing ×  Pids | -0.113 (0.095) | -0.091 (0.095) | -0.093 (0.134) | 0.199 (0.103) | -0.033 (0.074) | 0.019 (0.100) | 0.026 (0.062) | 0.001 (0.064) | -0.025 (0.101) |
| *N* | 1248 | 1248 | 930 | 1248 | 1248 | 1248 | 1248 | 1248 | 930 |
| *R*-squared | 0.066 | 0.068 | 0.014 | 0.022 | 0.058 | 0.038 | 0.257 | 0.239 | 0.049 |

Note. Cell entries are OLS regression coefficients with associated standard errors in parentheses. FT = feeling thermometer. * FDR adjusted *p* < .05.

**Table H.1.5** The effects of interactions between happiness treatments and political interest on affective polarization, feeling thermometers toward social groups, conspiracy endorsement, and deep fake recognition (Study 1, US)

|  | Affective polarization | FT immigrants | FT feminists | FT neo-Nazis | Conspiracy | Deep fake |
| --- | --- | --- | --- | --- | --- | --- |
| Intercept | 0.414* (0.025) | 0.482* (0.037) | 0.487* (0.041) | 0.134* (0.029) | 0.433* (0.033) | 0.189* (0.032) |
| Happy Photos | 0.014 (0.034) | 0.063 (0.052) | -0.022 (0.057) | 0.004 (0.040) | -0.097 (0.045) | -0.013 (0.045) |
| Happy Questions | -0.023 (0.034) | 0.039 (0.052) | 0.058 (0.058) | -0.018 (0.040) | -0.053 (0.046) | -0.052 (0.046) |
| Happy Writing | 0.017 (0.036) | 0.082 (0.055) | 0.023 (0.061) | -0.040 (0.043) | -0.064 (0.048) | -0.068 (0.048) |
| Interest | -0.069 (0.036) | 0.161* (0.054) | 0.083 (0.060) | -0.041 (0.042) | -0.113 (0.048) | -0.058 (0.047) |
| Happy Photos ×  Interest | -0.042 (0.050) | -0.093 (0.076) | 0.044 (0.085) | -0.004 (0.059) | 0.082 (0.067) | 0.002 (0.066) |
| Happy Questions ×  Interest | 0.061 (0.050) | -0.053 (0.076) | -0.094 (0.085) | 0.026 (0.059) | 0.093 (0.067) | 0.099 (0.067) |
| Happy Writing ×  Interest | -0.010 (0.052) | -0.096 (0.079) | 0.004 (0.088) | 0.047 (0.061) | 0.065 (0.069) | 0.085 (0.069) |
| *N* | 1248 | 1248 | 1248 | 1248 | 1248 | 1248 |
| *R*-squared | 0.017 | 0.014 | 0.008 | 0.002 | 0.013 | 0.005 |

Note. Cell entries are OLS regression coefficients with associated standard errors in parentheses. FT = feeling thermometer. * FDR adjusted *p* < .05.

**Table H.1.6** The effects of interactions between happiness treatments and political interest on individual and subtractive affective polarization indicators (Study 1, US)

|  | FT outparty | FT outparty supporters | FT out-ideologues | Social distance | Trait ratings | Outgroup trust | FT party (subt.) | FT party supporters (subt.) | FT ideology (subt.) |
| --- | --- | --- | --- | --- | --- | --- | --- | --- | --- |
| Intercept | 0.293* (0.032) | 0.311* (0.032) | 0.390* (0.049) | 0.564* (0.034) | 0.458* (0.025) | 0.431* (0.034) | 0.688* (0.023) | 0.669* (0.024) | 0.596* (0.037) |
| Happy Photos | 0.005 (0.045) | 0.027 (0.045) | 0.073 (0.067) | 0.017 (0.048) | -0.004 (0.035) | 0.011 (0.047) | -0.010 (0.032) | -0.018 (0.033) | -0.037 (0.051) |
| Happy Questions | -0.041 (0.045) | -0.032 (0.045) | -0.053 (0.068) | 0.017 (0.048) | 0.012 (0.035) | -0.043 (0.047) | 0.027 (0.033) | 0.017 (0.033) | 0.010 (0.051) |
| Happy Writing | 0.013 (0.048) | 0.032 (0.048) | 0.009 (0.069) | 0.013 (0.051) | 0.009 (0.037) | 0.045 (0.050) | 0.006 (0.034) | -0.008 (0.035) | -0.001 (0.052) |
| Interest | -0.122 (0.047) | -0.070 (0.047) | -0.117 (0.067) | 0.033 (0.050) | -0.085 (0.037) | -0.031 (0.049) | 0.159* (0.034) | 0.143* (0.034) | 0.197* (0.050) |
| Happy Photos ×  Interest | -0.003 (0.066) | -0.050 (0.066) | -0.103 (0.093) | -0.080 (0.070) | -0.017 (0.052) | -0.061 (0.069) | 0.005 (0.047) | 0.034 (0.048) | 0.048 (0.070) |
| Happy Questions ×  Interest | 0.089 (0.066) | 0.070 (0.066) | 0.126 (0.093) | 0.009 (0.070) | 0.009 (0.052) | 0.073 (0.069) | -0.070 (0.047) | -0.041 (0.048) | -0.056 (0.070) |
| Happy Writing ×  Interest | -0.002 (0.069) | -0.049 (0.069) | -0.014 (0.094) | 0.017 (0.073) | 0.003 (0.053) | -0.033 (0.072) | -0.030 (0.049) | 0.010 (0.050) | -0.003 (0.071) |
| *N* | 1248 | 1248 | 930 | 1248 | 1248 | 1248 | 1248 | 1248 | 930 |
| *R*-squared | 0.017 | 0.012 | 0.022 | 0.010 | 0.021 | 0.010 | 0.052 | 0.055 | 0.068 |

Note. Cell entries are OLS regression coefficients with associated standard errors in parentheses. FT = feeling thermometer. * FDR adjusted *p* < .05.

**Table H.1.7** The effects of interactions between happiness treatments and partisanship on affective polarization, feeling thermometers toward social groups, conspiracy endorsement, and deep fake recognition (Study 1, US)

|  | Affective polarization | FT immigrants | FT feminists | FT neo-Nazis | Conspiracy | Deep fake |
| --- | --- | --- | --- | --- | --- | --- |
| Intercept | 0.365* (0.014) | 0.693* (0.019) | 0.677* (0.021) | 0.086* (0.017) | 0.319* (0.018) | 0.165* (0.019) |
| Happy Photos | 0.003 (0.020) | 0.020 (0.027) | 0.011 (0.029) | 0.023 (0.024) | -0.064 (0.026) | 0.012 (0.027) |
| Happy Questions | 0.023 (0.020) | 0.006 (0.027) | -0.001 (0.029) | 0.010 (0.023) | -0.003 (0.026) | 0.001 (0.026) |
| Happy Writing | 0.010 (0.020) | 0.048 (0.027) | 0.053 (0.029) | 0.000 (0.024) | -0.016 (0.026) | -0.013 (0.027) |
| Partisanship (R) | 0.012 (0.021) | -0.236* (0.028) | -0.296* (0.030) | 0.047 (0.024) | 0.093* (0.027) | -0.027 (0.027) |
| Happy Photos ×  Partisanship (R) | -0.026 (0.029) | -0.020 (0.040) | 0.000 (0.042) | -0.044 (0.034) | 0.032 (0.038) | -0.044 (0.039) |
| Happy Questions ×  Partisanship (R) | -0.016 (0.029) | -0.015 (0.040) | -0.019 (0.043) | -0.024 (0.034) | 0.024 (0.038) | 0.018 (0.039) |
| Happy Writing ×  Partisanship (R) | -0.002 (0.030) | -0.050 (0.040) | -0.051 (0.043) | -0.022 (0.034) | -0.021 (0.038) | -0.003 (0.039) |
| *N* | 1248 | 1248 | 1248 | 1248 | 1248 | 1248 |
| *R*-squared | 0.004 | 0.216 | 0.261 | 0.005 | 0.053 | 0.009 |

Note. Cell entries are OLS regression coefficients with associated standard errors in parentheses. FT = feeling thermometer. * FDR adjusted *p* < .05.

**Table H.1.8** The effects of interactions between happiness treatments and partisanship on individual and subtractive affective polarization indicators (Study 1, US)

|  | FT outparty | FT outparty supporters | FT out-ideologues | Social distance | Trait ratings | Outgroup trust | FT party (subt.) | FT party supporters (subt.) | FT ideology (subt.) |
| --- | --- | --- | --- | --- | --- | --- | --- | --- | --- |
| Intercept | 0.212* (0.019) | 0.260* (0.019) | 0.373* (0.026) | 0.549* (0.020) | 0.397* (0.015) | 0.401* (0.020) | 0.781* (0.014) | 0.752* (0.014) | 0.678* (0.020) |
| Happy Photos | 0.035 (0.027) | 0.011 (0.027) | 0.017 (0.036) | -0.022 (0.028) | -0.016 (0.021) | -0.013 (0.028) | -0.022 (0.020) | 0.000 (0.020) | -0.009 (0.028) |
| Happy Questions | 0.026 (0.026) | 0.024 (0.026) | 0.034 (0.035) | 0.020 (0.027) | 0.029 (0.020) | 0.007 (0.027) | -0.008 (0.019) | 0.001 (0.020) | -0.019 (0.027) |
| Happy Writing | 0.013 (0.027) | 0.003 (0.027) | -0.015 (0.035) | 0.028 (0.028) | 0.002 (0.021) | 0.019 (0.028) | 0.004 (0.019) | 0.014 (0.020) | 0.017 (0.027) |
| Partisanship (R) | 0.012 (0.027) | 0.015 (0.027) | -0.123* (0.036) | 0.076* (0.029) | 0.018 (0.021) | 0.023 (0.029) | 0.012 (0.020) | 0.013 (0.021) | 0.103* (0.028) |
| Happy Photos ×  Partisanship (R) | -0.059 (0.039) | -0.026 (0.039) | -0.011 (0.051) | -0.023 (0.040) | 0.008 (0.030) | -0.025 (0.040) | 0.023 (0.028) | -0.003 (0.029) | -0.007 (0.039) |
| Happy Questions ×  Partisanship (R) | -0.024 (0.039) | -0.026 (0.039) | -0.015 (0.051) | 0.008 (0.040) | -0.023 (0.030) | -0.009 (0.040) | -0.021 (0.028) | -0.023 (0.029) | -0.014 (0.039) |
| Happy Writing ×  Partisanship (R) | -0.011 (0.039) | -0.011 (0.039) | 0.024 (0.050) | -0.007 (0.041) | 0.013 (0.030) | 0.007 (0.040) | -0.029 (0.028) | -0.024 (0.029) | -0.033 (0.039) |
| *N* | 1248 | 1248 | 930 | 1248 | 1248 | 1248 | 1248 | 1248 | 930 |
| *R*-squared | 0.003 | 0.001 | 0.052 | 0.027 | 0.007 | 0.006 | 0.005 | 0.002 | 0.048 |

Note. Cell entries are OLS regression coefficients with associated standard errors in parentheses. FT = feeling thermometer. * FDR adjusted *p* < .05.

**Table H.1.9** The effects of interactions between happiness treatments and ideology on affective polarization, feeling thermometers toward social groups, conspiracy endorsement, and deep fake recognition (Study 1, US)

|  | Affective polarization | FT immigrants | FT feminists | FT neo-Nazis | Conspiracy | Deep fake |
| --- | --- | --- | --- | --- | --- | --- |
| Intercept | 0.370* (0.023) | 0.782* (0.032) | 0.812* (0.034) | 0.010 (0.026) | 0.146* (0.027) | 0.139* (0.030) |
| Happy Photos | -0.017 (0.032) | 0.023 (0.045) | 0.028 (0.048) | 0.040 (0.037) | -0.021 (0.038) | 0.030 (0.043) |
| Happy Questions | -0.026 (0.032) | -0.024 (0.045) | -0.003 (0.047) | 0.006 (0.036) | -0.024 (0.038) | -0.015 (0.042) |
| Happy Writing | -0.027 (0.031) | 0.064 (0.044) | 0.032 (0.046) | -0.005 (0.036) | -0.016 (0.037) | 0.011 (0.041) |
| Ideology | 0.002 (0.035) | -0.345* (0.049) | -0.471* (0.052) | 0.168* (0.040) | 0.373* (0.041) | 0.023 (0.046) |
| Happy Photos ×  Ideology | 0.012 (0.050) | -0.044 (0.071) | -0.055 (0.075) | -0.061 (0.058) | -0.033 (0.059) | -0.071 (0.066) |
| Happy Questions ×  Ideology | 0.074 (0.049) | 0.044 (0.069) | -0.008 (0.073) | -0.009 (0.057) | 0.061 (0.058) | 0.044 (0.065) |
| Happy Writing ×  Ideology | 0.064 (0.049) | -0.088 (0.068) | -0.027 (0.072) | -0.001 (0.056) | 0.001 (0.058) | -0.045 (0.064) |
| *N* | 1248 | 1248 | 1248 | 1248 | 1248 | 1248 |
| *R*-squared | 0.010 | 0.156 | 0.226 | 0.046 | 0.221 | 0.004 |

Note. Cell entries are OLS regression coefficients with associated standard errors in parentheses. FT = feeling thermometer. * FDR adjusted *p* < .05.

**Table H.1.10** The effects of interactions between happiness treatments and ideology on individual and subtractive affective polarization indicators (Study 1, US)

|  | FT outparty | FT outparty supporters | FT out-ideologues | Social distance | Trait ratings | Outgroup trust | FT party (subt.) | FT party supporters (subt.) | FT ideology (subt.) |
| --- | --- | --- | --- | --- | --- | --- | --- | --- | --- |
| Intercept | 0.212* (0.030) | 0.235* (0.030) | 0.324* (0.037) | 0.510* (0.031) | 0.431* (0.023) | 0.462* (0.031) | 0.776* (0.022) | 0.753* (0.022) | 0.711* (0.029) |
| Happy Photos | 0.022 (0.043) | 0.025 (0.043) | -0.010 (0.053) | 0.000 (0.045) | -0.069 (0.033) | -0.061 (0.044) | -0.027 (0.031) | -0.009 (0.032) | 0.016 (0.041) |
| Happy Questions | -0.046 (0.042) | 0.000 (0.042) | -0.035 (0.052) | 0.035 (0.044) | -0.040 (0.033) | -0.060 (0.044) | 0.013 (0.031) | 0.010 (0.031) | 0.028 (0.040) |
| Happy Writing | -0.034 (0.041) | -0.009 (0.041) | -0.057 (0.051) | 0.035 (0.043) | -0.051 (0.032) | -0.031 (0.043) | 0.040 (0.030) | 0.035 (0.031) | 0.074 (0.039) |
| Ideology | 0.009 (0.046) | 0.054 (0.046) | -0.021 (0.054) | 0.129* (0.048) | -0.045 (0.036) | -0.088 (0.048) | 0.019 (0.034) | 0.009 (0.034) | 0.030 (0.041) |
| Happy Photos ×  Ideology | -0.027 (0.066) | -0.046 (0.066) | 0.031 (0.077) | -0.052 (0.070) | 0.100 (0.052) | 0.062 (0.069) | 0.030 (0.049) | 0.014 (0.050) | -0.045 (0.060) |
| Happy Questions ×  Ideology | 0.109 (0.065) | 0.022 (0.065) | 0.114 (0.076) | -0.019 (0.068) | 0.102 (0.051) | 0.108 (0.068) | -0.054 (0.048) | -0.035 (0.049) | -0.097 (0.058) |
| Happy Writing ×  Ideology | 0.076 (0.064) | 0.014 (0.064) | 0.094 (0.075) | -0.012 (0.067) | 0.105 (0.050) | 0.093 (0.067) | -0.087 (0.047) | -0.057 (0.048) | -0.126 (0.057) |
| *N* | 1248 | 1248 | 930 | 1248 | 1248 | 1248 | 1248 | 1248 | 930 |
| *R*-squared | 0.009 | 0.006 | 0.008 | 0.024 | 0.011 | 0.008 | 0.007 | 0.003 | 0.013 |

Note. Cell entries are OLS regression coefficients with associated standard errors in parentheses. FT = feeling thermometer. * FDR adjusted *p* < .05.

**Table H.1.11** The effects of interactions between happiness treatments and happiness strength on affective polarization, feeling thermometers toward social groups, conspiracy endorsement, and deep fake recognition (Study 1, US)

|  | Affective polarization | FT immigrants | FT feminists | FT neo-Nazis | Conspiracy | Deep fake |
| --- | --- | --- | --- | --- | --- | --- |
| Intercept | 0.335* (0.036) | 0.506* (0.055) | 0.552* (0.061) | 0.036 (0.042) | 0.158* (0.048) | 0.137* (0.048) |
| Happy Photos | -0.021 (0.051) | 0.091 (0.077) | -0.115 (0.085) | -0.014 (0.059) | 0.081 (0.067) | -0.022 (0.067) |
| Happy Questions | -0.048 (0.050) | 0.008 (0.076) | 0.023 (0.084) | -0.030 (0.058) | 0.044 (0.066) | -0.032 (0.066) |
| Happy Writing | -0.017 (0.050) | 0.066 (0.076) | -0.026 (0.084) | -0.009 (0.058) | 0.147 (0.066) | -0.041 (0.066) |
| Happiness | 0.054 (0.053) | 0.116 (0.080) | -0.020 (0.089) | 0.109 (0.061) | 0.311* (0.069) | 0.023 (0.070) |
| Happy Photos ×  Happiness | 0.020 (0.075) | -0.134 (0.115) | 0.185 (0.127) | 0.031 (0.088) | -0.186 (0.099) | 0.019 (0.099) |
| Happy Questions ×  Happiness | 0.100 (0.073) | 0.000 (0.112) | -0.038 (0.124) | 0.048 (0.086) | -0.051 (0.096) | 0.066 (0.097) |
| Happy Writing ×  Happiness | 0.042 (0.074) | -0.063 (0.112) | 0.085 (0.124) | 0.002 (0.086) | -0.259* (0.097) | 0.044 (0.097) |
| *N* | 1248 | 1248 | 1248 | 1248 | 1248 | 1248 |
| *R*-squared | 0.015 | 0.005 | 0.005 | 0.015 | 0.037 | 0.004 |

Note. Cell entries are OLS regression coefficients with associated standard errors in parentheses. FT = feeling thermometer. * FDR adjusted *p* < .05.

**Table H.1.12** The effects of interactions between happiness treatments and happiness strength on individual and subtractive affective polarization indicators (Study 1, US)

|  | FT outparty | FT outparty supporters | FT out-ideologues | Social distance | Trait ratings | Outgroup trust | FT party (subt.) | FT party supporters (subt.) | FT ideology (subt.) |
| --- | --- | --- | --- | --- | --- | --- | --- | --- | --- |
| Intercept | 0.185* (0.048) | 0.239* (0.048) | 0.169* (0.063) | 0.511* (0.050) | 0.401* (0.037) | 0.434* (0.050) | 0.698* (0.035) | 0.667* (0.036) | 0.763* (0.049) |
| Happy Photos | -0.034 (0.067) | 0.023 (0.067) | 0.064 (0.087) | 0.025 (0.070) | -0.065 (0.052) | -0.078 (0.070) | 0.022 (0.049) | 0.014 (0.050) | -0.036 (0.068) |
| Happy Questions | -0.027 (0.066) | -0.050 (0.066) | -0.079 (0.087) | -0.033 (0.069) | -0.010 (0.051) | -0.101 (0.068) | 0.011 (0.048) | 0.028 (0.049) | 0.058 (0.068) |
| Happy Writing | -0.008 (0.066) | 0.011 (0.066) | 0.014 (0.088) | 0.003 (0.069) | -0.034 (0.051) | -0.044 (0.069) | 0.039 (0.048) | 0.046 (0.049) | 0.003 (0.068) |
| Happiness | 0.049 (0.070) | 0.042 (0.069) | 0.215 (0.091) | 0.112 (0.073) | 0.007 (0.054) | -0.034 (0.072) | 0.135* (0.051) | 0.138* (0.052) | -0.051 (0.071) |
| Happy Photos ×  Happiness | 0.065 (0.099) | -0.038 (0.099) | -0.075 (0.130) | -0.084 (0.104) | 0.085 (0.078) | 0.082 (0.103) | -0.046 (0.072) | -0.019 (0.074) | 0.037 (0.102) |
| Happy Questions ×  Happiness | 0.067 (0.097) | 0.099 (0.097) | 0.176 (0.126) | 0.089 (0.102) | 0.045 (0.076) | 0.161 (0.101) | -0.041 (0.071) | -0.054 (0.072) | -0.137 (0.099) |
| Happy Writing ×  Happiness | 0.027 (0.097) | -0.019 (0.097) | -0.020 (0.129) | 0.038 (0.102) | 0.067 (0.076) | 0.104 (0.101) | -0.071 (0.071) | -0.062 (0.072) | -0.006 (0.100) |
| *N* | 1248 | 1248 | 930 | 1248 | 1248 | 1248 | 1248 | 1248 | 930 |
| *R*-squared | 0.006 | 0.004 | 0.036 | 0.020 | 0.008 | 0.008 | 0.013 | 0.014 | 0.013 |

Note. Cell entries are OLS regression coefficients with associated standard errors in parentheses. FT = feeling thermometer. * FDR adjusted *p* < .05.

**Table H.2.1** The effects of happiness treatments on affective polarization, feeling thermometers toward social groups, conspiracy endorsement, and deep fake recognition (Study 2, PL)

|  | Affective polarization | FT immigrants | FT feminists | FT neo-Nazis | FT nationalists | Conspiracy | Deep fake |
| --- | --- | --- | --- | --- | --- | --- | --- |
| Intercept | 0.347* (0.012) | 0.269* (0.020) | 0.337* (0.022) | 0.115* (0.012) | 0.403* (0.021) | 0.420* (0.016) | 0.242* (0.026) |
| Age | -0.114* (0.020) | 0.303* (0.034) | 0.347* (0.037) | -0.102* (0.020) | -0.304* (0.035) | -0.026 (0.026) | 0.132* (0.043) |
| Happy Photos | 0.009 (0.013) | -0.007 (0.022) | -0.025 (0.025) | 0.002 (0.013) | -0.006 (0.023) | -0.010 (0.017) | 0.037 (0.028) |
| Happy Questions | 0.026 (0.012) | -0.005 (0.021) | -0.013 (0.023) | 0.001 (0.012) | -0.001 (0.022) | -0.011 (0.016) | -0.013 (0.027) |
| Happy Writing | 0.006 (0.013) | 0.001 (0.022) | -0.041 (0.024) | 0.008 (0.013) | 0.011 (0.022) | 0.013 (0.017) | -0.010 (0.028) |
| *N* | 1109 | 1109 | 1109 | 1109 | 1109 | 1109 | 1109 |
| *R*-squared | 0.034 | 0.069 | 0.075 | 0.024 | 0.067 | 0.003 | 0.013 |

Note. Cell entries are OLS regression coefficients with associated standard errors in parentheses. FT = feeling thermometer. * FDR adjusted *p* < .05.

**Table H.2.2** The effects of happiness treatments on individual and subtractive affective polarization indicators (Study 2, PL)

|  | FT outparty | FT outparty supporters | FT out-gov. | FT out-ideologues | Social distance | Trait ratings | Outgroup trust | FT party (subt.) | FT party supporters (subt.) | FT gov. (subt.) | FT ideology (subt.) |
| --- | --- | --- | --- | --- | --- | --- | --- | --- | --- | --- | --- |
| Intercept | 0.208* (0.018) | 0.227* (0.018) | 0.301* (0.018) | 0.312* (0.022) | 0.559* (0.020) | 0.422* (0.015) | 0.380* (0.018) | 0.669* (0.016) | 0.687* (0.015) | 0.655* (0.016) | 0.643* (0.017) |
| Age | -0.136* (0.029) | -0.147* (0.030) | -0.195* (0.030) | -0.053 (0.036) | -0.060 (0.033) | -0.068* (0.025) | -0.124* (0.029) | 0.234* (0.027) | 0.218* (0.024) | 0.214* (0.026) | 0.156* (0.028) |
| Happy Photos | 0.001 (0.020) | 0.018 (0.020) | -0.004 (0.020) | 0.042 (0.024) | 0.004 (0.022) | 0.011 (0.016) | 0.006 (0.019) | -0.011 (0.018) | -0.008 (0.016) | 0.002 (0.018) | -0.007 (0.019) |
| Happy Questions | 0.007 (0.019) | 0.028 (0.019) | 0.032 (0.019) | 0.045 (0.023) | 0.014 (0.021) | 0.034 (0.015) | 0.032 (0.018) | -0.008 (0.017) | -0.018 (0.015) | -0.025 (0.017) | -0.018 (0.018) |
| Happy Writing | 0.011 (0.019) | 0.014 (0.020) | -0.011 (0.019) | 0.010 (0.023) | -0.012 (0.021) | 0.010 (0.016) | 0.022 (0.019) | -0.004 (0.017) | -0.001 (0.016) | 0.018 (0.017) | 0.007 (0.018) |
| *N* | 1109 | 1109 | 1109 | 800 | 1109 | 1109 | 1109 | 1109 | 1109 | 1109 | 800 |
| *R*-squared | 0.020 | 0.023 | 0.044 | 0.010 | 0.004 | 0.012 | 0.020 | 0.065 | 0.070 | 0.063 | 0.039 |

Note. Cell entries are OLS regression coefficients with associated standard errors in parentheses. FT = feeling thermometer. * FDR adjusted *p* < .05.

**Table H.2.3** The effects of interactions between happiness treatments and political identity strength on affective polarization, feeling thermometers toward social groups, conspiracy endorsement, and deep fake recognition (Study 2, PL)

|  | Affective polarization | FT immigrants | FT feminists | FT neo-Nazis | FT nationalists | Conspiracy | Deep fake |
| --- | --- | --- | --- | --- | --- | --- | --- |
| Intercept | 0.468* (0.024) | 0.259* (0.044) | 0.316* (0.049) | 0.144* (0.026) | 0.373* (0.046) | 0.416* (0.034) | 0.147* (0.057) |
| Age | -0.048* (0.019) | 0.309* (0.035) | 0.344* (0.039) | -0.096* (0.020) | -0.305* (0.036) | -0.026 (0.027) | 0.119* (0.044) |
| Happy Photos | 0.011 (0.037) | 0.069 (0.067) | 0.007 (0.075) | 0.051 (0.039) | 0.042 (0.069) | 0.043 (0.052) | 0.119 (0.086) |
| Happy Questions | 0.044 (0.033) | -0.028 (0.060) | -0.008 (0.067) | -0.064 (0.035) | 0.035 (0.062) | -0.040 (0.046) | 0.128 (0.077) |
| Happy Writing | 0.042 (0.035) | 0.049 (0.064) | -0.019 (0.071) | -0.030 (0.037) | 0.042 (0.066) | 0.018 (0.049) | 0.009 (0.082) |
| Pids | -0.241* (0.036) | 0.013 (0.066) | 0.035 (0.074) | -0.050 (0.039) | 0.050 (0.068) | 0.007 (0.051) | 0.163 (0.085) |
| Happy Photos ×  Pids | 0.005 (0.055) | -0.118 (0.101) | -0.051 (0.112) | -0.073 (0.059) | -0.077 (0.103) | -0.082 (0.077) | -0.136 (0.128) |
| Happy Questions ×  Pids | -0.024 (0.050) | 0.038 (0.091) | -0.009 (0.101) | 0.106 (0.053) | -0.058 (0.094) | 0.046 (0.070) | -0.230 (0.116) |
| Happy Writing ×  Pids | -0.049 (0.052) | -0.075 (0.096) | -0.034 (0.107) | 0.060 (0.056) | -0.049 (0.099) | -0.009 (0.074) | -0.036 (0.123) |
| *N* | 1109 | 1109 | 1109 | 1109 | 1109 | 1109 | 1109 |
| *R*-squared | 0.173 | 0.072 | 0.075 | 0.035 | 0.068 | 0.006 | 0.018 |

Note. Cell entries are OLS regression coefficients with associated standard errors in parentheses. FT = feeling thermometer. * FDR adjusted *p* < .05.

**Table H.2.4** The effects of interactions between happiness treatments and political identity strength on individual and subtractive affective polarization indicators (Study 2, PL)

|  | FT outparty | FT outparty supporters | FT out-gov. | FT out-ideologues | Social distance | Trait ratings | Outgroup trust | FT party (subt.) | FT party supporters (subt.) | FT gov. (subt.) | FT ideology (subt.) |
| --- | --- | --- | --- | --- | --- | --- | --- | --- | --- | --- | --- |
| Intercept | 0.232* (0.039) | 0.276* (0.040) | 0.495* (0.036) | 0.426* (0.046) | 0.738* (0.042) | 0.556* (0.031) | 0.527* (0.037) | 0.555* (0.034) | 0.569* (0.031) | 0.440* (0.029) | 0.486* (0.035) |
| Age | -0.106* (0.030) | -0.114* (0.031) | -0.081* (0.028) | 0.016 (0.036) | 0.021 (0.033) | 0.001 (0.024) | -0.049 (0.029) | 0.174* (0.027) | 0.158* (0.024) | 0.087* (0.023) | 0.073* (0.028) |
| Happy Photos | 0.027 (0.059) | 0.027 (0.061) | 0.079 (0.055) | -0.047 (0.070) | -0.013 (0.064) | 0.017 (0.047) | -0.005 (0.056) | -0.019 (0.052) | -0.012 (0.047) | -0.058 (0.045) | 0.074 (0.053) |
| Happy Questions | 0.046 (0.052) | 0.057 (0.054) | 0.090 (0.049) | 0.042 (0.062) | -0.002 (0.057) | 0.052 (0.042) | 0.046 (0.050) | 0.017 (0.046) | -0.009 (0.042) | -0.080 (0.040) | 0.005 (0.048) |
| Happy Writing | 0.096 (0.056) | 0.056 (0.057) | 0.004 (0.052) | 0.156 (0.068) | -0.027 (0.061) | 0.020 (0.044) | 0.050 (0.053) | -0.069 (0.049) | -0.035 (0.044) | -0.051 (0.042) | -0.084 (0.052) |
| Pids | -0.060 (0.058) | -0.102 (0.060) | -0.390* (0.054) | -0.226* (0.068) | -0.345* (0.063) | -0.263* (0.046) | -0.288* (0.055) | 0.225* (0.051) | 0.231* (0.046) | 0.434* (0.044) | 0.302* (0.052) |
| Happy Photos ×  Pids | -0.040 (0.088) | -0.010 (0.091) | -0.116 (0.081) | 0.143 (0.101) | 0.038 (0.096) | -0.001 (0.070) | 0.028 (0.084) | 0.004 (0.077) | -0.002 (0.070) | 0.078 (0.067) | -0.135 (0.077) |
| Happy Questions ×  Pids | -0.061 (0.079) | -0.043 (0.082) | -0.083 (0.074) | 0.011 (0.091) | 0.033 (0.087) | -0.023 (0.063) | -0.017 (0.076) | -0.044 (0.070) | -0.018 (0.063) | 0.079 (0.060) | -0.043 (0.070) |
| Happy Writing ×  Pids | -0.133 (0.084) | -0.063 (0.086) | -0.012 (0.078) | -0.211 (0.098) | 0.034 (0.092) | -0.009 (0.067) | -0.034 (0.080) | 0.096 (0.074) | 0.046 (0.067) | 0.095 (0.064) | 0.124 (0.075) |
| *N* | 1109 | 1109 | 1109 | 800 | 1109 | 1109 | 1109 | 1109 | 1109 | 1109 | 800 |
| *R*-squared | 0.035 | 0.039 | 0.217 | 0.078 | 0.080 | 0.112 | 0.104 | 0.128 | 0.143 | 0.336 | 0.170 |

Note. Cell entries are OLS regression coefficients with associated standard errors in parentheses. FT = feeling thermometer. * FDR adjusted *p* < .05.

**Table H.2.5** The effects of interactions between happiness treatments and political interest on affective polarization, feeling thermometers toward social groups, conspiracy endorsement, and deep fake recognition (Study 2, PL)

|  | Affective polarization | FT immigrants | FT feminists | FT neo-Nazis | FT nationalists | Conspiracy | Deep fake |
| --- | --- | --- | --- | --- | --- | --- | --- |
| Intercept | 0.367* (0.022) | 0.300* (0.038) | 0.371* (0.042) | 0.133* (0.022) | 0.379* (0.039) | 0.432* (0.029) | 0.243* (0.048) |
| Age | -0.084* (0.020) | 0.278* (0.035) | 0.350* (0.039) | -0.085* (0.020) | -0.311* (0.036) | -0.007 (0.027) | 0.175* (0.045) |
| Happy Photos | 0.030 (0.033) | -0.061 (0.056) | 0.004 (0.062) | 0.052 (0.033) | 0.018 (0.058) | 0.036 (0.043) | 0.155 (0.071) |
| Happy Questions | 0.058 (0.030) | -0.125 (0.052) | -0.113 (0.058) | -0.038 (0.030) | 0.016 (0.054) | 0.014 (0.040) | 0.015 (0.066) |
| Happy Writing | 0.053 (0.032) | -0.088 (0.055) | -0.086 (0.061) | 0.030 (0.032) | 0.021 (0.057) | 0.008 (0.042) | 0.091 (0.070) |
| Interest | -0.058 (0.034) | -0.037 (0.058) | -0.063 (0.065) | -0.043 (0.034) | 0.047 (0.060) | -0.035 (0.045) | -0.031 (0.074) |
| Happy Photos ×  Interest | -0.035 (0.051) | 0.092 (0.087) | -0.045 (0.097) | -0.081 (0.051) | -0.043 (0.090) | -0.077 (0.067) | -0.198 (0.112) |
| Happy Questions ×  Interest | -0.051 (0.048) | 0.204* (0.082) | 0.172 (0.091) | 0.069 (0.048) | -0.030 (0.084) | -0.040 (0.063) | -0.047 (0.104) |
| Happy Writing ×  Interest | -0.074 (0.050) | 0.150 (0.085) | 0.078 (0.095) | -0.034 (0.050) | -0.019 (0.088) | 0.009 (0.066) | -0.164 (0.109) |
| *N* | 1109 | 1109 | 1109 | 1109 | 1109 | 1109 | 1109 |
| *R*-squared | 0.060 | 0.079 | 0.080 | 0.039 | 0.068 | 0.011 | 0.025 |

Note. Cell entries are OLS regression coefficients with associated standard errors in parentheses. FT = feeling thermometer. * FDR adjusted *p* < .05.

**Table H.2.6** The effects of interactions between happiness treatments and political interest on individual and subtractive affective polarization indicators (Study 1, US)

|  | FT outparty | FT outparty supporters | FT out-gov. | FT out-ideologues | Social distance | Trait ratings | Outgroup trust | FT party (subt.) | FT party supporters (subt.) | FT gov. (subt.) | FT ideology (subt.) |
| --- | --- | --- | --- | --- | --- | --- | --- | --- | --- | --- | --- |
| Intercept | 0.231* (0.033) | 0.270* (0.034) | 0.319* (0.034) | 0.370* (0.044) | 0.571* (0.037) | 0.429* (0.028) | 0.384* (0.033) | 0.574* (0.029) | 0.598* (0.027) | 0.621* (0.029) | 0.539* (0.034) |
| Age | -0.109* (0.031) | -0.128* (0.032) | -0.163* (0.031) | -0.016 (0.037) | -0.014 (0.034) | -0.040 (0.025) | -0.093* (0.030) | 0.168* (0.027) | 0.163* (0.025) | 0.166* (0.027) | 0.095* (0.029) |
| Happy Photos | 0.022 (0.049) | -0.008 (0.051) | 0.030 (0.050) | 0.050 (0.068) | 0.065 (0.055) | 0.037 (0.041) | 0.052 (0.049) | -0.037 (0.043) | -0.014 (0.040) | -0.046 (0.043) | -0.005 (0.052) |
| Happy Questions | 0.030 (0.046) | 0.022 (0.047) | 0.096 (0.046) | -0.011 (0.061) | 0.078 (0.051) | 0.101* (0.038) | 0.066 (0.045) | -0.019 (0.040) | -0.015 (0.037) | -0.087 (0.040) | 0.022 (0.047) |
| Happy Writing | 0.039 (0.048) | -0.002 (0.050) | 0.019 (0.049) | 0.061 (0.063) | 0.087 (0.054) | 0.061 (0.040) | 0.112 (0.048) | 0.001 (0.042) | 0.008 (0.039) | -0.035 (0.043) | -0.032 (0.049) |
| Interest | -0.060 (0.051) | -0.089 (0.053) | -0.054 (0.052) | -0.120 (0.065) | -0.053 (0.057) | -0.033 (0.042) | -0.027 (0.050) | 0.213* (0.045) | 0.195* (0.041) | 0.094 (0.045) | 0.209* (0.050) |
| Happy Photos ×  Interest | -0.034 (0.077) | 0.046 (0.079) | -0.056 (0.078) | -0.006 (0.099) | -0.102 (0.086) | -0.044 (0.064) | -0.077 (0.076) | 0.036 (0.067) | 0.004 (0.062) | 0.079 (0.068) | -0.014 (0.077) |
| Happy Questions ×  Interest | -0.035 (0.072) | 0.015 (0.074) | -0.105 (0.073) | 0.092 (0.091) | -0.105 (0.081) | -0.111 (0.060) | -0.055 (0.071) | 0.010 (0.063) | -0.013 (0.058) | 0.100 (0.063) | -0.070 (0.070) |
| Happy Writing ×  Interest | -0.042 (0.075) | 0.032 (0.077) | -0.046 (0.076) | -0.074 (0.093) | -0.160 (0.084) | -0.082 (0.062) | -0.145 (0.074) | -0.021 (0.066) | -0.027 (0.060) | 0.080 (0.066) | 0.050 (0.072) |
| *N* | 1109 | 1109 | 1109 | 800 | 1109 | 1109 | 1109 | 1109 | 1109 | 1109 | 800 |
| *R*-squared | 0.029 | 0.028 | 0.058 | 0.027 | 0.026 | 0.030 | 0.034 | 0.130 | 0.126 | 0.099 | 0.104 |

Note. Cell entries are OLS regression coefficients with associated standard errors in parentheses. FT = feeling thermometer. * FDR adjusted *p* < .05.

**Table H.2.7** The effects of interactions between happiness treatments and attitude toward the government on affective polarization, feeling thermometers toward social groups, conspiracy endorsement, and deep fake recognition (Study 2, PL)

|  | Affective polarization | FT immigrants | FT feminists | FT neo-Nazis | FT nationalists | Conspiracy | Deep fake |
| --- | --- | --- | --- | --- | --- | --- | --- |
| Intercept | 0.309* (0.013) | 0.318* (0.023) | 0.395* (0.025) | 0.092* (0.014) | 0.309* (0.022) | 0.367* (0.017) | 0.183* (0.030) |
| Age | -0.107* (0.019) | 0.293* (0.033) | 0.340* (0.036) | -0.097* (0.020) | -0.290* (0.031) | -0.018 (0.025) | 0.145* (0.043) |
| Happy Photos | 0.021 (0.016) | -0.011 (0.027) | 0.007 (0.029) | 0.021 (0.016) | -0.002 (0.026) | 0.012 (0.021) | 0.097* (0.035) |
| Happy Questions | 0.011 (0.015) | 0.008 (0.026) | -0.012 (0.029) | 0.001 (0.016) | 0.003 (0.026) | -0.002 (0.020) | 0.001 (0.035) |
| Happy Writing | 0.014 (0.016) | 0.019 (0.027) | -0.021 (0.029) | 0.009 (0.016) | 0.001 (0.026) | 0.026 (0.021) | 0.009 (0.035) |
| Att. Gov. (Supporter) | 0.099* (0.018) | -0.127* (0.030) | -0.156* (0.033) | 0.059* (0.018) | 0.247* (0.029) | 0.139* (0.023) | 0.152* (0.040) |
| Happy Photos ×  Att. Gov.  (Supporter) | -0.034 (0.026) | 0.007 (0.045) | -0.094 (0.049) | -0.053 (0.027) | -0.008 (0.044) | -0.063 (0.035) | -0.172* (0.059) |
| Happy Questions ×  Att. Gov.  (Supporter) | 0.014 (0.024) | -0.005 (0.042) | 0.027 (0.046) | -0.011 (0.025) | -0.055 (0.040) | -0.047 (0.032) | -0.061 (0.055) |
| Happy Writing ×  Att. Gov.  (Supporter) | -0.035 (0.025) | -0.025 (0.043) | -0.027 (0.047) | -0.011 (0.026) | -0.010 (0.042) | -0.051 (0.033) | -0.066 (0.056) |
| *N* | 1109 | 1109 | 1109 | 1109 | 1109 | 1109 | 1109 |
| *R*-squared | 0.115 | 0.129 | 0.163 | 0.046 | 0.233 | 0.067 | 0.035 |

Note. Cell entries are OLS regression coefficients with associated standard errors in parentheses. FT = feeling thermometer. * FDR adjusted *p* < .05.

**Table H.2.8** The effects of interactions between happiness treatments and attitude toward the government on individual and subtractive affective polarization indicators (Study 2, PL)

|  | FT outparty | FT outparty supporters | FT out-gov. | FT out-ideologues | Social distance | Trait ratings | Outgroup trust | FT party (subt.) | FT party supporters (subt.) | FT gov. (subt.) | FT ideology (subt.) |
| --- | --- | --- | --- | --- | --- | --- | --- | --- | --- | --- | --- |
| Intercept | 0.184* (0.020) | 0.204* (0.021) | 0.235* (0.020) | 0.276* (0.026) | 0.508* (0.023) | 0.388* (0.017) | 0.347* (0.020) | 0.683* (0.018) | 0.704* (0.017) | 0.692* (0.018) | 0.653* (0.020) |
| Age | -0.134* (0.029) | -0.145* (0.030) | -0.185* (0.029) | -0.048 (0.036) | -0.047 (0.033) | -0.062* (0.024) | -0.117* (0.029) | 0.232* (0.027) | 0.215* (0.024) | 0.206* (0.026) | 0.154* (0.029) |
| Happy Photos | 0.002 (0.024) | 0.014 (0.025) | 0.017 (0.023) | 0.066 (0.030) | 0.030 (0.027) | 0.019 (0.020) | 0.024 (0.024) | -0.010 (0.022) | -0.013 (0.020) | -0.022 (0.021) | -0.023 (0.024) |
| Happy Questions | -0.010 (0.024) | -0.002 (0.024) | 0.017 (0.023) | 0.054 (0.030) | -0.003 (0.026) | 0.018 (0.020) | 0.024 (0.023) | 0.005 (0.022) | 0.001 (0.020) | -0.013 (0.021) | -0.022 (0.024) |
| Happy Writing | 0.033 (0.024) | 0.028 (0.025) | 0.005 (0.023) | 0.038 (0.030) | -0.024 (0.027) | 0.005 (0.020) | 0.026 (0.024) | -0.015 (0.022) | -0.014 (0.020) | 0.011 (0.021) | -0.001 (0.024) |
| Att. Gov.  (Supporter) | 0.067 (0.027) | 0.060 (0.028) | 0.175* (0.026) | 0.089* (0.033) | 0.130* (0.030) | 0.087* (0.022) | 0.086* (0.027) | -0.037 (0.025) | -0.046 (0.023) | -0.094* (0.024) | -0.023 (0.026) |
| Happy Photos ×  Att. Gov.  (Supporter) | -0.002 (0.041) | 0.013 (0.042) | -0.058 (0.040) | -0.063 (0.049) | -0.073 (0.045) | -0.022 (0.033) | -0.050 (0.040) | -0.005 (0.037) | 0.015 (0.034) | 0.068 (0.036) | 0.043 (0.039) |
| Happy Questions ×  Att. Gov.  (Supporter) | 0.027 (0.038) | 0.057 (0.039) | 0.001 (0.037) | -0.039 (0.046) | 0.014 (0.042) | 0.020 (0.031) | 0.002 (0.037) | -0.023 (0.034) | -0.033 (0.031) | -0.011 (0.034) | 0.014 (0.036) |
| Happy Writing ×  Att. Gov.  (Supporter) | -0.063 (0.039) | -0.041 (0.040) | -0.064 (0.038) | -0.078 (0.046) | 0.010 (0.043) | -0.001 (0.032) | -0.021 (0.038) | 0.033 (0.035) | 0.036 (0.032) | 0.028 (0.034) | 0.021 (0.037) |
| *N* | 1109 | 1109 | 1109 | 800 | 1109 | 1109 | 1109 | 1109 | 1109 | 1109 | 800 |
| *R*-squared | 0.040 | 0.049 | 0.141 | 0.023 | 0.061 | 0.064 | 0.045 | 0.074 | 0.086 | 0.099 | 0.041 |

Note. Cell entries are OLS regression coefficients with associated standard errors in parentheses. FT = feeling thermometer. * FDR adjusted *p* < .05.

**Table H.2.9** The effects of interactions between happiness treatments and ideology on affective polarization, feeling thermometers toward social groups, conspiracy endorsement, and deep fake recognition (Study 2, PL)

|  | Affective polarization | FT immigrants | FT feminists | FT neo-Nazis | FT nationalists | Conspiracy | Deep fake |
| --- | --- | --- | --- | --- | --- | --- | --- |
| Intercept | 0.307* (0.020) | 0.416* (0.034) | 0.536* (0.036) | 0.079* (0.020) | 0.185* (0.033) | 0.310* (0.026) | 0.113* (0.045) |
| Age | -0.102* (0.020) | 0.267* (0.033) | 0.297* (0.035) | -0.087* (0.019) | -0.249* (0.032) | 0.001 (0.025) | 0.155* (0.043) |
| Happy Photos | 0.012 (0.026) | -0.024 (0.044) | 0.013 (0.047) | -0.002 (0.026) | -0.001 (0.042) | 0.008 (0.034) | 0.159* (0.058) |
| Happy Questions | 0.004 (0.026) | -0.010 (0.044) | -0.001 (0.047) | -0.032 (0.026) | 0.020 (0.042) | -0.031 (0.034) | 0.048 (0.058) |
| Happy Writing | -0.006 (0.026) | 0.008 (0.043) | -0.027 (0.047) | -0.036 (0.026) | -0.041 (0.042) | 0.015 (0.033) | 0.037 (0.057) |
| Ideology | 0.066 (0.031) | -0.256* (0.051) | -0.345* (0.055) | 0.057 (0.031) | 0.377* (0.050) | 0.190* (0.039) | 0.232* (0.068) |
| Happy Photos ×  Ideology | -0.006 (0.045) | 0.030 (0.074) | -0.078 (0.079) | 0.008 (0.044) | -0.007 (0.072) | -0.035 (0.057) | -0.238 (0.098) |
| Happy Questions ×  Ideology | 0.037 (0.044) | 0.024 (0.072) | -0.001 (0.077) | 0.057 (0.043) | -0.060 (0.070) | 0.026 (0.055) | -0.125 (0.095) |
| Happy Writing ×  Ideology | 0.019 (0.044) | -0.004 (0.072) | -0.014 (0.078) | 0.080 (0.043) | 0.087 (0.070) | -0.011 (0.056) | -0.094 (0.096) |
| *N* | 1109 | 1109 | 1109 | 1109 | 1109 | 1109 | 1109 |
| *R*-squared | 0.057 | 0.138 | 0.202 | 0.060 | 0.231 | 0.077 | 0.029 |

Note. Cell entries are OLS regression coefficients with associated standard errors in parentheses. FT = feeling thermometer. * FDR adjusted *p* < .05.

**Table H.2.10** The effects of interactions between happiness treatments and ideology on individual and subtractive affective polarization indicators (Study 2, PL)

|  | FT outparty | FT outparty supporters | FT out-gov. | FT out-ideologues | Social distance | Trait ratings | Outgroup trust | FT party (subt.) | FT party supporters (subt.) | FT gov. (subt.) | FT ideology (subt.) |
| --- | --- | --- | --- | --- | --- | --- | --- | --- | --- | --- | --- |
| Intercept | 0.204* (0.031) | 0.232* (0.032) | 0.214* (0.031) | 0.279* (0.035) | 0.493* (0.035) | 0.362* (0.026) | 0.346* (0.030) | 0.656* (0.028) | 0.667* (0.025) | 0.680* (0.028) | 0.655* (0.028) |
| Age | -0.129* (0.030) | -0.138* (0.031) | -0.176* (0.030) | -0.042 (0.036) | -0.041 (0.033) | -0.054 (0.025) | -0.115* (0.029) | 0.234* (0.027) | 0.217* (0.025) | 0.207* (0.027) | 0.152* (0.029) |
| Happy Photos | -0.032 (0.040) | -0.017 (0.041) | 0.048 (0.040) | 0.050 (0.043) | 0.018 (0.045) | 0.021 (0.033) | 0.016 (0.039) | 0.026 (0.036) | 0.035 (0.033) | -0.029 (0.036) | -0.003 (0.034) |
| Happy Questions | -0.049 (0.040) | -0.066 (0.041) | 0.034 (0.040) | 0.040 (0.044) | -0.004 (0.045) | 0.056 (0.033) | 0.027 (0.039) | 0.020 (0.036) | 0.042 (0.033) | -0.011 (0.036) | -0.028 (0.035) |
| Happy Writing | -0.017 (0.039) | -0.031 (0.041) | 0.007 (0.040) | 0.013 (0.043) | -0.028 (0.044) | 0.000 (0.033) | 0.019 (0.039) | 0.006 (0.036) | 0.016 (0.033) | 0.018 (0.036) | 0.016 (0.034) |
| Ideology | 0.002 (0.047) | -0.017 (0.048) | 0.153* (0.047) | 0.054 (0.048) | 0.112 (0.053) | 0.106* (0.039) | 0.059 (0.046) | 0.025 (0.042) | 0.038 (0.039) | -0.043 (0.042) | -0.019 (0.038) |
| Happy Photos ×  Ideology | 0.065 (0.067) | 0.067 (0.070) | -0.102 (0.068) | -0.014 (0.069) | -0.028 (0.076) | -0.019 (0.056) | -0.019 (0.067) | -0.072 (0.061) | -0.084 (0.056) | 0.060 (0.061) | -0.008 (0.055) |
| Happy Questions ×  Ideology | 0.103 (0.065) | 0.171* (0.067) | -0.011 (0.066) | 0.004 (0.068) | 0.027 (0.074) | -0.044 (0.054) | 0.007 (0.065) | -0.052 (0.059) | -0.110 (0.054) | -0.024 (0.059) | 0.018 (0.054) |
| Happy Writing ×  Ideology | 0.052 (0.066) | 0.085 (0.068) | -0.039 (0.066) | -0.009 (0.068) | 0.026 (0.074) | 0.015 (0.055) | 0.005 (0.065) | -0.019 (0.060) | -0.034 (0.054) | 0.001 (0.059) | -0.015 (0.054) |
| *N* | 1109 | 1109 | 1109 | 800 | 1109 | 1109 | 1109 | 1109 | 1109 | 1109 | 800 |
| *R*-squared | 0.027 | 0.035 | 0.067 | 0.015 | 0.023 | 0.033 | 0.026 | 0.067 | 0.075 | 0.067 | 0.041 |

Note. Cell entries are OLS regression coefficients with associated standard errors in parentheses. FT = feeling thermometer. * FDR adjusted *p* < .05.

**Table H.2.11** The effects of interactions between happiness treatments and happiness strength on affective polarization, feeling thermometers toward social groups, conspiracy endorsement, and deep fake recognition (Study 2, PL)

|  | Affective polarization | FT immigrants | FT feminists | FT neo-Nazis | FT nationalists | Conspiracy | Deep fake |
| --- | --- | --- | --- | --- | --- | --- | --- |
| Intercept | 0.206* (0.032) | 0.275* (0.056) | 0.341* (0.062) | 0.054 (0.032) | 0.175* (0.056) | 0.266* (0.042) | 0.109 (0.071) |
| Age | -0.121* (0.020) | 0.304* (0.034) | 0.346* (0.037) | -0.104* (0.020) | -0.316* (0.034) | -0.034 (0.025) | 0.128* (0.043) |
| Happy Photos | 0.120* (0.047) | 0.039 (0.081) | 0.038 (0.090) | 0.047 (0.047) | 0.104 (0.082) | 0.103 (0.061) | 0.122 (0.104) |
| Happy Questions | 0.112* (0.045) | 0.025 (0.077) | 0.016 (0.086) | 0.007 (0.045) | 0.062 (0.078) | -0.001 (0.058) | 0.075 (0.099) |
| Happy Writing | 0.071 (0.046) | 0.009 (0.079) | -0.093 (0.088) | 0.057 (0.046) | 0.135 (0.080) | 0.050 (0.060) | 0.159 (0.101) |
| Happiness | 0.255* (0.054) | -0.011 (0.093) | -0.007 (0.104) | 0.110 (0.055) | 0.415* (0.095) | 0.280* (0.071) | 0.242 (0.119) |
| Happy Photos ×  Happiness | -0.197 (0.081) | -0.084 (0.140) | -0.113 (0.155) | -0.079 (0.081) | -0.194 (0.141) | -0.202 (0.105) | -0.151 (0.178) |
| Happy Questions ×  Happiness | -0.156 (0.076) | -0.050 (0.131) | -0.051 (0.145) | -0.013 (0.076) | -0.121 (0.132) | -0.025 (0.099) | -0.160 (0.167) |
| Happy Writing ×  Happiness | -0.119 (0.078) | -0.013 (0.134) | 0.091 (0.148) | -0.089 (0.078) | -0.225 (0.135) | -0.072 (0.101) | -0.300 (0.171) |
| *N* | 1109 | 1109 | 1109 | 1109 | 1109 | 1109 | 1109 |
| *R*-squared | 0.062 | 0.070 | 0.076 | 0.031 | 0.098 | 0.037 | 0.017 |

Note. Cell entries are OLS regression coefficients with associated standard errors in parentheses. FT = feeling thermometer. * FDR adjusted *p* < .05.

**Table H.2.12.** The effects of interactions between happiness treatments and happiness strength on individual and subtractive affective polarization indicators (Study 2, PL)

|  | FT outparty | FT outparty supporters | FT out-gov. | FT out-ideologues | Social distance | Trait ratings | Outgroup trust | FT party (subt.) | FT party supporters (subt.) | FT gov. (subt.) | FT ideology (subt.) |
| --- | --- | --- | --- | --- | --- | --- | --- | --- | --- | --- | --- |
| Intercept | 0.105 (0.049) | 0.116 (0.050) | 0.139* (0.049) | 0.212* (0.060) | 0.360* (0.055) | 0.294* (0.040) | 0.188* (0.048) | 0.645* (0.044) | 0.697* (0.040) | 0.763* (0.044) | 0.612* (0.048) |
| Age | -0.142* (0.030) | -0.153* (0.030) | -0.204* (0.030) | -0.059 (0.036) | -0.070 (0.033) | -0.075* (0.024) | -0.132* (0.029) | 0.232* (0.027) | 0.217* (0.024) | 0.218* (0.027) | 0.156* (0.029) |
| Happy Photos | 0.079 (0.071) | 0.134 (0.073) | 0.160 (0.072) | 0.075 (0.086) | 0.118 (0.080) | 0.110 (0.059) | 0.174* (0.070) | -0.031 (0.064) | -0.084 (0.059) | -0.142 (0.064) | 0.060 (0.069) |
| Happy Questions | 0.103 (0.068) | 0.084 (0.070) | 0.141 (0.068) | 0.122 (0.081) | 0.150 (0.076) | 0.076 (0.056) | 0.170* (0.066) | -0.010 (0.061) | -0.034 (0.056) | -0.155* (0.061) | -0.024 (0.064) |
| Happy Writing | 0.047 (0.069) | 0.048 (0.071) | 0.042 (0.070) | 0.073 (0.083) | 0.108 (0.078) | 0.054 (0.057) | 0.155 (0.068) | 0.026 (0.063) | -0.022 (0.057) | -0.073 (0.062) | 0.035 (0.066) |
| Happiness | 0.187 (0.082) | 0.202 (0.084) | 0.295* (0.083) | 0.182 (0.100) | 0.362* (0.092) | 0.232* (0.068) | 0.349* (0.080) | 0.044 (0.074) | -0.018 (0.068) | -0.196* (0.073) | 0.054 (0.080) |
| Happy Photos ×  Happiness | -0.140 (0.122) | -0.206 (0.126) | -0.292 (0.124) | -0.060 (0.146) | -0.203 (0.137) | -0.175 (0.101) | -0.298* (0.120) | 0.036 (0.111) | 0.137 (0.101) | 0.257 (0.110) | -0.120 (0.116) |
| Happy Questions ×  Happiness | -0.171 (0.114) | -0.102 (0.118) | -0.196 (0.116) | -0.138 (0.136) | -0.244 (0.129) | -0.078 (0.095) | -0.248 (0.113) | 0.002 (0.104) | 0.029 (0.095) | 0.231 (0.103) | 0.007 (0.108) |
| Happy Writing ×  Happiness | -0.068 (0.117) | -0.064 (0.121) | -0.099 (0.118) | -0.115 (0.139) | -0.218 (0.132) | -0.082 (0.097) | -0.239 (0.115) | -0.053 (0.106) | 0.036 (0.097) | 0.162 (0.105) | -0.050 (0.111) |
| *N* | 1109 | 1109 | 1109 | 800 | 1109 | 1109 | 1109 | 1109 | 1109 | 1109 | 800 |
| *R*-squared | 0.026 | 0.032 | 0.061 | 0.017 | 0.024 | 0.032 | 0.040 | 0.067 | 0.072 | 0.070 | 0.041 |

Note. Cell entries are OLS regression coefficients with associated standard errors in parentheses. FT = feeling thermometer. * FDR adjusted *p* < .05.

**Table H.3.1** The effects of happiness treatments on affective polarization, feeling thermometers toward social groups, conspiracy endorsement, and deep fake recognition (Study 3, NL)

|  | Affective polarization | FT immigrants | FT feminists | FT nationalists | Conspiracy | Deep fake |
| --- | --- | --- | --- | --- | --- | --- |
| Intercept | 0.381* (0.005) | 0.532* (0.008) | 0.534* (0.009) | 0.437* (0.009) | 0.353* (0.009) | 0.109* (0.008) |
| Happy Photos | -0.005 (0.010) | 0.012 (0.017) | 0.005 (0.018) | 0.015 (0.018) | 0.006 (0.018) | -0.018 (0.016) |
| Happy Questions | 0.002 (0.010) | 0.016 (0.017) | 0.016 (0.018) | 0.001 (0.018) | 0.015 (0.018) | 0.026 (0.016) |
| Happy Writing | 0.010 (0.010) | 0.049* (0.018) | 0.028 (0.019) | 0.000 (0.019) | -0.025 (0.019) | -0.014 (0.017) |
| *N* | 1254 | 1254 | 1254 | 1254 | 1254 | 1254 |
| *R*-squared | 0.001 | 0.006 | 0.002 | 0.001 | 0.003 | 0.005 |

Note. Cell entries are OLS regression coefficients with associated standard errors in parentheses. FT = feeling thermometer. * FDR adjusted *p* < .05.

**Table H.3.2** The effects of happiness treatments on individual and subtractive affective polarization indicators (Study 3, NL)

|  | FT outparty | FT outparty supporters | FT out-ideologues | Social distance | Trait ratings | Outgroup trust | FT party (subt.) | FT party supporters (subt.) | FT ideology (subt.) |
| --- | --- | --- | --- | --- | --- | --- | --- | --- | --- |
| Intercept | 0.145* (0.008) | 0.189* (0.008) | 0.375* (0.007) | 0.585* (0.008) | 0.491* (0.005) | 0.502* (0.007) | 0.808* (0.006) | 0.771* (0.006) | 0.611* (0.006) |
| Happy Photos | 0.000 (0.015) | -0.020 (0.016) | -0.006 (0.015) | 0.000 (0.016) | -0.005 (0.011) | -0.002 (0.015) | 0.004 (0.011) | 0.009 (0.012) | 0.012 (0.012) |
| Happy Questions | 0.006 (0.016) | 0.004 (0.016) | 0.001 (0.015) | 0.023 (0.016) | -0.009 (0.011) | -0.015 (0.015) | -0.009 (0.011) | -0.010 (0.012) | -0.004 (0.013) |
| Happy Writing | 0.017 (0.016) | 0.005 (0.017) | 0.005 (0.016) | 0.010 (0.017) | 0.002 (0.011) | 0.018 (0.016) | -0.008 (0.012) | -0.005 (0.012) | -0.004 (0.013) |
| *N* | 1254 | 1254 | 1254 | 1254 | 1254 | 1254 | 1254 | 1254 | 1254 |
| *R*-squared | 0.001 | 0.002 | 0.000 | 0.002 | 0.001 | 0.002 | 0.001 | 0.001 | 0.001 |

Note. Cell entries are OLS regression coefficients with associated standard errors in parentheses. FT = feeling thermometer. * FDR adjusted *p* < .05.

**Table H.3.3** The effects of interactions between happiness treatments and political identity strength on affective polarization, feeling thermometers toward social groups, conspiracy endorsement, and deep fake recognition (Study 3, NL)

|  | Affective polarization | FT immigrants | FT feminists | FT nationalists | Conspiracy | Deep fake |
| --- | --- | --- | --- | --- | --- | --- |
| Intercept | 0.488* (0.014) | 0.594* (0.025) | 0.511* (0.027) | 0.377* (0.026) | 0.281* (0.027) | 0.100* (0.025) |
| Happy Photos | -0.082* (0.029) | -0.032 (0.052) | -0.033 (0.055) | -0.085 (0.054) | -0.065 (0.055) | -0.003 (0.050) |
| Happy Questions | -0.024 (0.028) | -0.006 (0.050) | -0.012 (0.053) | -0.077 (0.052) | -0.024 (0.052) | -0.080 (0.048) |
| Happy Writing | -0.007 (0.031) | -0.053 (0.055) | -0.031 (0.059) | 0.130 (0.058) | -0.043 (0.059) | 0.013 (0.054) |
| Pids | -0.201* (0.025) | -0.116* (0.045) | 0.043 (0.048) | 0.112 (0.047) | 0.137* (0.047) | 0.017 (0.044) |
| Happy Photos ×  Pids | 0.145* (0.052) | 0.083 (0.091) | 0.070 (0.097) | 0.185 (0.096) | 0.131 (0.096) | -0.029 (0.089) |
| Happy Questions ×  Pids | 0.051 (0.049) | 0.043 (0.087) | 0.051 (0.093) | 0.143 (0.091) | 0.070 (0.092) | 0.197 (0.084) |
| Happy Writing ×  Pids | 0.035 (0.055) | 0.191 (0.098) | 0.109 (0.104) | -0.242 (0.102) | 0.030 (0.103) | -0.051 (0.095) |
| *N* | 1254 | 1254 | 1254 | 1254 | 1254 | 1254 |
| *R*-squared | 0.067 | 0.013 | 0.007 | 0.025 | 0.025 | 0.012 |

Note. Cell entries are OLS regression coefficients with associated standard errors in parentheses. FT = feeling thermometer. * FDR adjusted *p* < .05.

**Table H.3.4** The effects of interactions between happiness treatments and political identity strength on individual and subtractive affective polarization indicators (Study 3, NL)

|  | FT outparty | FT outparty supporters | FT out-ideologues | Social distance | Trait ratings | Outgroup trust | FT party (subt.) | FT party supporters (subt.) | FT ideology (subt.) |
| --- | --- | --- | --- | --- | --- | --- | --- | --- | --- |
| Intercept | 0.210* (0.023) | 0.270* (0.024) | 0.543* (0.022) | 0.678* (0.023) | 0.592* (0.016) | 0.635* (0.022) | 0.716* (0.017) | 0.662* (0.017) | 0.397* (0.017) |
| Happy Photos | -0.051 (0.048) | -0.135* (0.049) | -0.104 (0.044) | -0.023 (0.048) | -0.056 (0.033) | -0.121* (0.046) | -0.007 (0.034) | 0.040 (0.035) | 0.039 (0.034) |
| Happy Questions | -0.107 (0.046) | -0.075 (0.047) | 0.031 (0.043) | 0.051 (0.046) | -0.004 (0.032) | -0.043 (0.044) | 0.037 (0.033) | 0.020 (0.034) | -0.050 (0.033) |
| Happy Writing | 0.024 (0.051) | 0.010 (0.053) | -0.019 (0.048) | -0.004 (0.052) | -0.012 (0.035) | -0.042 (0.049) | -0.027 (0.037) | -0.010 (0.038) | 0.010 (0.037) |
| Pids | -0.122* (0.041) | -0.152* (0.043) | -0.315* (0.039) | -0.176* (0.042) | -0.190* (0.029) | -0.251* (0.039) | 0.173* (0.030) | 0.207* (0.031) | 0.403* (0.030) |
| Happy Photos ×  Pids | 0.097 (0.084) | 0.216 (0.087) | 0.186 (0.078) | 0.045 (0.085) | 0.098 (0.058) | 0.225* (0.080) | 0.018 (0.061) | -0.061 (0.062) | -0.056 (0.060) |
| Happy Questions ×  Pids | 0.210* (0.080) | 0.149 (0.083) | -0.052 (0.075) | -0.050 (0.081) | -0.006 (0.055) | 0.055 (0.077) | -0.087 (0.058) | -0.057 (0.059) | 0.081 (0.057) |
| Happy Writing ×  Pids | -0.010 (0.090) | -0.005 (0.093) | 0.052 (0.084) | 0.030 (0.091) | 0.030 (0.062) | 0.115 (0.086) | 0.030 (0.065) | 0.005 (0.066) | -0.034 (0.064) |
| *N* | 1254 | 1254 | 1254 | 1254 | 1254 | 1254 | 1254 | 1254 | 1254 |
| *R*-squared | 0.011 | 0.015 | 0.085 | 0.029 | 0.056 | 0.043 | 0.049 | 0.058 | 0.228 |

Note. Cell entries are OLS regression coefficients with associated standard errors in parentheses. FT = feeling thermometer. * FDR adjusted *p* < .05.

**Table H.3.5** The effects of interactions between happiness treatments and political interest on affective polarization, feeling thermometers toward social groups, conspiracy endorsement, and deep fake recognition (Study 3, NL)

|  | Affective polarization | FT immigrants | FT feminists | FT nationalists | Conspiracy | Deep fake |
| --- | --- | --- | --- | --- | --- | --- |
| Intercept | 0.391* (0.012) | 0.474* (0.020) | 0.498* (0.022) | 0.482* (0.022) | 0.430* (0.022) | 0.100* (0.020) |
| Happy Photos | 0.014 (0.024) | 0.065 (0.041) | 0.058 (0.044) | -0.024 (0.043) | -0.048 (0.043) | -0.058 (0.040) |
| Happy Questions | 0.028 (0.026) | 0.018 (0.044) | -0.023 (0.047) | 0.024 (0.046) | 0.018 (0.046) | 0.031 (0.043) |
| Happy Writing | -0.021 (0.026) | 0.094 (0.044) | 0.041 (0.047) | -0.017 (0.047) | -0.098 (0.047) | -0.006 (0.043) |
| Interest | -0.016 (0.019) | 0.103* (0.033) | 0.063 (0.035) | -0.079 (0.035) | -0.137* (0.035) | 0.016 (0.032) |
| Happy Photos ×  Interest | -0.034 (0.039) | -0.095 (0.066) | -0.093 (0.071) | 0.070 (0.070) | 0.097 (0.070) | 0.070 (0.064) |
| Happy Questions ×  Interest | -0.044 (0.040) | -0.008 (0.069) | 0.063 (0.074) | -0.036 (0.073) | 0.001 (0.073) | -0.009 (0.067) |
| Happy Writing ×  Interest | 0.053 (0.042) | -0.080 (0.071) | -0.025 (0.076) | 0.031 (0.075) | 0.131 (0.075) | -0.015 (0.069) |
| *N* | 1254 | 1254 | 1254 | 1254 | 1254 | 1254 |
| *R*-squared | 0.007 | 0.016 | 0.008 | 0.008 | 0.019 | 0.007 |

Note. Cell entries are OLS regression coefficients with associated standard errors in parentheses. FT = feeling thermometer. * FDR adjusted *p* < .05.

**Table H.3.6** The effects of interactions between happiness treatments and political interest on individual and subtractive affective polarization indicators (Study 3, NL)

|  | FT outparty | FT outparty supporters | FT out-ideologues | Social distance | Trait ratings | Outgroup trust | FT party (subt.) | FT party supporters (subt.) | FT ideology (subt.) |
| --- | --- | --- | --- | --- | --- | --- | --- | --- | --- |
| Intercept | 0.191* (0.019) | 0.231* (0.019) | 0.383* (0.018) | 0.542* (0.019) | 0.498* (0.013) | 0.497* (0.018) | 0.749* (0.014) | 0.722* (0.014) | 0.575* (0.015) |
| Happy Photos | 0.030 (0.038) | 0.003 (0.039) | 0.038 (0.037) | -0.015 (0.038) | 0.008 (0.027) | 0.018 (0.037) | -0.016 (0.027) | -0.018 (0.028) | -0.035 (0.030) |
| Happy Questions | 0.011 (0.040) | 0.021 (0.042) | 0.046 (0.039) | 0.062 (0.041) | 0.016 (0.029) | 0.011 (0.039) | -0.047 (0.029) | -0.053 (0.030) | -0.055 (0.032) |
| Happy Writing | 0.017 (0.041) | 0.004 (0.042) | -0.025 (0.040) | -0.065 (0.041) | -0.024 (0.029) | -0.032 (0.040) | -0.025 (0.029) | -0.029 (0.030) | -0.001 (0.033) |
| Interest | -0.083* (0.030) | -0.074 (0.031) | -0.013 (0.029) | 0.076 (0.031) | -0.013 (0.022) | 0.008 (0.030) | 0.104* (0.022) | 0.088* (0.023) | 0.065* (0.024) |
| Happy Photos ×  Interest | -0.052 (0.061) | -0.039 (0.063) | -0.078 (0.059) | 0.025 (0.062) | -0.022 (0.043) | -0.036 (0.059) | 0.035 (0.044) | 0.047 (0.045) | 0.082 (0.049) |
| Happy Questions ×  Interest | -0.005 (0.063) | -0.025 (0.066) | -0.076 (0.062) | -0.070 (0.065) | -0.041 (0.045) | -0.045 (0.062) | 0.060 (0.046) | 0.070 (0.048) | 0.084 (0.051) |
| Happy Writing ×  Interest | 0.003 (0.065) | 0.004 (0.068) | 0.054 (0.063) | 0.127 (0.066) | 0.045 (0.046) | 0.086 (0.064) | 0.026 (0.047) | 0.038 (0.049) | -0.006 (0.053) |
| *N* | 1254 | 1254 | 1254 | 1254 | 1254 | 1254 | 1254 | 1254 | 1254 |
| *R*-squared | 0.016 | 0.013 | 0.006 | 0.019 | 0.004 | 0.005 | 0.048 | 0.040 | 0.026 |

Note. Cell entries are OLS regression coefficients with associated standard errors in parentheses. FT = feeling thermometer. * FDR adjusted *p* < .05.

**Table H.3.7** The effects of interactions between happiness treatments and ideology on affective polarization, feeling thermometers toward social groups, conspiracy endorsement, and deep fake recognition (Study 3, NL)

|  | Affective polarization | FT immigrants | FT feminists | FT nationalists | Conspiracy | Deep fake |
| --- | --- | --- | --- | --- | --- | --- |
| Intercept | 0.371* (0.007) | 0.626* (0.011) | 0.617* (0.012) | 0.377* (0.012) | 0.268* (0.012) | 0.112* (0.012) |
| Happy Photos | -0.016 (0.014) | -0.009 (0.023) | -0.003 (0.025) | -0.001 (0.025) | -0.024 (0.024) | -0.016 (0.024) |
| Happy Questions | -0.010 (0.014) | 0.003 (0.023) | 0.026 (0.025) | -0.013 (0.025) | 0.012 (0.025) | -0.002 (0.024) |
| Happy Writing | -0.022 (0.015) | 0.052 (0.023) | 0.011 (0.025) | -0.043 (0.026) | -0.029 (0.025) | -0.019 (0.025) |
| Ideology (R) | 0.020 (0.010) | -0.178* (0.015) | -0.158* (0.017) | 0.114* (0.017) | 0.162* (0.017) | -0.006 (0.016) |
| Happy Photos ×  Ideology (R) | 0.019 (0.019) | 0.045 (0.031) | 0.021 (0.033) | 0.026 (0.034) | 0.050 (0.033) | -0.003 (0.032) |
| Happy Questions ×  Ideology (R) | 0.021 (0.020) | 0.030 (0.031) | -0.014 (0.034) | 0.021 (0.034) | 0.001 (0.033) | 0.052 (0.033) |
| Happy Writing ×  Ideology (R) | 0.062* (0.020) | -0.011 (0.032) | 0.029 (0.035) | 0.085 (0.035) | 0.012 (0.035) | 0.008 (0.034) |
| *N* | 1254 | 1254 | 1254 | 1254 | 1254 | 1254 |
| *R*-squared | 0.030 | 0.161 | 0.116 | 0.094 | 0.150 | 0.007 |

Note. Cell entries are OLS regression coefficients with associated standard errors in parentheses. FT = feeling thermometer. * FDR adjusted *p* < .05.

**Table H.3.8** The effects of interactions between happiness treatments and ideology on individual and subtractive affective polarization indicators (Study 3, NL)

|  | FT outparty | FT outparty supporters | FT out-ideologues | Social distance | Trait ratings | Outgroup trust | FT party (subt.) | FT party supporters (subt.) | FT ideology (subt.) |
| --- | --- | --- | --- | --- | --- | --- | --- | --- | --- |
| Intercept | 0.130* (0.011) | 0.177* (0.012) | 0.360* (0.011) | 0.563* (0.011) | 0.479* (0.008) | 0.515* (0.011) | 0.822* (0.008) | 0.785* (0.008) | 0.637* (0.009) |
| Happy Photos | -0.022 (0.022) | -0.033 (0.023) | -0.012 (0.022) | -0.018 (0.023) | -0.007 (0.016) | -0.005 (0.022) | 0.008 (0.016) | 0.016 (0.017) | 0.003 (0.018) |
| Happy Questions | -0.020 (0.023) | -0.021 (0.024) | 0.005 (0.022) | 0.022 (0.023) | -0.020 (0.016) | -0.026 (0.022) | 0.009 (0.017) | 0.007 (0.017) | -0.014 (0.018) |
| Happy Writing | -0.035 (0.023) | -0.041 (0.024) | -0.028 (0.023) | -0.005 (0.024) | -0.021 (0.016) | -0.003 (0.023) | 0.024 (0.017) | 0.025 (0.017) | 0.015 (0.019) |
| Ideology (R) | 0.028 (0.015) | 0.022 (0.016) | 0.029 (0.015) | 0.043* (0.016) | 0.022 (0.011) | -0.025 (0.015) | -0.027 (0.011) | -0.026 (0.012) | -0.049* (0.012) |
| Happy Photos ×  Ideology (R) | 0.040 (0.030) | 0.023 (0.032) | 0.010 (0.030) | 0.032 (0.031) | 0.003 (0.022) | 0.007 (0.030) | -0.005 (0.022) | -0.013 (0.023) | 0.018 (0.025) |
| Happy Questions ×  Ideology (R) | 0.047 (0.031) | 0.046 (0.032) | -0.009 (0.030) | 0.000 (0.032) | 0.021 (0.022) | 0.021 (0.030) | -0.031 (0.023) | -0.031 (0.023) | 0.020 (0.025) |
| Happy Writing ×  Ideology (R) | 0.102* (0.032) | 0.091* (0.033) | 0.065 (0.031) | 0.030 (0.033) | 0.045 (0.023) | 0.040 (0.031) | -0.063* (0.024) | -0.060 (0.024) | -0.038 (0.026) |
| *N* | 1254 | 1254 | 1254 | 1254 | 1254 | 1254 | 1254 | 1254 | 1254 |
| *R*-squared | 0.031 | 0.022 | 0.015 | 0.021 | 0.019 | 0.005 | 0.029 | 0.027 | 0.028 |

Note. Cell entries are OLS regression coefficients with associated standard errors in parentheses. FT = feeling thermometer. * FDR adjusted *p* < .05.

**Table H.3.9** The effects of interactions between happiness treatments and happiness strength on affective polarization, feeling thermometers toward social groups, conspiracy endorsement, and deep fake recognition (Study 3, NL)

|  | Affective polarization | FT immigrants | FT feminists | FT nationalists | Conspiracy | Deep fake |
| --- | --- | --- | --- | --- | --- | --- |
| Intercept | 0.352* (0.018) | 0.439* (0.030) | 0.512* (0.032) | 0.477* (0.032) | 0.401* (0.032) | 0.068 (0.029) |
| Happy Photos | -0.050 (0.034) | 0.034 (0.059) | 0.000 (0.063) | -0.107 (0.063) | -0.216* (0.063) | 0.028 (0.057) |
| Happy Questions | 0.018 (0.036) | 0.093 (0.062) | 0.034 (0.066) | -0.079 (0.066) | -0.002 (0.066) | -0.027 (0.060) |
| Happy Writing | -0.051 (0.039) | 0.158 (0.066) | 0.016 (0.071) | -0.035 (0.070) | -0.090 (0.071) | 0.078 (0.065) |
| Happiness | 0.043 (0.025) | 0.137* (0.043) | 0.032 (0.046) | -0.059 (0.045) | -0.070 (0.045) | 0.060 (0.041) |
| Happy Photos ×  Happiness | 0.057 (0.045) | -0.041 (0.078) | 0.004 (0.083) | 0.169 (0.083) | 0.305* (0.083) | -0.067 (0.076) |
| Happy Questions ×  Happiness | -0.024 (0.050) | -0.113 (0.085) | -0.026 (0.091) | 0.115 (0.091) | 0.026 (0.091) | 0.074 (0.083) |
| Happy Writing ×  Happiness | 0.080 (0.052) | -0.158 (0.089) | 0.014 (0.095) | 0.052 (0.094) | 0.094 (0.094) | -0.130 (0.086) |
| *N* | 1254 | 1254 | 1254 | 1254 | 1254 | 1254 |
| *R*-squared | 0.015 | 0.016 | 0.003 | 0.004 | 0.014 | 0.010 |

Note. Cell entries are OLS regression coefficients with associated standard errors in parentheses. FT = feeling thermometer. * FDR adjusted *p* < .05.

**Table H.3.10** The effects of interactions between happiness treatments and happiness strength on individual and subtractive affective polarization indicators (Study 3, NL)

|  | FT outparty | FT outparty supporters | FT out-ideologues | Social distance | Trait ratings | Outgroup trust | FT party (subt.) | FT party supporters (subt.) | FT ideology (subt.) |
| --- | --- | --- | --- | --- | --- | --- | --- | --- | --- |
| Intercept | 0.151* (0.028) | 0.164* (0.029) | 0.351* (0.027) | 0.515* (0.028) | 0.454* (0.020) | 0.476* (0.027) | 0.778* (0.020) | 0.762* (0.021) | 0.617* (0.023) |
| Happy Photos | -0.111 (0.055) | -0.101 (0.056) | -0.015 (0.053) | -0.064 (0.055) | 0.009 (0.039) | -0.022 (0.053) | 0.076 (0.040) | 0.050 (0.041) | 0.004 (0.044) |
| Happy Questions | 0.014 (0.057) | 0.068 (0.059) | 0.022 (0.055) | 0.056 (0.057) | -0.024 (0.040) | -0.030 (0.055) | -0.025 (0.042) | -0.048 (0.043) | -0.065 (0.046) |
| Happy Writing | -0.007 (0.061) | -0.016 (0.063) | -0.003 (0.060) | -0.175* (0.062) | -0.038 (0.043) | -0.070 (0.059) | 0.011 (0.045) | 0.021 (0.047) | 0.020 (0.050) |
| Happiness | -0.008 (0.039) | 0.037 (0.041) | 0.037 (0.038) | 0.104* (0.040) | 0.054 (0.028) | 0.038 (0.038) | 0.045 (0.029) | 0.013 (0.030) | -0.008 (0.032) |
| Happy Photos ×  Happiness | 0.149 (0.072) | 0.105 (0.075) | 0.009 (0.070) | 0.076 (0.072) | -0.023 (0.051) | 0.023 (0.070) | -0.100 (0.053) | -0.056 (0.055) | 0.012 (0.059) |
| Happy Questions ×  Happiness | -0.012 (0.079) | -0.092 (0.082) | -0.031 (0.077) | -0.050 (0.079) | 0.020 (0.056) | 0.020 (0.077) | 0.022 (0.058) | 0.054 (0.060) | 0.088 (0.064) |
| Happy Writing ×  Happiness | 0.033 (0.082) | 0.027 (0.085) | 0.009 (0.080) | 0.244* (0.082) | 0.050 (0.058) | 0.117 (0.080) | -0.029 (0.060) | -0.036 (0.062) | -0.032 (0.067) |
| *N* | 1254 | 1254 | 1254 | 1254 | 1254 | 1254 | 1254 | 1254 | 1254 |
| *R*-squared | 0.006 | 0.008 | 0.002 | 0.032 | 0.009 | 0.009 | 0.006 | 0.004 | 0.003 |

Note. Cell entries are OLS regression coefficients with associated standard errors in parentheses. FT = feeling thermometer. * FDR adjusted *p* < .05.

**Table H.4.1** The effects of emotion treatments on affective polarization, feeling thermometers toward social groups, conspiracy endorsement, and deep fake recognition (Study 4, US)

|  | Affective polarization | FT immigrants | FT feminists | FT neo-Nazis | Conspiracy | Deep fake |
| --- | --- | --- | --- | --- | --- | --- |
| Intercept | 0.410* (0.012) | 0.608* (0.016) | 0.498* (0.017) | 0.126* (0.014) | 0.373* (0.014) | 0.142* (0.015) |
| Angry Writing | -0.003 (0.016) | 0.002 (0.023) | 0.039 (0.024) | 0.009 (0.019) | 0.005 (0.020) | 0.032 (0.021) |
| Anxious Writing | -0.009 (0.016) | -0.031 (0.023) | 0.000 (0.024) | -0.001 (0.019) | 0.011 (0.020) | 0.016 (0.021) |
| Happy Writing | -0.001 (0.017) | -0.004 (0.023) | 0.016 (0.024) | -0.003 (0.020) | 0.023 (0.020) | -0.006 (0.021) |
| *N* | 1165 | 1165 | 1165 | 1165 | 1165 | 1165 |
| *R*-squared | 0.000 | 0.003 | 0.003 | 0.000 | 0.001 | 0.004 |

Note. Cell entries are OLS regression coefficients with associated standard errors in parentheses. FT = feeling thermometer. * FDR adjusted *p* < .05.

**Table H.4.2** The effects of emotion treatments on individual and subtractive affective polarization indicators (Study 4, US)

|  | FT outparty | FT outparty supporters | FT out-ideologues | Social distance | Trait ratings | Outgroup trust | FT party (subt.) | FT party supporters (subt.) | FT ideology (subt.) |
| --- | --- | --- | --- | --- | --- | --- | --- | --- | --- |
| Intercept | 0.263* (0.016) | 0.302* (0.016) | 0.371* (0.020) | 0.635* (0.015) | 0.434* (0.012) | 0.436* (0.016) | 0.756* (0.011) | 0.742* (0.011) | 0.692* (0.014) |
| Angry Writing | -0.007 (0.022) | -0.010 (0.021) | -0.026 (0.027) | 0.008 (0.021) | -0.014 (0.017) | 0.013 (0.022) | 0.011 (0.015) | 0.010 (0.015) | 0.014 (0.020) |
| Anxious Writing | 0.002 (0.022) | 0.001 (0.021) | -0.013 (0.027) | -0.024 (0.021) | -0.016 (0.017) | -0.010 (0.022) | 0.008 (0.015) | 0.005 (0.015) | 0.008 (0.020) |
| Happy Writing | 0.000 (0.022) | 0.008 (0.022) | -0.006 (0.028) | -0.004 (0.021) | -0.006 (0.017) | 0.003 (0.022) | 0.009 (0.016) | 0.003 (0.016) | -0.003 (0.020) |
| *N* | 1165 | 1165 | 873 | 1165 | 1165 | 1165 | 1165 | 1165 | 873 |
| *R*-squared | 0.000 | 0.001 | 0.001 | 0.002 | 0.001 | 0.001 | 0.001 | 0.000 | 0.001 |

Note. Cell entries are OLS regression coefficients with associated standard errors in parentheses. FT = feeling thermometer. * FDR adjusted *p* < .05.

**Table H.4.3** The effects of interactions between emotion treatments and political identity strength on affective polarization, feeling thermometers toward social groups, conspiracy endorsement, and deep fake recognition (Study 4, US)

|  | Affective polarization | FT immigrants | FT feminists | FT neo-Nazis | Conspiracy | Deep fake |
| --- | --- | --- | --- | --- | --- | --- |
| Intercept | 0.557* (0.043) | 0.504* (0.061) | 0.283* (0.064) | 0.031 (0.053) | 0.187* (0.053) | 0.094 (0.056) |
| Angry Writing | 0.042 (0.060) | 0.024 (0.085) | 0.277* (0.090) | 0.065 (0.073) | 0.025 (0.073) | -0.033 (0.078) |
| Anxious Writing | 0.040 (0.058) | 0.139 (0.083) | 0.314* (0.087) | 0.044 (0.071) | 0.013 (0.071) | -0.006 (0.076) |
| Happy Writing | 0.015 (0.060) | 0.040 (0.086) | 0.210 (0.091) | 0.087 (0.074) | 0.130 (0.074) | -0.001 (0.079) |
| Pids | -0.209* (0.058) | 0.147 (0.083) | 0.305* (0.088) | 0.135 (0.072) | 0.265* (0.072) | 0.069 (0.077) |
| Angry Writing ×  Pids | -0.062 (0.081) | -0.031 (0.116) | -0.338* (0.122) | -0.079 (0.100) | -0.030 (0.100) | 0.091 (0.107) |
| Anxious Writing ×  Pids | -0.068 (0.078) | -0.240 (0.112) | -0.445* (0.118) | -0.064 (0.097) | -0.005 (0.097) | 0.031 (0.103) |
| Happy Writing ×  Pids | -0.022 (0.082) | -0.062 (0.118) | -0.275 (0.124) | -0.127 (0.102) | -0.151 (0.101) | -0.008 (0.108) |
| *N* | 1165 | 1165 | 1165 | 1165 | 1165 | 1165 |
| *R*-squared | 0.064 | 0.009 | 0.016 | 0.005 | 0.038 | 0.011 |

Note. Cell entries are OLS regression coefficients with associated standard errors in parentheses. FT = feeling thermometer. * FDR adjusted *p* < .05.

**Table H.4.4** The effects of interactions between emotion treatments and political identity strength on individual and subtractive affective polarization indicators (Study 4, US)

|  | FT outparty | FT outparty supporters | FT out-ideologues | Social distance | Trait ratings | Outgroup trust | FT party (subt.) | FT party supporters (subt.) | FT ideology (subt.) |
| --- | --- | --- | --- | --- | --- | --- | --- | --- | --- |
| Intercept | 0.447* (0.057) | 0.562* (0.056) | 0.283* (0.077) | 0.761* (0.056) | 0.595* (0.045) | 0.552* (0.058) | 0.425* (0.037) | 0.396* (0.036) | 0.637* (0.055) |
| Angry Writing | 0.007 (0.080) | -0.046 (0.078) | 0.223 (0.111) | -0.003 (0.078) | 0.009 (0.063) | 0.125 (0.081) | 0.017 (0.051) | 0.037 (0.050) | -0.119 (0.080) |
| Anxious Writing | 0.049 (0.078) | 0.023 (0.075) | 0.174 (0.106) | -0.007 (0.075) | 0.010 (0.061) | 0.029 (0.078) | 0.001 (0.050) | -0.013 (0.048) | -0.123 (0.077) |
| Happy Writing | 0.085 (0.081) | -0.008 (0.079) | 0.227 (0.109) | -0.081 (0.079) | 0.003 (0.064) | -0.022 (0.082) | 0.030 (0.052) | 0.050 (0.050) | -0.189 (0.079) |
| Pids | -0.262* (0.078) | -0.368* (0.076) | 0.121 (0.101) | -0.179 (0.076) | -0.227* (0.062) | -0.165 (0.079) | 0.470* (0.050) | 0.490* (0.049) | 0.074 (0.073) |
| Angry Writing ×  Pids | -0.019 (0.109) | 0.052 (0.106) | -0.339 (0.147) | 0.017 (0.106) | -0.031 (0.086) | -0.157 (0.111) | -0.010 (0.070) | -0.040 (0.068) | 0.179 (0.106) |
| Anxious Writing ×  Pids | -0.066 (0.106) | -0.030 (0.103) | -0.253 (0.139) | -0.024 (0.102) | -0.034 (0.083) | -0.054 (0.107) | 0.007 (0.067) | 0.022 (0.066) | 0.173 (0.101) |
| Happy Writing ×  Pids | -0.118 (0.111) | 0.025 (0.108) | -0.317 (0.144) | 0.108 (0.107) | -0.012 (0.087) | 0.037 (0.112) | -0.032 (0.071) | -0.069 (0.069) | 0.251 (0.104) |
| *N* | 1165 | 1165 | 873 | 1165 | 1165 | 1165 | 1165 | 1165 | 873 |
| *R*-squared | 0.057 | 0.077 | 0.014 | 0.019 | 0.057 | 0.029 | 0.241 | 0.259 | 0.050 |

Note. Cell entries are OLS regression coefficients with associated standard errors in parentheses. FT = feeling thermometer. * FDR adjusted *p* < .05.

**Table H.4.5** The effects of interactions between emotion treatments and political interest on affective polarization, feeling thermometers toward social groups, conspiracy endorsement, and deep fake recognition (Study 4, US)

|  | Affective polarization | FT immigrants | FT feminists | FT neo-Nazis | Conspiracy | Deep fake |
| --- | --- | --- | --- | --- | --- | --- |
| Intercept | 0.402* (0.028) | 0.493* (0.039) | 0.426* (0.041) | 0.106* (0.034) | 0.393* (0.034) | 0.128* (0.036) |
| Angry Writing | 0.023 (0.039) | 0.012 (0.053) | 0.071 (0.057) | 0.078 (0.046) | 0.010 (0.047) | 0.087 (0.049) |
| Anxious Writing | 0.026 (0.040) | -0.015 (0.055) | 0.055 (0.059) | 0.020 (0.048) | -0.008 (0.049) | 0.026 (0.051) |
| Happy Writing | 0.019 (0.040) | 0.035 (0.055) | 0.042 (0.059) | -0.014 (0.048) | 0.021 (0.049) | -0.033 (0.051) |
| Interest | 0.012 (0.040) | 0.179* (0.055) | 0.113 (0.059) | 0.031 (0.048) | -0.030 (0.049) | 0.023 (0.051) |
| Angry Writing ×  Interest | -0.041 (0.055) | -0.016 (0.075) | -0.050 (0.080) | -0.107 (0.065) | -0.008 (0.066) | -0.084 (0.070) |
| Anxious Writing ×  Interest | -0.054 (0.057) | -0.031 (0.078) | -0.087 (0.083) | -0.032 (0.068) | 0.029 (0.069) | -0.016 (0.072) |
| Happy Writing ×  Interest | -0.031 (0.058) | -0.056 (0.079) | -0.039 (0.085) | 0.019 (0.069) | 0.003 (0.070) | 0.044 (0.074) |
| *N* | 1165 | 1165 | 1165 | 1165 | 1165 | 1165 |
| *R*-squared | 0.002 | 0.030 | 0.009 | 0.004 | 0.003 | 0.007 |

Note. Cell entries are OLS regression coefficients with associated standard errors in parentheses. FT = feeling thermometer. * FDR adjusted *p* < .05.

**Table H.4.6** The effects of interactions between emotion treatments and political interest on individual and subtractive affective polarization indicators (Study 4, US)

|  | FT outparty | FT outparty supporters | FT out-ideologues | Social distance | Trait ratings | Outgroup trust | FT party (subt.) | FT party supporters (subt.) | FT ideology (subt.) |
| --- | --- | --- | --- | --- | --- | --- | --- | --- | --- |
| Intercept | 0.262* (0.038) | 0.316* (0.037) | 0.373* (0.052) | 0.630* (0.036) | 0.452* (0.030) | 0.347* (0.038) | 0.720* (0.027) | 0.688* (0.026) | 0.597* (0.038) |
| Angry Writing | 0.028 (0.052) | -0.006 (0.051) | -0.013 (0.069) | 0.019 (0.049) | 0.010 (0.041) | 0.092 (0.052) | 0.014 (0.037) | 0.030 (0.036) | 0.043 (0.050) |
| Anxious Writing | 0.094 (0.054) | 0.052 (0.053) | 0.008 (0.075) | -0.078 (0.051) | 0.003 (0.042) | 0.041 (0.054) | -0.069 (0.038) | -0.049 (0.037) | 0.043 (0.054) |
| Happy Writing | 0.034 (0.054) | 0.018 (0.053) | 0.023 (0.072) | -0.052 (0.051) | 0.016 (0.042) | 0.077 (0.053) | -0.002 (0.038) | -0.003 (0.037) | -0.009 (0.052) |
| Interest | 0.001 (0.053) | -0.021 (0.053) | -0.002 (0.070) | 0.006 (0.051) | -0.028 (0.042) | 0.138 (0.053) | 0.057 (0.038) | 0.084 (0.037) | 0.139* (0.051) |
| Angry Writing ×  Interest | -0.054 (0.073) | -0.007 (0.072) | -0.019 (0.095) | -0.018 (0.069) | -0.038 (0.057) | -0.124 (0.073) | -0.005 (0.051) | -0.032 (0.051) | -0.041 (0.069) |
| Anxious Writing ×  Interest | -0.138 (0.075) | -0.076 (0.074) | -0.029 (0.100) | 0.080 (0.072) | -0.027 (0.059) | -0.082 (0.075) | 0.114 (0.053) | 0.077 (0.052) | -0.055 (0.073) |
| Happy Writing ×  Interest | -0.055 (0.077) | -0.016 (0.076) | -0.045 (0.100) | 0.076 (0.073) | -0.036 (0.061) | -0.114 (0.077) | 0.020 (0.054) | 0.011 (0.053) | 0.014 (0.072) |
| *N* | 1165 | 1165 | 873 | 1165 | 1165 | 1165 | 1165 | 1165 | 873 |
| *R*-squared | 0.008 | 0.004 | 0.002 | 0.007 | 0.007 | 0.008 | 0.025 | 0.028 | 0.027 |

Note. Cell entries are OLS regression coefficients with associated standard errors in parentheses. FT = feeling thermometer. * FDR adjusted *p* < .05.

**Table H.4.7** The effects of interactions between emotion treatments and partisanship on affective polarization, feeling thermometers toward social groups, conspiracy endorsement, and deep fake recognition (Study 4, US)

|  | Affective polarization | FT immigrants | FT feminists | FT neo-Nazis | Conspiracy | Deep fake |
| --- | --- | --- | --- | --- | --- | --- |
| Intercept | 0.396* (0.017) | 0.709* (0.021) | 0.639* (0.022) | 0.112* (0.020) | 0.338* (0.020) | 0.177* (0.021) |
| Angry Writing | 0.005 (0.023) | 0.025 (0.029) | 0.038 (0.029) | 0.036 (0.027) | 0.012 (0.027) | 0.040 (0.029) |
| Anxious Writing | 0.015 (0.023) | -0.026 (0.030) | 0.015 (0.030) | 0.015 (0.028) | 0.003 (0.028) | -0.005 (0.030) |
| Happy Writing | 0.038 (0.023) | 0.005 (0.030) | 0.000 (0.030) | 0.031 (0.028) | 0.022 (0.028) | 0.003 (0.030) |
| Partisanship (R) | 0.027 (0.024) | -0.199* (0.030) | -0.277* (0.030) | 0.027 (0.028) | 0.069 (0.028) | -0.067 (0.030) |
| Angry Writing ×  Partisanship (R) | -0.015 (0.032) | -0.062 (0.041) | -0.016 (0.042) | -0.053 (0.039) | -0.010 (0.039) | -0.022 (0.041) |
| Anxious Writing ×  Partisanship (R) | -0.046 (0.032) | -0.003 (0.041) | -0.021 (0.041) | -0.032 (0.039) | 0.013 (0.039) | 0.042 (0.041) |
| Happy Writing ×  Partisanship (R) | -0.078 (0.033) | -0.025 (0.042) | 0.022 (0.042) | -0.068 (0.040) | 0.005 (0.040) | -0.020 (0.042) |
| *N* | 1165 | 1165 | 1165 | 1165 | 1165 | 1165 |
| *R*-squared | 0.007 | 0.173 | 0.246 | 0.004 | 0.024 | 0.024 |

Note. Cell entries are OLS regression coefficients with associated standard errors in parentheses. FT = feeling thermometer. * FDR adjusted *p* < .05.

**Table H.4.8** The effects of interactions between emotion treatments and partisanship on individual and subtractive affective polarization indicators (Study 4, US)

|  | FT outparty | FT outparty supporters | FT out-ideologues | Social distance | Trait ratings | Outgroup trust | FT party (subt.) | FT party supporters (subt.) | FT ideology (subt.) |
| --- | --- | --- | --- | --- | --- | --- | --- | --- | --- |
| Intercept | 0.248* (0.023) | 0.283* (0.022) | 0.415* (0.029) | 0.615* (0.021) | 0.402* (0.018) | 0.414* (0.023) | 0.782* (0.016) | 0.769* (0.016) | 0.660* (0.021) |
| Angry Writing | 0.004 (0.031) | -0.005 (0.030) | -0.017 (0.039) | -0.005 (0.029) | 0.012 (0.024) | 0.023 (0.031) | -0.003 (0.022) | 0.002 (0.021) | 0.025 (0.029) |
| Anxious Writing | 0.028 (0.031) | 0.019 (0.031) | 0.036 (0.040) | -0.016 (0.030) | 0.009 (0.025) | 0.015 (0.031) | -0.024 (0.022) | -0.022 (0.022) | -0.032 (0.029) |
| Happy Writing | 0.037 (0.031) | 0.050 (0.031) | 0.038 (0.040) | 0.020 (0.030) | 0.043 (0.025) | 0.046 (0.031) | -0.029 (0.022) | -0.036 (0.022) | -0.032 (0.030) |
| Partisanship (R) | 0.028 (0.032) | 0.038 (0.031) | -0.077 (0.039) | 0.039 (0.030) | 0.064 (0.025) | 0.044 (0.032) | -0.049 (0.022) | -0.053 (0.022) | 0.056 (0.028) |
| Angry Writing ×  Partisanship (R) | -0.021 (0.044) | -0.009 (0.043) | -0.023 (0.053) | 0.029 (0.041) | -0.051 (0.034) | -0.018 (0.043) | 0.026 (0.031) | 0.012 (0.030) | -0.017 (0.039) |
| Anxious Writing ×  Partisanship (R) | -0.051 (0.043) | -0.036 (0.043) | -0.086 (0.053) | -0.018 (0.041) | -0.049 (0.034) | -0.048 (0.043) | 0.062 (0.031) | 0.052 (0.030) | 0.072 (0.039) |
| Happy Writing ×  Partisanship (R) | -0.075 (0.044) | -0.084 (0.044) | -0.084 (0.054) | -0.048 (0.042) | -0.098* (0.035) | -0.086 (0.044) | 0.075 (0.032) | 0.076 (0.031) | 0.055 (0.040) |
| *N* | 1165 | 1165 | 873 | 1165 | 1165 | 1165 | 1165 | 1165 | 873 |
| *R*-squared | 0.003 | 0.005 | 0.055 | 0.009 | 0.009 | 0.005 | 0.007 | 0.009 | 0.049 |

Note. Cell entries are OLS regression coefficients with associated standard errors in parentheses. FT = feeling thermometer. * FDR adjusted *p* < .05.

**Table H.4.9** The effects of interactions between emotion treatments and ideology on affective polarization, feeling thermometers toward social groups, conspiracy endorsement, and deep fake recognition (Study 4, US)

|  | Affective polarization | FT immigrants | FT feminists | FT neo-Nazis | Conspiracy | Deep fake |
| --- | --- | --- | --- | --- | --- | --- |
| Intercept | 0.394* (0.026) | 0.732* (0.035) | 0.699* (0.035) | 0.030 (0.030) | 0.203* (0.029) | 0.102* (0.033) |
| Angry Writing | -0.013 (0.036) | 0.021 (0.048) | 0.029 (0.049) | 0.000 (0.042) | -0.036 (0.040) | 0.076 (0.046) |
| Anxious Writing | -0.012 (0.036) | 0.008 (0.049) | 0.057 (0.049) | 0.006 (0.043) | -0.039 (0.040) | 0.036 (0.047) |
| Happy Writing | 0.002 (0.039) | 0.083 (0.052) | 0.081 (0.052) | -0.002 (0.045) | -0.022 (0.043) | 0.023 (0.049) |
| Ideology | 0.027 (0.040) | -0.212* (0.053) | -0.344* (0.054) | 0.165* (0.046) | 0.291* (0.044) | 0.069 (0.051) |
| Angry Writing ×  Ideology | 0.017 (0.055) | -0.031 (0.073) | 0.020 (0.074) | 0.014 (0.064) | 0.066 (0.060) | -0.075 (0.070) |
| Anxious Writing ×  Ideology | 0.003 (0.055) | -0.052 (0.073) | -0.075 (0.074) | -0.020 (0.064) | 0.066 (0.060) | -0.036 (0.070) |
| Happy Writing ×  Ideology | -0.007 (0.058) | -0.128 (0.077) | -0.084 (0.078) | -0.010 (0.068) | 0.055 (0.064) | -0.050 (0.074) |
| *N* | 1165 | 1165 | 1165 | 1165 | 1165 | 1165 |
| *R*-squared | 0.003 | 0.085 | 0.155 | 0.042 | 0.179 | 0.006 |

Note. Cell entries are OLS regression coefficients with associated standard errors in parentheses. FT = feeling thermometer. * FDR adjusted *p* < .05.

**Table H.4.10** The effects of interactions between emotion treatments and ideology on individual and subtractive affective polarization indicators (Study 4, US)

|  | FT outparty | FT outparty supporters | FT out-ideologues | Social distance | Trait ratings | Outgroup trust | FT party (subt.) | FT party supporters (subt.) | FT ideology (subt.) |
| --- | --- | --- | --- | --- | --- | --- | --- | --- | --- |
| Intercept | 0.220* (0.035) | 0.279* (0.034) | 0.338* (0.041) | 0.628* (0.033) | 0.422* (0.027) | 0.450* (0.035) | 0.762* (0.025) | 0.742* (0.024) | 0.695* (0.030) |
| Angry Writing | -0.019 (0.048) | -0.021 (0.047) | -0.019 (0.057) | -0.031 (0.046) | -0.020 (0.038) | 0.001 (0.048) | 0.023 (0.034) | 0.019 (0.034) | 0.046 (0.042) |
| Anxious Writing | 0.002 (0.049) | -0.012 (0.048) | -0.005 (0.058) | -0.038 (0.046) | 0.004 (0.038) | -0.039 (0.049) | -0.003 (0.035) | -0.009 (0.034) | 0.003 (0.042) |
| Happy Writing | 0.023 (0.052) | -0.011 (0.051) | 0.004 (0.062) | -0.024 (0.049) | 0.023 (0.041) | -0.008 (0.052) | -0.005 (0.037) | -0.001 (0.036) | -0.004 (0.045) |
| Ideology | 0.074 (0.053) | 0.040 (0.052) | 0.055 (0.058) | 0.012 (0.050) | 0.021 (0.042) | -0.023 (0.053) | -0.009 (0.038) | 0.000 (0.037) | -0.005 (0.043) |
| Angry Writing ×  Ideology | 0.019 (0.073) | 0.018 (0.072) | -0.012 (0.080) | 0.065 (0.069) | 0.009 (0.057) | 0.021 (0.073) | -0.019 (0.052) | -0.016 (0.051) | -0.051 (0.059) |
| Anxious Writing ×  Ideology | -0.005 (0.073) | 0.019 (0.072) | -0.016 (0.081) | 0.022 (0.069) | -0.032 (0.057) | 0.048 (0.073) | 0.018 (0.052) | 0.022 (0.051) | 0.008 (0.059) |
| Happy Writing ×  Ideology | -0.041 (0.077) | 0.028 (0.076) | -0.019 (0.086) | 0.030 (0.074) | -0.048 (0.061) | 0.020 (0.077) | 0.022 (0.055) | 0.006 (0.054) | 0.002 (0.063) |
| *N* | 1165 | 1165 | 873 | 1165 | 1165 | 1165 | 1165 | 1165 | 873 |
| *R*-squared | 0.007 | 0.005 | 0.004 | 0.006 | 0.002 | 0.001 | 0.001 | 0.001 | 0.003 |

Note. Cell entries are OLS regression coefficients with associated standard errors in parentheses. FT = feeling thermometer. * FDR adjusted *p* < .05.

**Table H.4.11** The effects of interactions between emotion treatments and emotion strength on affective polarization, feeling thermometers toward social groups, conspiracy endorsement, and deep fake recognition (Study 4, US)

|  | Affective polarization | FT immigrants | FT feminists | FT neo-Nazis | Conspiracy | Deep fake |
| --- | --- | --- | --- | --- | --- | --- |
| Intercept | 0.417* (0.020) | 0.601* (0.027) | 0.560* (0.029) | 0.165* (0.023) | 0.423* (0.024) | 0.178* (0.025) |
| Angry Writing | -0.010 (0.027) | 0.020 (0.037) | -0.027 (0.039) | 0.021 (0.031) | 0.001 (0.032) | 0.013 (0.034) |
| Anxious Writing | 0.003 (0.027) | -0.070 (0.037) | -0.072 (0.039) | 0.042 (0.032) | 0.026 (0.032) | 0.052 (0.035) |
| Emotion Strength | -0.018 (0.033) | 0.007 (0.046) | -0.098 (0.049) | -0.087 (0.039) | -0.055 (0.040) | -0.088 (0.043) |
| Angry Writing ×  Emotion Strength | 0.016 (0.045) | -0.032 (0.062) | 0.106 (0.066) | -0.025 (0.053) | -0.046 (0.054) | 0.050 (0.058) |
| Anxious Writing ×  Emotion Strength | -0.022 (0.045) | 0.087 (0.062) | 0.116 (0.065) | -0.078 (0.052) | -0.075 (0.054) | -0.057 (0.057) |
| *N* | 893 | 893 | 893 | 893 | 893 | 893 |
| *R*-squared | 0.003 | 0.009 | 0.008 | 0.041 | 0.026 | 0.025 |

Note. Cell entries are OLS regression coefficients with associated standard errors in parentheses. FT = feeling thermometer. * FDR adjusted *p* < .05. Happy writing is the reference group.

**Table H.4.12** The effects of interactions between emotion treatments and emotion strength on individual and subtractive affective polarization indicators (Study 4, US)

|  | FT outparty | FT outparty supporters | FT out-ideologues | Social distance | Trait ratings | Outgroup trust | FT party (subt.) | FT party supporters (subt.) | FT ideology (subt.) |
| --- | --- | --- | --- | --- | --- | --- | --- | --- | --- |
| Intercept | 0.299* (0.026) | 0.325* (0.026) | 0.386* (0.034) | 0.618* (0.025) | 0.418* (0.021) | 0.447* (0.026) | 0.746* (0.019) | 0.743* (0.019) | 0.677* (0.025) |
| Angry Writing | -0.048 (0.035) | -0.044 (0.035) | -0.026 (0.046) | 0.029 (0.034) | 0.006 (0.028) | 0.004 (0.035) | 0.033 (0.025) | 0.017 (0.025) | 0.021 (0.034) |
| Anxious Writing | 0.000 (0.036) | 0.015 (0.036) | 0.010 (0.045) | -0.006 (0.035) | 0.020 (0.028) | -0.024 (0.036) | 0.001 (0.026) | -0.012 (0.025) | -0.008 (0.034) |
| Emotion Strength | -0.075 (0.045) | -0.030 (0.044) | -0.043 (0.056) | 0.026 (0.043) | 0.022 (0.035) | -0.016 (0.044) | 0.040 (0.032) | 0.003 (0.031) | 0.023 (0.042) |
| Angry Writing ×  Emotion Strength | 0.086 (0.060) | 0.054 (0.060) | 0.011 (0.076) | -0.037 (0.058) | -0.029 (0.047) | 0.013 (0.060) | -0.067 (0.043) | -0.022 (0.042) | -0.008 (0.057) |
| Anxious Writing ×  Emotion Strength | 0.006 (0.060) | -0.043 (0.059) | -0.033 (0.074) | -0.030 (0.057) | -0.060 (0.046) | 0.024 (0.059) | -0.006 (0.043) | 0.029 (0.042) | 0.039 (0.055) |
| *N* | 893 | 893 | 665 | 893 | 893 | 893 | 893 | 893 | 665 |
| *R*-squared | 0.007 | 0.006 | 0.006 | 0.003 | 0.003 | 0.002 | 0.004 | 0.002 | 0.006 |

Note. Cell entries are OLS regression coefficients with associated standard errors in parentheses. FT = feeling thermometer. * FDR adjusted *p* < .05. Happy writing is the reference group.

**Table H.4.13** The effects of interactions between emotion treatments and emotion strength on affective polarization, feeling thermometers toward social groups, conspiracy endorsement, and deep fake recognition (Study 4, US)

|  | Affective polarization | FT immigrants | FT feminists | FT neo-Nazis | Conspiracy | Deep fake |
| --- | --- | --- | --- | --- | --- | --- |
| Intercept | 0.407* (0.018) | 0.621* (0.025) | 0.533* (0.026) | 0.186* (0.021) | 0.424* (0.021) | 0.191* (0.023) |
| Anxious Writing | 0.013 (0.026) | -0.091 (0.035) | -0.045 (0.037) | 0.020 (0.030) | 0.025 (0.031) | 0.039 (0.033) |
| Happy Writing | 0.010 (0.027) | -0.020 (0.037) | 0.027 (0.039) | -0.021 (0.031) | -0.001 (0.032) | -0.013 (0.034) |
| Emotion Strength | -0.001 (0.030) | -0.025 (0.042) | 0.008 (0.044) | -0.112* (0.035) | -0.101* (0.036) | -0.037 (0.039) |
| Anxious Writing ×  Emotion Strength | -0.038 (0.042) | 0.119 (0.059) | 0.010 (0.062) | -0.053 (0.050) | -0.029 (0.051) | -0.108 (0.054) |
| Happy Writing ×  Emotion Strength | -0.016 (0.045) | 0.032 (0.062) | -0.106 (0.066) | 0.025 (0.053) | 0.046 (0.054) | -0.050 (0.058) |
| *N* | 893 | 893 | 893 | 893 | 893 | 893 |
| *R*-squared | 0.003 | 0.009 | 0.008 | 0.041 | 0.026 | 0.025 |

Note. Cell entries are OLS regression coefficients with associated standard errors in parentheses. FT = feeling thermometer. * FDR adjusted *p* < .05. Angry writing is the reference group.

**Table H.4.14** The effects of interactions between emotion treatments and emotion strength on individual and subtractive affective polarization indicators (Study 4, US)

|  | FT outparty | FT outparty supporters | FT out-ideologues | Social distance | Trait ratings | Outgroup trust | FT party (subt.) | FT party supporters (subt.) | FT ideology (subt.) |
| --- | --- | --- | --- | --- | --- | --- | --- | --- | --- |
| Intercept | 0.251* (0.024) | 0.281* (0.023) | 0.361* (0.031) | 0.647* (0.023) | 0.424* (0.018) | 0.451* (0.023) | 0.780* (0.017) | 0.760* (0.017) | 0.698* (0.023) |
| Anxious Writing | 0.048 (0.034) | 0.058 (0.034) | 0.035 (0.043) | -0.035 (0.033) | 0.014 (0.027) | -0.028 (0.034) | -0.033 (0.024) | -0.030 (0.024) | -0.029 (0.032) |
| Happy Writing | 0.048 (0.035) | 0.044 (0.035) | 0.026 (0.046) | -0.029 (0.034) | -0.006 (0.028) | -0.004 (0.035) | -0.033 (0.025) | -0.017 (0.025) | -0.021 (0.034) |
| Emotion Strength | 0.011 (0.040) | 0.025 (0.040) | -0.032 (0.051) | -0.010 (0.039) | -0.008 (0.031) | -0.003 (0.040) | -0.027 (0.029) | -0.019 (0.028) | 0.015 (0.038) |
| Anxious Writing ×  Emotion Strength | -0.080 (0.056) | -0.097 (0.056) | -0.043 (0.071) | 0.007 (0.054) | -0.030 (0.044) | 0.011 (0.056) | 0.061 (0.040) | 0.051 (0.040) | 0.046 (0.053) |
| Happy Writing ×  Emotion Strength | -0.086 (0.060) | -0.054 (0.060) | -0.011 (0.076) | 0.037 (0.058) | 0.029 (0.047) | -0.013 (0.060) | 0.067 (0.043) | 0.022 (0.042) | 0.008 (0.057) |
| *N* | 893 | 893 | 665 | 893 | 893 | 893 | 893 | 893 | 665 |
| *R*-squared | 0.007 | 0.006 | 0.006 | 0.003 | 0.003 | 0.002 | 0.004 | 0.002 | 0.006 |

Note. Cell entries are OLS regression coefficients with associated standard errors in parentheses. FT = feeling thermometer. * FDR adjusted *p* < .05. Angry writing is the reference group.

**Table H.5.1** The effects of emotion treatments on affective polarization, feeling thermometers toward social groups, conspiracy endorsement, and deep fake recognition (Study 5, PL)

|  | Affective polarization | FT immigrants | FT feminists | FT neo-Nazis | FT nationalists | Conspiracy | Deep fake |
| --- | --- | --- | --- | --- | --- | --- | --- |
| Intercept | 0.310* (0.009) | 0.427* (0.016) | 0.466* (0.017) | 0.101* (0.012) | 0.266* (0.016) | 0.413* (0.011) | 0.271* (0.019) |
| Angry Writing | -0.001 (0.013) | -0.015 (0.023) | 0.012 (0.025) | 0.022 (0.017) | 0.039 (0.023) | 0.013 (0.016) | 0.057 (0.027) |
| Anxious Writing | 0.006 (0.013) | -0.021 (0.023) | 0.009 (0.025) | 0.002 (0.017) | 0.026 (0.023) | 0.003 (0.016) | 0.011 (0.027) |
| Happy Writing | 0.014 (0.014) | 0.001 (0.023) | 0.025 (0.026) | 0.034 (0.018) | 0.023 (0.024) | -0.008 (0.017) | 0.051 (0.028) |
| *N* | 1055 | 1055 | 1055 | 1055 | 1055 | 1055 | 1055 |
| *R*-squared | 0.001 | 0.001 | 0.001 | 0.005 | 0.003 | 0.002 | 0.006 |

Note. Cell entries are OLS regression coefficients with associated standard errors in parentheses. FT = feeling thermometer. * FDR adjusted *p* < .05.

**Table H.5.2** The effects of emotion treatments on individual and subtractive affective polarization indicators (Study 5, PL)

|  | FT outparty | FT outparty supporters | FT out-gov. | FT out-ideologues | Social distance | Trait ratings | Outgroup trust | FT party (subt.) | FT party supporters (subt.) | FT gov. (subt.) | FT ideology (subt.) |
| --- | --- | --- | --- | --- | --- | --- | --- | --- | --- | --- | --- |
| Intercept | 0.150* (0.013) | 0.186* (0.013) | 0.239* (0.014) | 0.318* (0.016) | 0.530* (0.014) | 0.398* (0.011) | 0.347* (0.013) | 0.772* (0.011) | 0.779* (0.011) | 0.720* (0.012) | 0.689* (0.013) |
| Angry Writing | 0.018 (0.019) | 0.002 (0.019) | 0.004 (0.020) | -0.008 (0.022) | -0.005 (0.021) | -0.006 (0.016) | -0.010 (0.019) | -0.020 (0.017) | -0.014 (0.015) | 0.008 (0.017) | 0.001 (0.018) |
| Anxious Writing | 0.006 (0.019) | -0.003 (0.019) | 0.018 (0.020) | 0.012 (0.023) | 0.000 (0.021) | 0.008 (0.016) | 0.008 (0.019) | 0.000 (0.017) | -0.009 (0.015) | -0.004 (0.017) | -0.018 (0.018) |
| Happy Writing | 0.027 (0.019) | 0.006 (0.020) | 0.020 (0.020) | 0.016 (0.023) | 0.018 (0.021) | 0.015 (0.017) | -0.001 (0.020) | -0.008 (0.017) | -0.006 (0.016) | -0.011 (0.018) | 0.005 (0.019) |
| *N* | 1055 | 1055 | 1055 | 790 | 1055 | 1055 | 1055 | 1055 | 1055 | 1055 | 790 |
| *R*-squared | 0.002 | 0.000 | 0.001 | 0.002 | 0.001 | 0.002 | 0.001 | 0.002 | 0.001 | 0.001 | 0.002 |

Note. Cell entries are OLS regression coefficients with associated standard errors in parentheses. FT = feeling thermometer. * FDR adjusted *p* < .05.

**Table H.5.3** The effects of interactions between emotion treatments and political identity strength on affective polarization, feeling thermometers toward social groups, conspiracy endorsement, and deep fake recognition (Study 5, PL)

|  | Affective polarization | FT immigrants | FT feminists | FT neo-Nazis | FT nationalists | Conspiracy | Deep fake |
| --- | --- | --- | --- | --- | --- | --- | --- |
| Intercept | 0.500* (0.025) | 0.394* (0.046) | 0.438* (0.050) | 0.193* (0.034) | 0.338* (0.047) | 0.439* (0.033) | 0.296* (0.056) |
| Angry Writing | -0.029 (0.037) | 0.085 (0.068) | 0.075 (0.075) | 0.037 (0.051) | 0.024 (0.071) | -0.032 (0.050) | 0.043 (0.083) |
| Anxious Writing | -0.018 (0.035) | 0.007 (0.065) | -0.080 (0.071) | -0.045 (0.048) | 0.038 (0.068) | -0.019 (0.047) | -0.087 (0.080) |
| Happy Writing | 0.023 (0.036) | 0.065 (0.067) | 0.030 (0.073) | -0.012 (0.050) | 0.046 (0.070) | 0.006 (0.049) | 0.072 (0.082) |
| Pids | -0.300* (0.037) | 0.052 (0.068) | 0.044 (0.074) | -0.146* (0.050) | -0.114 (0.070) | -0.041 (0.049) | -0.038 (0.083) |
| Angry Writing ×  Pids | 0.052 (0.054) | -0.155 (0.100) | -0.098 (0.109) | -0.019 (0.074) | 0.027 (0.104) | 0.071 (0.073) | 0.023 (0.122) |
| Anxious Writing ×  Pids | 0.032 (0.053) | -0.043 (0.098) | 0.145 (0.107) | 0.074 (0.073) | -0.021 (0.102) | 0.035 (0.071) | 0.156 (0.119) |
| Happy Writing ×  Pids | -0.010 (0.053) | -0.100 (0.099) | -0.007 (0.108) | 0.073 (0.073) | -0.035 (0.102) | -0.021 (0.072) | -0.033 (0.120) |
| *N* | 1055 | 1055 | 1055 | 1055 | 1055 | 1055 | 1055 |
| *R*-squared | 0.176 | 0.004 | 0.008 | 0.024 | 0.014 | 0.004 | 0.009 |

Note. Cell entries are OLS regression coefficients with associated standard errors in parentheses. FT = feeling thermometer. * FDR adjusted *p* < .05.

**Table H.5.4** The effects of interactions between emotion treatments and political identity strength on individual and subtractive affective polarization indicators (Study 5, PL)

|  | FT outparty | FT outparty supporters | FT out-gov. | FT out-ideologues | Social distance | Trait ratings | Outgroup trust | FT party (subt.) | FT party supporters (subt.) | FT gov. (subt.) | FT ideology (subt.) |
| --- | --- | --- | --- | --- | --- | --- | --- | --- | --- | --- | --- |
| Intercept | 0.327* (0.037) | 0.364* (0.038) | 0.513* (0.037) | 0.458* (0.046) | 0.736* (0.040) | 0.563* (0.031) | 0.513* (0.038) | 0.580* (0.031) | 0.598* (0.029) | 0.400* (0.029) | 0.512* (0.036) |
| Angry Writing | 0.026 (0.055) | -0.009 (0.057) | -0.051 (0.055) | -0.020 (0.066) | -0.157* (0.060) | -0.003 (0.047) | 0.015 (0.056) | -0.095 (0.046) | -0.061 (0.043) | 0.021 (0.044) | -0.010 (0.052) |
| Anxious Writing | -0.079 (0.052) | -0.067 (0.054) | 0.010 (0.052) | 0.007 (0.063) | 0.002 (0.058) | 0.036 (0.045) | -0.006 (0.054) | 0.014 (0.044) | 0.007 (0.041) | 0.032 (0.042) | 0.025 (0.049) |
| Happy Writing | 0.035 (0.054) | 0.009 (0.056) | -0.002 (0.054) | 0.091 (0.065) | 0.019 (0.059) | 0.047 (0.046) | 0.011 (0.055) | -0.043 (0.045) | -0.042 (0.042) | 0.007 (0.043) | -0.042 (0.051) |
| Pids | -0.280* (0.055) | -0.280* (0.056) | -0.433* (0.054) | -0.213* (0.066) | -0.326* (0.060) | -0.260* (0.046) | -0.263* (0.056) | 0.304* (0.046) | 0.285* (0.042) | 0.506* (0.044) | 0.270* (0.051) |
| Angry Writing ×  Pids | -0.004 (0.081) | 0.026 (0.083) | 0.098 (0.080) | 0.020 (0.094) | 0.244* (0.088) | 0.003 (0.068) | -0.031 (0.083) | 0.106 (0.067) | 0.063 (0.063) | -0.035 (0.065) | 0.013 (0.074) |
| Anxious Writing ×  Pids | 0.130 (0.079) | 0.099 (0.081) | 0.004 (0.079) | -0.005 (0.093) | -0.010 (0.087) | -0.052 (0.067) | 0.017 (0.081) | -0.016 (0.066) | -0.019 (0.061) | -0.046 (0.063) | -0.052 (0.072) |
| Happy Writing ×  Pids | -0.008 (0.079) | 0.000 (0.082) | 0.041 (0.079) | -0.112 (0.093) | 0.003 (0.087) | -0.047 (0.067) | -0.015 (0.082) | 0.051 (0.066) | 0.052 (0.062) | -0.035 (0.064) | 0.069 (0.073) |
| *N* | 1055 | 1055 | 1055 | 790 | 1055 | 1055 | 1055 | 1055 | 1055 | 1055 | 790 |
| *R*-squared | 0.074 | 0.066 | 0.161 | 0.066 | 0.077 | 0.118 | 0.076 | 0.164 | 0.158 | 0.296 | 0.133 |

Note. Cell entries are OLS regression coefficients with associated standard errors in parentheses. FT = feeling thermometer. * FDR adjusted *p* < .05.

**Table H.5.5** The effects of interactions between emotion treatments and political interest on affective polarization, feeling thermometers toward social groups, conspiracy endorsement, and deep fake recognition (Study 5, PL)

|  | Affective polarization | FT immigrants | FT feminists | FT neo-Nazis | FT nationalists | Conspiracy | Deep fake |
| --- | --- | --- | --- | --- | --- | --- | --- |
| Intercept | 0.367* (0.021) | 0.317* (0.036) | 0.422* (0.040) | 0.152* (0.027) | 0.267* (0.038) | 0.480* (0.027) | 0.366* (0.045) |
| Angry Writing | 0.029 (0.030) | -0.006 (0.052) | -0.005 (0.058) | 0.054 (0.039) | 0.132 (0.055) | -0.013 (0.038) | 0.001 (0.065) |
| Anxious Writing | 0.042 (0.031) | 0.005 (0.054) | -0.014 (0.059) | 0.035 (0.040) | 0.110 (0.056) | -0.017 (0.039) | -0.064 (0.066) |
| Happy Writing | 0.059 (0.033) | 0.099 (0.057) | 0.028 (0.062) | 0.050 (0.042) | 0.130 (0.059) | -0.085 (0.041) | -0.021 (0.070) |
| Interest | -0.099* (0.033) | 0.190* (0.057) | 0.076 (0.063) | -0.088 (0.043) | -0.002 (0.060) | -0.116* (0.042) | -0.165 (0.070) |
| Angry Writing ×  Interest | -0.051 (0.048) | -0.014 (0.082) | 0.030 (0.091) | -0.056 (0.062) | -0.161 (0.086) | 0.045 (0.060) | 0.097 (0.101) |
| Anxious Writing ×  Interest | -0.055 (0.048) | -0.051 (0.082) | 0.035 (0.091) | -0.051 (0.062) | -0.139 (0.086) | 0.037 (0.060) | 0.130 (0.101) |
| Happy Writing ×  Interest | -0.069 (0.051) | -0.171 (0.087) | -0.009 (0.096) | -0.023 (0.065) | -0.178 (0.091) | 0.132 (0.064) | 0.127 (0.107) |
| N | 1055 | 1055 | 1055 | 1055 | 1055 | 1055 | 1055 |
| R-squared | 0.062 | 0.025 | 0.008 | 0.032 | 0.021 | 0.015 | 0.012 |

Note. Cell entries are OLS regression coefficients with associated standard errors in parentheses. FT = feeling thermometer. * FDR adjusted *p* < .05.

**Table H.5.6** The effects of interactions between emotion treatments and political interest on individual and subtractive affective polarization indicators (Study 5, PL)

|  | FT outparty | FT outparty supporters | FT out-gov. | FT out-ideologues | Social distance | Trait ratings | Outgroup trust | FT party (subt.) | FT party supporters (subt.) | FT gov. (subt.) | FT ideology (subt.) |
| --- | --- | --- | --- | --- | --- | --- | --- | --- | --- | --- | --- |
| Intercept | 0.238* (0.030) | 0.247* (0.031) | 0.345* (0.032) | 0.332* (0.039) | 0.536* (0.034) | 0.459* (0.026) | 0.406* (0.031) | 0.625* (0.025) | 0.665* (0.024) | 0.572* (0.027) | 0.591* (0.031) |
| Angry Writing | 0.049 (0.043) | 0.047 (0.045) | -0.006 (0.045) | 0.077 (0.056) | 0.021 (0.048) | 0.014 (0.038) | 0.015 (0.045) | -0.051 (0.036) | -0.062 (0.034) | 0.008 (0.038) | -0.051 (0.044) |
| Anxious Writing | 0.030 (0.044) | 0.033 (0.046) | -0.002 (0.046) | 0.071 (0.058) | 0.085 (0.049) | 0.054 (0.039) | 0.042 (0.046) | 0.005 (0.037) | -0.005 (0.035) | 0.019 (0.039) | -0.014 (0.045) |
| Happy Writing | 0.076 (0.046) | 0.057 (0.048) | 0.047 (0.049) | 0.101 (0.063) | 0.090 (0.052) | 0.033 (0.041) | 0.036 (0.049) | -0.037 (0.039) | -0.049 (0.037) | -0.040 (0.041) | -0.061 (0.049) |
| Interest | -0.152* (0.047) | -0.105 (0.049) | -0.184* (0.049) | -0.023 (0.059) | -0.012 (0.053) | -0.105 (0.041) | -0.102 (0.049) | 0.254* (0.039) | 0.197* (0.037) | 0.257* (0.042) | 0.162* (0.046) |
| Angry Writing ×  Interest | -0.055 (0.068) | -0.078 (0.070) | 0.018 (0.071) | -0.139 (0.085) | -0.045 (0.076) | -0.035 (0.059) | -0.045 (0.071) | 0.054 (0.057) | 0.084 (0.053) | 0.001 (0.060) | 0.082 (0.066) |
| Anxious Writing ×  Interest | -0.033 (0.068) | -0.055 (0.070) | 0.040 (0.071) | -0.093 (0.086) | -0.141 (0.076) | -0.072 (0.059) | -0.052 (0.071) | -0.019 (0.057) | -0.015 (0.053) | -0.048 (0.060) | -0.012 (0.068) |
| Happy Writing ×  Interest | -0.073 (0.071) | -0.079 (0.074) | -0.035 (0.075) | -0.131 (0.093) | -0.119 (0.080) | -0.025 (0.063) | -0.056 (0.075) | 0.036 (0.060) | 0.062 (0.056) | 0.035 (0.064) | 0.094 (0.073) |
| *N* | 1055 | 1055 | 1055 | 790 | 1055 | 1055 | 1055 | 1055 | 1055 | 1055 | 790 |
| *R*-squared | 0.057 | 0.035 | 0.044 | 0.021 | 0.014 | 0.040 | 0.028 | 0.143 | 0.120 | 0.114 | 0.083 |

Note. Cell entries are OLS regression coefficients with associated standard errors in parentheses. FT = feeling thermometer. * FDR adjusted *p* < .05.

**Table H.5.7** The effects of interactions between emotion treatments and attitude toward the government on affective polarization, feeling thermometers toward social groups, conspiracy endorsement, and deep fake recognition (Study 5, PL)

|  | Affective polarization | FT immigrants | FT feminists | FT neo-Nazis | FT nationalists | Conspiracy | Deep fake |
| --- | --- | --- | --- | --- | --- | --- | --- |
| Intercept | 0.285* (0.011) | 0.478* (0.019) | 0.526* (0.020) | 0.078* (0.014) | 0.177* (0.018) | 0.378* (0.014) | 0.251* (0.024) |
| Angry Writing | -0.001 (0.017) | -0.003 (0.028) | 0.044 (0.030) | 0.008 (0.021) | 0.026 (0.027) | 0.001 (0.020) | 0.085 (0.035) |
| Anxious Writing | -0.008 (0.017) | -0.031 (0.028) | 0.030 (0.030) | -0.016 (0.021) | -0.001 (0.027) | -0.004 (0.020) | 0.032 (0.035) |
| Happy Writing | -0.005 (0.017) | 0.007 (0.029) | 0.048 (0.031) | 0.004 (0.022) | 0.025 (0.028) | -0.028 (0.021) | 0.038 (0.037) |
| Att. Gov. (Supporter) | 0.071* (0.019) | -0.145* (0.032) | -0.169* (0.034) | 0.066* (0.024) | 0.250* (0.031) | 0.100* (0.023) | 0.056 (0.040) |
| Angry Writing ×  Att. Gov.  (Supporter) | -0.010 (0.027) | -0.006 (0.045) | -0.051 (0.048) | 0.023 (0.034) | -0.004 (0.044) | 0.013 (0.032) | -0.075 (0.056) |
| Anxious Writing ×  Att. Gov.  (Supporter) | 0.022 (0.026) | 0.049 (0.045) | -0.021 (0.048) | 0.033 (0.034) | 0.022 (0.043) | 0.000 (0.032) | -0.059 (0.056) |
| Happy Writing ×  Att. Gov.  (Supporter) | 0.032 (0.027) | 0.013 (0.047) | -0.023 (0.050) | 0.058 (0.035) | -0.049 (0.045) | 0.029 (0.033) | 0.019 (0.058) |
| *N* | 1055 | 1055 | 1055 | 1055 | 1055 | 1055 | 1055 |
| *R*-squared | 0.069 | 0.062 | 0.107 | 0.060 | 0.191 | 0.080 | 0.011 |

Note. Cell entries are OLS regression coefficients with associated standard errors in parentheses. FT = feeling thermometer. * FDR adjusted *p* < .05.

**Table H.5.8** The effects of interactions between emotion treatments and attitude toward the government on individual and subtractive affective polarization indicators (Study 5, PL)

|  | FT outparty | FT outparty supporters | FT out-gov. | FT out-ideologues | Social distance | Trait ratings | Outgroup trust | FT party (subt.) | FT party supporters (subt.) | FT gov. (subt.) | FT ideology (subt.) |
| --- | --- | --- | --- | --- | --- | --- | --- | --- | --- | --- | --- |
| Intercept | 0.133* (0.016) | 0.162* (0.016) | 0.189* (0.016) | 0.327* (0.021) | 0.504* (0.018) | 0.368* (0.014) | 0.317* (0.016) | 0.786* (0.014) | 0.791* (0.013) | 0.746* (0.015) | 0.683* (0.017) |
| Angry Writing | 0.017 (0.024) | -0.008 (0.024) | 0.020 (0.024) | -0.034 (0.030) | -0.006 (0.026) | -0.008 (0.020) | 0.009 (0.024) | -0.029 (0.021) | -0.010 (0.019) | -0.005 (0.022) | 0.009 (0.024) |
| Anxious Writing | -0.010 (0.024) | -0.017 (0.024) | 0.008 (0.024) | -0.007 (0.031) | 0.005 (0.026) | -0.014 (0.020) | -0.021 (0.024) | 0.009 (0.021) | 0.009 (0.020) | 0.018 (0.022) | 0.003 (0.025) |
| Happy Writing | 0.002 (0.025) | -0.020 (0.025) | -0.001 (0.025) | -0.035 (0.032) | 0.010 (0.027) | 0.001 (0.021) | -0.005 (0.025) | -0.002 (0.022) | 0.009 (0.020) | -0.008 (0.023) | 0.030 (0.026) |
| Att. Gov. (Supporter) | 0.050 (0.027) | 0.068 (0.027) | 0.141* (0.027) | -0.022 (0.032) | 0.073 (0.030) | 0.087* (0.023) | 0.084* (0.027) | -0.039 (0.024) | -0.034 (0.022) | -0.074* (0.024) | 0.014 (0.026) |
| Angry Writing ×  Att. Gov.  (Supporter) | -0.005 (0.038) | 0.013 (0.039) | -0.057 (0.039) | 0.059 (0.045) | -0.009 (0.042) | -0.008 (0.033) | -0.058 (0.039) | 0.027 (0.034) | -0.003 (0.031) | 0.042 (0.035) | -0.019 (0.037) |
| Anxious Writing ×  Att. Gov.  (Supporter) | 0.029 (0.038) | 0.023 (0.039) | -0.002 (0.039) | 0.041 (0.046) | -0.025 (0.042) | 0.037 (0.032) | 0.054 (0.039) | -0.014 (0.034) | -0.037 (0.031) | -0.038 (0.035) | -0.046 (0.037) |
| Happy Writing ×  Att. Gov.  (Supporter) | 0.050 (0.040) | 0.050 (0.040) | 0.026 (0.040) | 0.106 (0.047) | 0.005 (0.044) | 0.017 (0.034) | -0.004 (0.040) | -0.007 (0.035) | -0.030 (0.032) | 0.005 (0.036) | -0.054 (0.038) |
| *N* | 1055 | 1055 | 1055 | 790 | 1055 | 1055 | 1055 | 1055 | 1055 | 1055 | 790 |
| *R*-squared | 0.026 | 0.038 | 0.083 | 0.012 | 0.019 | 0.066 | 0.040 | 0.012 | 0.022 | 0.036 | 0.007 |

Note. Cell entries are OLS regression coefficients with associated standard errors in parentheses. FT = feeling thermometer. * FDR adjusted *p* < .05.

**Table H.5.9** The effects of interactions between emotion treatments and ideology on affective polarization, feeling thermometers toward social groups, conspiracy endorsement, and deep fake recognition (Study 5, PL)

|  | Affective polarization | FT immigrants | FT feminists | FT neo-Nazis | FT nationalists | Conspiracy | Deep fake |
| --- | --- | --- | --- | --- | --- | --- | --- |
| Intercept | 0.291* (0.018) | 0.531* (0.031) | 0.649* (0.032) | 0.058 (0.023) | 0.073 (0.030) | 0.327* (0.022) | 0.249* (0.038) |
| Angry Writing | -0.019 (0.026) | -0.005 (0.044) | 0.025 (0.046) | 0.009 (0.033) | 0.039 (0.043) | 0.032 (0.031) | 0.044 (0.055) |
| Anxious Writing | -0.033 (0.027) | -0.007 (0.045) | 0.002 (0.047) | -0.029 (0.034) | -0.023 (0.043) | -0.009 (0.032) | 0.012 (0.056) |
| Happy Writing | -0.010 (0.028) | 0.035 (0.046) | 0.032 (0.048) | -0.023 (0.035) | 0.020 (0.044) | -0.048 (0.033) | -0.023 (0.057) |
| Ideology | 0.037 (0.030) | -0.198* (0.050) | -0.347* (0.053) | 0.081 (0.038) | 0.365* (0.049) | 0.164* (0.036) | 0.043 (0.063) |
| Angry Writing ×  Ideology | 0.033 (0.043) | -0.015 (0.071) | -0.019 (0.075) | 0.024 (0.054) | -0.004 (0.069) | -0.038 (0.051) | 0.023 (0.089) |
| Anxious Writing ×  Ideology | 0.077 (0.045) | -0.033 (0.075) | 0.004 (0.079) | 0.064 (0.057) | 0.106 (0.073) | 0.029 (0.054) | -0.002 (0.094) |
| Happy Writing ×  Ideology | 0.044 (0.045) | -0.057 (0.074) | -0.004 (0.078) | 0.103 (0.056) | -0.004 (0.072) | 0.071 (0.053) | 0.135 (0.093) |
| *N* | 1055 | 1055 | 1055 | 1055 | 1055 | 1055 | 1055 |
| *R*-squared | 0.024 | 0.067 | 0.136 | 0.045 | 0.185 | 0.083 | 0.014 |

Note. Cell entries are OLS regression coefficients with associated standard errors in parentheses. FT = feeling thermometer. * FDR adjusted *p* < .05.

**Table H.5.10** The effects of interactions between emotion treatments and ideology on individual and subtractive affective polarization indicators (Study 5, PL)

|  | FT outparty | FT outparty supporters | FT out-gov. | FT out-ideologues | Social distance | Trait ratings | Outgroup trust | FT party (subt.) | FT party supporters (subt.) | FT gov. (subt.) | FT ideology (subt.) |
| --- | --- | --- | --- | --- | --- | --- | --- | --- | --- | --- | --- |
| Intercept | 0.145* (0.026) | 0.154* (0.027) | 0.180* (0.027) | 0.313* (0.029) | 0.518* (0.029) | 0.398* (0.023) | 0.323* (0.027) | 0.780* (0.023) | 0.799* (0.021) | 0.753* (0.024) | 0.712* (0.023) |
| Angry Writing | -0.012 (0.038) | -0.015 (0.038) | -0.006 (0.039) | -0.031 (0.041) | -0.034 (0.041) | -0.032 (0.032) | -0.002 (0.038) | -0.017 (0.033) | -0.018 (0.031) | 0.019 (0.034) | -0.032 (0.033) |
| Anxious Writing | -0.030 (0.038) | -0.025 (0.039) | 0.007 (0.040) | 0.028 (0.042) | -0.073 (0.042) | -0.071 (0.033) | -0.060 (0.039) | 0.022 (0.034) | 0.018 (0.031) | 0.037 (0.035) | -0.036 (0.034) |
| Happy Writing | -0.013 (0.039) | -0.016 (0.040) | 0.009 (0.041) | -0.012 (0.043) | -0.015 (0.043) | -0.026 (0.034) | 0.003 (0.040) | -0.007 (0.034) | -0.012 (0.032) | -0.040 (0.036) | -0.002 (0.035) |
| Ideology | 0.009 (0.043) | 0.061 (0.044) | 0.112 (0.045) | 0.009 (0.045) | 0.021 (0.047) | 0.001 (0.037) | 0.045 (0.044) | -0.016 (0.038) | -0.039 (0.035) | -0.063 (0.040) | -0.042 (0.036) |
| Angry Writing ×  Ideology | 0.055 (0.061) | 0.031 (0.063) | 0.017 (0.063) | 0.041 (0.063) | 0.055 (0.067) | 0.048 (0.053) | -0.016 (0.063) | -0.005 (0.054) | 0.009 (0.050) | -0.020 (0.056) | 0.060 (0.051) |
| Anxious Writing ×  Ideology | 0.071 (0.064) | 0.045 (0.066) | 0.024 (0.067) | -0.030 (0.066) | 0.144 (0.070) | 0.152* (0.055) | 0.134 (0.066) | -0.043 (0.057) | -0.053 (0.052) | -0.081 (0.059) | 0.033 (0.054) |
| Happy Writing ×  Ideology | 0.074 (0.063) | 0.039 (0.065) | 0.018 (0.066) | 0.050 (0.066) | 0.061 (0.070) | 0.076 (0.055) | -0.008 (0.065) | -0.001 (0.056) | 0.012 (0.052) | 0.054 (0.058) | 0.014 (0.053) |
| *N* | 1055 | 1055 | 1055 | 790 | 1055 | 1055 | 1055 | 1055 | 1055 | 1055 | 790 |
| *R*-squared | 0.010 | 0.015 | 0.029 | 0.005 | 0.016 | 0.020 | 0.015 | 0.004 | 0.009 | 0.018 | 0.005 |

Note. Cell entries are OLS regression coefficients with associated standard errors in parentheses. FT = feeling thermometer. * FDR adjusted *p* < .05.

**Table H.5.11** The effects of interactions between emotion treatments and emotion strength on affective polarization, feeling thermometers toward social groups, conspiracy endorsement, and deep fake recognition (Study 5, PL)

|  | Affective polarization | FT immigrants | FT feminists | FT neo-Nazis | FT nationalists | Conspiracy | Deep fake |
| --- | --- | --- | --- | --- | --- | --- | --- |
| Intercept | 0.331* (0.017) | 0.382* (0.028) | 0.433* (0.031) | 0.215* (0.021) | 0.313* (0.029) | 0.413* (0.020) | 0.342* (0.035) |
| Angry Writing | -0.011 (0.021) | 0.013 (0.036) | 0.016 (0.040) | -0.072* (0.027) | 0.005 (0.038) | 0.019 (0.026) | 0.021 (0.045) |
| Anxious Writing | -0.008 (0.021) | -0.003 (0.035) | 0.012 (0.038) | -0.097* (0.026) | -0.012 (0.036) | 0.022 (0.025) | -0.062 (0.043) |
| Emotion Strength | -0.014 (0.026) | 0.091 (0.043) | 0.116 (0.048) | -0.160* (0.033) | -0.049 (0.045) | -0.016 (0.031) | -0.040 (0.054) |
| Angry Writing ×  Emotion Strength | -0.015 (0.037) | -0.044 (0.061) | -0.037 (0.068) | 0.104 (0.047) | 0.014 (0.064) | 0.002 (0.044) | -0.057 (0.076) |
| Anxious Writing ×  Emotion Strength | -0.010 (0.037) | -0.002 (0.061) | -0.013 (0.068) | 0.110 (0.047) | 0.020 (0.064) | -0.048 (0.044) | 0.047 (0.077) |
| *N* | 763 | 763 | 763 | 763 | 763 | 763 | 763 |
| *R*-squared | 0.005 | 0.014 | 0.018 | 0.041 | 0.003 | 0.008 | 0.009 |

Note. Cell entries are OLS regression coefficients with associated standard errors in parentheses. FT = feeling thermometer. * FDR adjusted *p* < .05. Happy writing is the reference group.

**Table H.5.12** The effects of interactions between emotion treatments and emotion strength on individual and subtractive affective polarization indicators (Study 5, PL)

|  | FT outparty | FT outparty supporters | FT out-gov. | FT out-ideologues | Social distance | Trait ratings | Outgroup trust | FT party (subt.) | FT party supporters (subt.) | FT gov. (subt.) | FT ideology (subt.) |
| --- | --- | --- | --- | --- | --- | --- | --- | --- | --- | --- | --- |
| Intercept | 0.230* (0.024) | 0.226* (0.024) | 0.278* (0.024) | 0.329* (0.028) | 0.504* (0.026) | 0.402* (0.020) | 0.349* (0.024) | 0.717* (0.020) | 0.738* (0.019) | 0.682* (0.021) | 0.668* (0.022) |
| Angry Writing | -0.050 (0.030) | -0.022 (0.031) | -0.018 (0.031) | 0.004 (0.036) | 0.019 (0.033) | 0.002 (0.026) | -0.002 (0.031) | 0.020 (0.026) | 0.010 (0.024) | 0.025 (0.027) | 0.009 (0.029) |
| Anxious Writing | -0.056 (0.029) | -0.034 (0.030) | -0.013 (0.030) | 0.003 (0.035) | 0.019 (0.032) | 0.009 (0.025) | 0.024 (0.030) | 0.036 (0.025) | 0.016 (0.024) | 0.013 (0.026) | -0.004 (0.028) |
| Emotion Strength | -0.105* (0.037) | -0.067 (0.037) | -0.037 (0.038) | 0.010 (0.044) | 0.087 (0.040) | 0.024 (0.031) | -0.006 (0.037) | 0.094* (0.032) | 0.070 (0.030) | 0.054 (0.033) | 0.052 (0.035) |
| Angry Writing ×  Emotion Strength | 0.071 (0.052) | 0.024 (0.053) | -0.010 (0.054) | -0.075 (0.062) | -0.081 (0.056) | -0.056 (0.044) | -0.022 (0.053) | -0.052 (0.045) | -0.021 (0.042) | 0.005 (0.046) | -0.016 (0.050) |
| Anxious Writing ×  Emotion Strength | 0.043 (0.052) | 0.037 (0.053) | 0.010 (0.054) | -0.016 (0.062) | -0.063 (0.056) | -0.038 (0.044) | -0.056 (0.053) | -0.029 (0.045) | -0.015 (0.042) | 0.021 (0.046) | -0.029 (0.050) |
| *N* | 763 | 763 | 763 | 579 | 763 | 763 | 763 | 763 | 763 | 763 | 579 |
| *R*-squared | 0.017 | 0.007 | 0.005 | 0.006 | 0.008 | 0.004 | 0.005 | 0.021 | 0.016 | 0.016 | 0.009 |

Note. Cell entries are OLS regression coefficients with associated standard errors in parentheses. FT = feeling thermometer. * FDR adjusted *p* < .05. Happy writing is the reference group.

**Table H.5.13** The effects of interactions between emotion treatments and emotion strength on affective polarization, feeling thermometers toward social groups, conspiracy endorsement, and deep fake recognition (Study 5, PL)

|  | Affective polarization | FT immigrants | FT feminists | FT neo-Nazis | FT nationalists | Conspiracy | Deep fake |
| --- | --- | --- | --- | --- | --- | --- | --- |
| Intercept | 0.320* (0.013) | 0.395* (0.023) | 0.449* (0.025) | 0.143* (0.017) | 0.318* (0.024) | 0.432* (0.016) | 0.363* (0.028) |
| Anxious Writing | 0.004 (0.018) | -0.015 (0.031) | -0.005 (0.034) | -0.025 (0.023) | -0.017 (0.032) | 0.003 (0.022) | -0.083 (0.038) |
| Happy Writing | 0.011 (0.021) | -0.013 (0.036) | -0.016 (0.040) | 0.072* (0.027) | -0.005 (0.038) | -0.019 (0.026) | -0.021 (0.045) |
| Emotion Strength | -0.029 (0.026) | 0.047 (0.043) | 0.080 (0.048) | -0.056 (0.033) | -0.035 (0.046) | -0.014 (0.031) | -0.097 (0.054) |
| Anxious Writing ×  Emotion Strength | 0.005 (0.037) | 0.042 (0.061) | 0.024 (0.068) | 0.005 (0.047) | 0.006 (0.065) | -0.049 (0.044) | 0.104 (0.077) |
| Happy Writing ×  Emotion Strength | 0.015 (0.037) | 0.044 (0.061) | 0.037 (0.068) | -0.104 (0.047) | -0.014 (0.064) | -0.002 (0.044) | 0.057 (0.076) |
| N | 763 | 763 | 763 | 763 | 763 | 763 | 763 |
| R-squared | 0.005 | 0.014 | 0.018 | 0.041 | 0.003 | 0.008 | 0.009 |

Note. Cell entries are OLS regression coefficients with associated standard errors in parentheses. FT = feeling thermometer. * FDR adjusted *p* < .05. Angry writing is the reference group.

**Table H.5.14** The effects of interactions between emotion treatments and emotion strength on individual and subtractive affective polarization indicators (Study 5, PL)

|  | FT outparty | FT outparty supporters | FT out-gov. | FT out-ideologues | Social distance | Trait ratings | Outgroup trust | FT party (subt.) | FT party supporters (subt.) | FT gov. (subt.) | FT ideology (subt.) |
| --- | --- | --- | --- | --- | --- | --- | --- | --- | --- | --- | --- |
| Intercept | 0.180* (0.019) | 0.204* (0.019) | 0.260* (0.020) | 0.333* (0.023) | 0.522* (0.021) | 0.403* (0.016) | 0.347* (0.020) | 0.737* (0.017) | 0.747* (0.015) | 0.707* (0.017) | 0.677* (0.018) |
| Anxious Writing | -0.006 (0.026) | -0.011 (0.026) | 0.004 (0.027) | -0.001 (0.032) | 0.000 (0.028) | 0.007 (0.022) | 0.026 (0.026) | 0.016 (0.023) | 0.006 (0.021) | -0.012 (0.023) | -0.013 (0.025) |
| Happy Writing | 0.050 (0.030) | 0.022 (0.031) | 0.018 (0.031) | -0.004 (0.036) | -0.019 (0.033) | -0.002 (0.026) | 0.002 (0.031) | -0.020 (0.026) | -0.010 (0.024) | -0.025 (0.027) | -0.009 (0.029) |
| Emotion Strength | -0.034 (0.037) | -0.042 (0.037) | -0.047 (0.038) | -0.064 (0.044) | 0.007 (0.040) | -0.031 (0.031) | -0.028 (0.038) | 0.042 (0.032) | 0.049 (0.030) | 0.059 (0.033) | 0.036 (0.035) |
| Anxious Writing ×  Emotion Strength | -0.028 (0.052) | 0.013 (0.053) | 0.020 (0.054) | 0.059 (0.063) | 0.017 (0.056) | 0.018 (0.044) | -0.034 (0.053) | 0.024 (0.045) | 0.006 (0.042) | 0.016 (0.046) | -0.014 (0.050) |
| Happy Writing ×  Emotion Strength | -0.071 (0.052) | -0.024 (0.053) | 0.010 (0.054) | 0.075 (0.062) | 0.081 (0.056) | 0.056 (0.044) | 0.022 (0.053) | 0.052 (0.045) | 0.021 (0.042) | -0.005 (0.046) | 0.016 (0.050) |
| *N* | 763 | 763 | 763 | 579 | 763 | 763 | 763 | 763 | 763 | 763 | 579 |
| *R*-squared | 0.017 | 0.007 | 0.005 | 0.006 | 0.008 | 0.004 | 0.005 | 0.021 | 0.016 | 0.016 | 0.009 |

Note. Cell entries are OLS regression coefficients with associated standard errors in parentheses. FT = feeling thermometer. * FDR adjusted *p* < .05. Angry writing is the reference group.

**Appendix I: Stimuli for control conditions and the manipulation check item (Study 3)**

[Control writing condition]

Now we would like you to describe what you are seeing in your surroundings right now. It may be your living room, office, a park, or a café.

[Control questions condition]

What is the brand of your laptop?

What did you have for breakfast?

What time do you typically get up?

What’s the color of your shoes?

Which country in the Europe has the largest population?

How many days a week do you do groceries?

[Control photos condition]


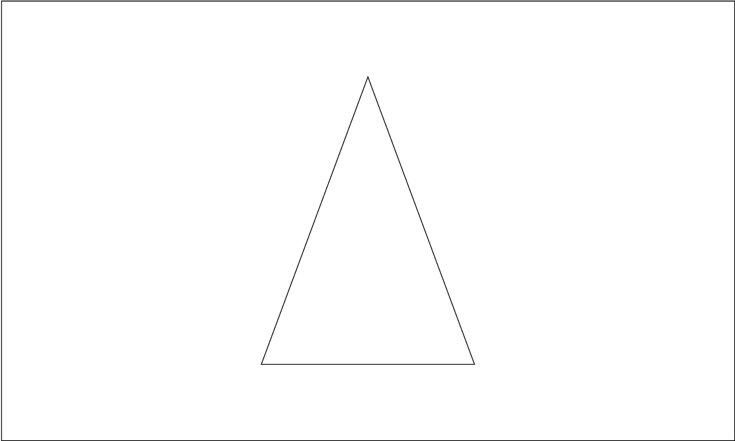


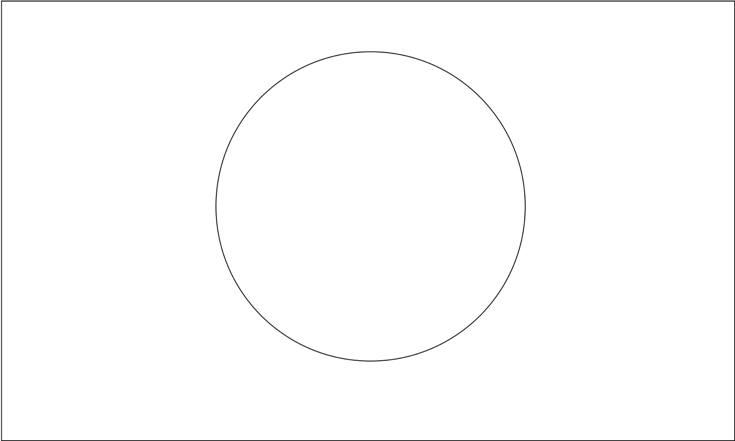


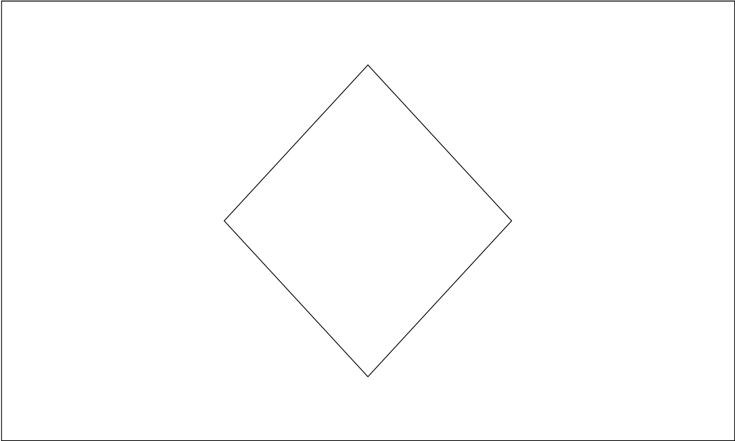


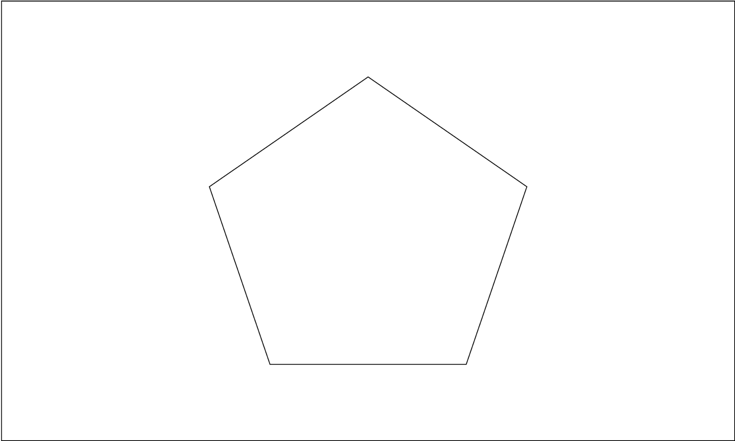


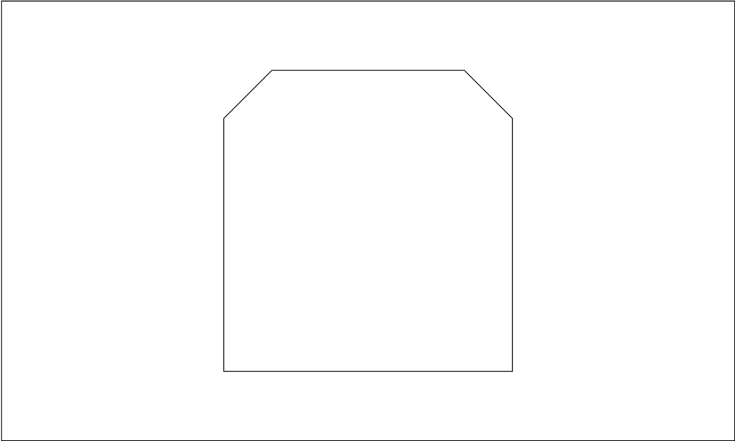


[Manipulation check (SAM)] **
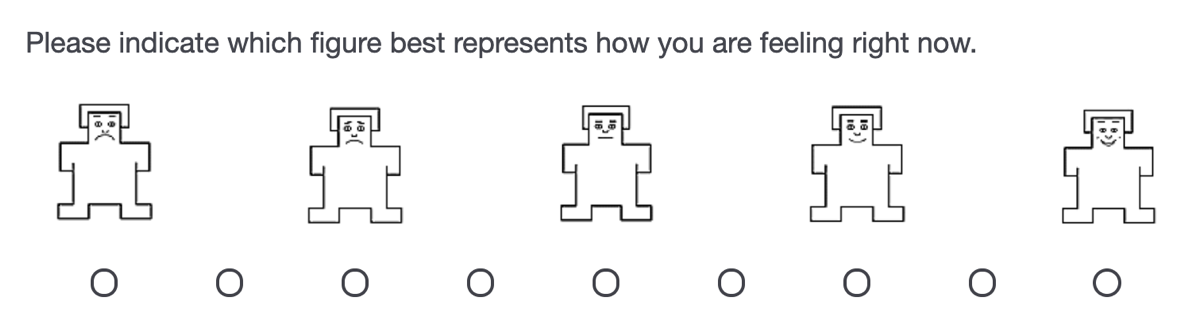
**
